# Whole-Transcriptome Sequencing-Based Analysis of *DAZL* and Its Interacting Genes during Germ Cells Specification and Zygotic Genome Activation in Chickens

**DOI:** 10.3390/ijms21218170

**Published:** 2020-10-31

**Authors:** Deivendran Rengaraj, Sohyoung Won, Jong Won Han, DongAhn Yoo, Heebal Kim, Jae Yong Han

**Affiliations:** 1 Department of Agricultural Biotechnology, and Research Institute of Agriculture and Life Sciences, Seoul National University, Seoul 08826, Korea; deivendran@snu.ac.kr (D.R.); hanjo106@snu.ac.kr (J.W.H.); heebal@snu.ac.kr (H.K.); 2 Interdisciplinary Program in Bioinformatics, Seoul National University, Seoul 08826, Korea; wsy415@snu.ac.kr (S.W.); day1092@snu.ac.kr (D.Y.); 3 C&K Genomics, Seoul 05836, Korea

**Keywords:** *DAZL*, *DAZL* interacting genes, germ cells development, intrauterine embryos, zygotic genome activation

## Abstract

The deleted in azoospermia like (*DAZL*) is required for germ cells development and maintenance. In chickens, the mRNA and protein of *DAZL*, a representative maternally inherited germ plasm factor, are detected in the germ plasm of oocyte, zygote, and all stages of the intrauterine embryos. However, it is still insufficient to explain the origin and specification process of chicken germ cells, because the stage at which the zygotic transcription of *DAZL* occurs and the stage at which the maternal *DAZL* RNA/protein clears have not yet been fully identified. Moreover, a comprehensive understanding of the expression of *DAZL* interacting genes during the germ cells specification and development and zygotic genome activation (ZGA) is lacking in chickens. In this study, we identified a set of *DAZL* interacting genes in chickens using in silico prediction method. Then, we analyzed the whole-transcriptome sequencing (WTS)-based expression of *DAZL* and its interacting genes in the chicken oocyte, zygote, and Eyal-Giladi and Kochav (EGK) stage embryos (EGK.I to EGK.X). In the results, *DAZL* transcripts are increased in the zygote (onset of transcription), maintained the increased level until EGK.VI, and decreased from EGK.VIII (possible clearance of maternal RNAs). Among the *DAZL* interacting genes, most of them are increased either at 1st ZGA or 2nd ZGA, indicating their involvement in germ cells specification and development.

## 1. Introduction

During fertilization, the haploid egg (oocyte) nucleus fuses with the sperm nucleus to produce a diploid one-cell embryo (zygote). The zygote is defined as totipotent due to its remarkable potential to develop into an embryo with all the specialized cells that make up a living organism [1]. When the zygote undergoes further embryonic development, primordial germ cells (PGCs, as the precursor of germ cells) are developed along with other types of specialized cells. Two modes of germ cells specification and development occur in animals: induction mode and preformation mode. The induction mode has been well explained or predicted in species, such as mice and several mammals, turtles, salamander, skates, and sharks [2]. In the mice and humans, signals from extraembryonic tissues induce a unique gene regulatory network in germline-competent cells for PGC specification, and PGCs are established in perigastrulation-stage embryos [3]. The preformation mode has been well explained or predicted in species, such as fruit flies, frogs, zebrafish, roundworm, sturgeon, and ascidians [2,4]. In the fruit flies, frogs, and zebrafish, the maternally stored germ plasm (containing RNAs, proteins, and energy-rich mitochondria) in the oocyte is inherited into the cells specifying precursor germ cells during early embryonic development [5,6,7]. Commonly in both modes, germ cells specification requires the expression of germ plasm factors and the precise interaction of germ cells-related genes. 

The deleted in azoospermia like (DAZL), an RNA-binding protein, has been identified in diverse vertebrate and invertebrate species, including chickens, as a master factor for the germ cells development and maintenance [8,9]. The homozygous deletion of *DAZL* gene causes a complete loss of germ cells in mouse testis and ovary [10]. Another study indicates that the germ cells specification and migration are not affected in *DAZL*-deficient mouse fetuses; however, the PGCs do not enter into further stages of germ cells development in both male and female gonads [11]. Moreover, it is demonstrated that the *DAZL* is necessary to restrict the developmental potential of the germline cells in gonads. *DAZL*’s absence prolongs the expression of a *NANOG* pluripotency reporter, causing spontaneous gonadal teratomas in mice and pigs [12]. In mammals, *DAZL* interacts with thousands of genes as a master regulator of germ cells gene expression. Due to its RNA-binding properties, DAZL protein binds with the 3′ untranslated region (UTR) of target mRNAs at the GUU-enriched regions [13]. DAZL enhances the translation of genes critically required for normal functioning of germ cells at various stages, including germ cells specification, development, and differentiation [13,14,15,16,17]. In addition, DAZL acts as a translational repressor of core pluripotency-, somatic differentiation-, and apoptosis-related genes in nascent PGCs [14], protecting its survivability and ability to differentiate into germline cells (spermatogonia/oogonia).

In chickens, the mRNA transcripts and proteins of *DAZL* are detected in the germ plasm of oocyte and zygote as maternally stored factors [18]. When the zygote undergoes intrauterine embryonic development, as described by Eyal-Giladi and Kochav (EGK) stages [19,20], the expression of *DAZL* is localized in the cleavage furrows at EGK.I. Then, the expression of *DAZL* is localized in several central cells at EGK.III to EGK.X [18]. This expression pattern analysis of *DAZL* might reveal its importance in germ cells specification and development during the chicken intrauterine embryonic development. The chicken intrauterine embryonic development has other important features, such as zygotic genome activation (ZGA) and maternal-to-zygotic transition (MZT). During ZGA, the maternal RNAs and proteins stored in the cytoplasm of zygote activates zygotic transcriptions and controls the initial development of embryos. During MZT, maternal RNAs and proteins are cleared and the zygotic RNAs and proteins control the further development of embryos [21]. In chickens, two waves of ZGA have been revealed by transcriptome sequencing during intrauterine embryonic development: 1st ZGA (a minor wave) occurs in the zygote shortly after fertilization and 2nd ZGA (a major wave) occurs between EGK.III and EGK.VI shortly before MZT [21,22,23]. Moreover, to avoid transcription from supernumerary sperm nuclei, the maternal genome is activated during 1st ZGA, and the paternal genome is quiescent until 2nd ZGA in the chickens [21,24].

Although the localization of *DAZL* mRNAs is detected at all stages of chicken intrauterine embryonic development [18], it is still insufficient to explain the origin and specification process of chicken germ cells, because it is not very clear at which stage the zygotic transcription of *DAZL* occurs and at which stage the maternal RNA/protein of *DAZL* clears. Moreover, a comprehensive understanding of the *DAZL* interacting genes and their expression patterns during the germ cells specification/development and ZGA is lacking in chickens. To uncover the above themes, we identified a set of *DAZL* interacting genes in chickens using in silico prediction method. Then, we analyzed the whole-transcriptome sequencing (WTS)-based expression patterns of the *DAZL* gene and *DAZL* interacting genes in the chicken oocyte, zygote, and intrauterine embryos (EGK.I to EGK.X).

## 2. Results and Discussion

### 2.1. Prediction of DAZL Interacting Genes and Motifs Analysis

In this study, the *DAZL* interacting genes in chickens are first predicted using the search tool for the retrieval of interacting genes/proteins (STRING) database (v. 11.0). The STRING database is helpful to predict the direct (physical) and indirect (functional) associations of genes/proteins, and that information comes from computational prediction, knowledge transfers between organisms, and interactions aggregated in other primary databases [25]. According to medium to highest confidence score setting (score: 0.4–1.0), the STRING database predicted 139 genes that have direct associations with *DAZL*. Of the 139 genes, 13 genes, including *DND1*, *STRA8*, *DDX4*, *NANOS3*, *SYCP3*, *PUM2*, *PRDM14*, *DZIP1*, *TDRD7*, *PUM1*, *POUV*, *NANOG*, and *NANOS1* are predicted with high to highest confidence scores (score: 0.7–1.0) (Table S1). To further confirm the *DAZL* interaction, 3’UTR sequences of all the predicted *DAZL* interacting genes, along with the *DAZL* gene, are extracted from the chicken (galGal6a) reference genome and analyzed for the presence of DAZL protein binding motifs via an in-house Python script (Supplementary Material).

In chickens, a precise analysis of *DAZL* interacting genes is not conducted experimentally. Therefore, we analyzed the *DAZL* interacting genes in this study based on the conserved DAZL binding motifs reported in other species. In the results, we identified different counts of the reported DAZL binding motifs, such as UGUU(U/A) [17], UGUU [16], GUU(U/A) [17], GUUG [13], GUUC [26], and UUU(C/G)UUU [15,27], in the 3’UTR of the predicted *DAZL* interacting genes in chicken (Table S1 and Figure S1). Notably, the UGUU(U/A) motif is identified in most predicted *DAZL* interacting genes. This motif is recently reported as the most preferential motif for DAZL binding than all other motifs mentioned above [17]. Moreover, the longer 3’UTRs contained more counts, and the shorter 3’UTRs contained fewer counts of the DAZL binding motifs. We could not identify DAZL binding motifs in a few chicken genes, in which the 3’UTR is not well-annotated. Importantly, DAZL binding motifs are also identified in the 3’UTR sequences of the *DAZL* gene, indicating that the *DAZL* involves promoting its translation. A similar observation of *DAZL* promoting its translation is reported in an earlier in vitro study in zebrafish [26].

DAZL protein binds with thousands of mRNA transcripts at the 3’UTR, as reported in different species. In undifferentiated mouse spermatogonia, DAZL binds with about 2500 transcripts at a UGUU(U/A) motif and increases the translation of key spermatogonial gene regulatory factors [17]. In the mouse testis, DAZL binds with about 3000 transcripts, particularly at a UGUU motif, and increases the translation of binding mRNAs associated with the spermatogenesis [16]. Another study using the mouse testis revealed that the DAZL binds with over 3900 transcripts at the GUU-containing regions. Particularly the GUUG motif is present in a significant proportion of the transcripts [13]. When analyzing the gene ontology of about 500 genes that interacted with *DAZL* and reduced mRNA levels in *DAZL* KO condition, most gene ontology terms are associated with various functions critical for spermatogenesis [13]. In an in vitro study, zebrafish DAZL binds with its target transcripts at a GUUC motif and promotes translation [26]. In a study using mouse oocytes, 1799 transcripts that contained a DAZL binding motif [UUU(C/G)UUU] are revealed [15]. According to yeast trihybrid assay, oligo(U) stretches interspersed by G showed stronger interactions with DAZL than interspersed by C, A, or U [27]. In addition, DAZL binding at a UUUGUUUU motif is reported in the mouse PGCs [14]. The authors demonstrated that DAZL acts as a translational repressor of core pluripotency-, somatic differentiation-, and apoptosis-related genes in nascent PGCs [14].

### 2.2. Gene Ontology Enrichment of DAZL and Its Interacting Genes

**Figure 1:**
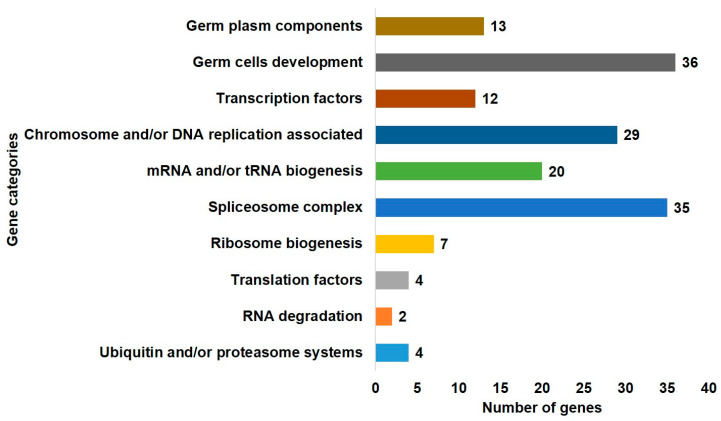
The gene ontology enrichment of deleted in azoospermia like (*DAZL*) interacting genes. *DAZL* and a set of *DAZL* interacting genes predicted in chickens using the search tool for the retrieval of interacting genes/proteins (STRING) database and motifs analysis are subjected to the AmiGO 2 gene ontology database and Kyoto Encyclopedia of Genes and Genomes (KEGG) database. Genes in the germ plasm component and germ cells development categories are identified using the AmiGO database. Genes in all other categories (processes related to zygotic genome activation (ZGA)/maternal-to-zygotic transition (MZT)) are identified using the KEGG database.

In this study, the predicted *DAZL* interacting genes and the *DAZL* gene, are subjected to AmiGO 2 [28] and Kyoto Encyclopedia of Genes and Genomes (KEGG) [29] databases for gene ontology enrichment analysis. In the AmiGO 2 database, we searched the genes involved in the germ plasm and germ cells development categories. In the KEGG database, we searched the genes involved in processes related to ZGA/MZT. Genes in each category are retrieved primarily based on the references in chicken. Due to less information in chicken, genes are retrieved secondarily based on the references in other species, i.e., zebrafish, fruit fly, and roundworm for the germ plasm category and human, mouse, zebrafish, fruit fly, and roundworm for all other categories. In the gene ontology enrichment using AmiGO, we identified 13 genes in the germ plasm component category and 36 genes in the germ cells development category. In the gene ontology enrichment using KEGG, we identified 12 genes in the transcription factors category, 29 genes in the chromosome and/or DNA replication associated category, 20 genes in the mRNA and/or tRNA biogenesis category, 35 genes in the spliceosome complex category, 7 genes in the ribosome biogenesis category, 4 genes in the translation factors category, 2 genes in the RNA degradation category, and 4 genes in the ubiquitin and/or proteasome systems category (Figure 1). Some of the genes are overlapped in the gene ontology terms; however, we have not regulated the overlapped genes. Because excluding the overlapping genes could mislead that they are not involved in other functions. For instance, the *DAZL* is identified in both germ plasm and germ cells development categories.

In the oocytes of chickens, specific maternally produced RNAs, proteins, and energy-rich mitochondria are stored in a specialized structure called germ plasm and inherited into the cells specifying precursor germ cells during intrauterine embryonic development. After fertilization, the chicken zygote undergoes asymmetric cleavage to yield preblastodermal cells. The first two divisions occur synchronously to produce four polarized preblastodermal cells, and then asynchronous cleavage continues in a radial manner [30]. According to an earlier study, the *DAZL* (a germ plasm component) is detected in the chicken oocyte and zygote. During the intrauterine embryonic development, *DAZL* is localized in the cleavage furrows at EGK.I and in several central cells at EGK.III to EGK.X [18]. This result revealed a possible role of *DAZL* in germ cells specification and development during the chicken intrauterine embryonic development, which also has other important features such as ZGA and MZT. During ZGA, the transcriptions of zygotic genes are activated by the maternal RNAs and proteins present in the zygote’s cytoplasm. During MZT, maternal RNAs and proteins are cleared, and further embryonic development processes are completely controlled by the zygotic RNAs and proteins [21].

### 2.3. Detection of DAZL and Its Interacting Genes in the Chicken Intrauterine Embryos

After gene ontology classification, genes in each category are separately subjected to the STRING database to show their interaction with the *DAZL* gene. In addition, the transcriptomic expression of genes in each category is detected in the chicken intrauterine embryos using our previously generated WTS data (NCBI GEO accession number GSE86592). In our previous study, the WTS data are generated using the bulked chicken intrauterine samples, including oocyte, zygote, and EGK stage embryos (EGK.I, EGK.III, EGK.VI, EGK.VIII, and EGK.X). The authors analyzed the data based on the galGal4 and galGal5 versions of the chicken reference genome [31]. The current version of the chicken reference genome is galGal6a. Therefore, we preprocessed the raw-sequencing data to generate clean reads using Trimmomatic v. 0.39 [32] and mapped the clean reads into the galGal6a reference genome in this study. Then, HTSeq-count [33] is used to quantify the gene expression levels (number of mapped reads) with the RefSeq genomic gene transfer format (GTF) of galGal6a, and finally, 24,154 genes are annotated. The read counts are normalized with the trimmed mean of M-values (TMM) normalization method. Then, the log2 (TMM) is used for the better visualization of the gene expression through heatmap and line graph. The mapped clean-read counts and the log2 TMM-normalized values of *DAZL* and *DAZL* interacting genes in the chicken oocyte, zygote, and intrauterine embryos are shown in Tables S2 and S3, respectively.

#### 2.3.1. Germ Plasm and Germ Cells Development Categories

**Figure 2:**
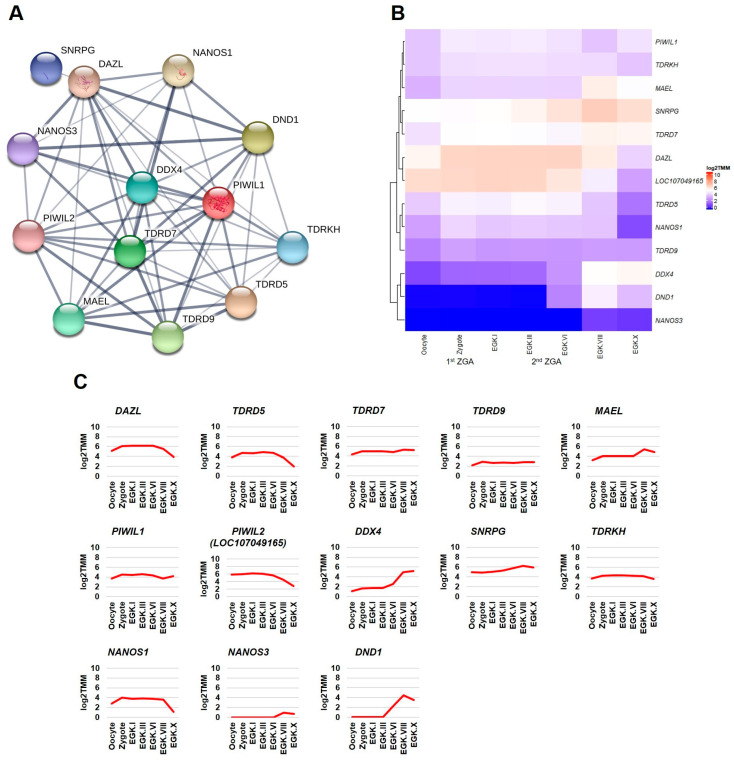
Interaction network and intrauterine embryonic expression of *DAZL* interacting genes identified in the germ plasm category. The confidence-based direct interaction of *DAZL* with genes identified in the germ plasm category is prepared using the STRING database (**A**). The expression patterns of *DAZL* interacting genes (germ plasm category) in the chicken oocyte, zygote, and Eyal-Giladi and Kochav (EGK) stage intrauterine embryos (EGK.I to EGK.X) are examined using the whole-transcriptome sequencing (WTS) data. log2 trimmed mean of M-values (TMM)-normalization is used to better visualize the gene expression through heatmap (**B**) and line graph (**C**).

This section first examined the transcriptome-based expression patterns of *DAZL* and its interacting genes of germ plasm category (Figure 2A) in the chicken intrauterine embryos. According to the transcriptome-based expression patterns analysis, *DAZL* transcripts are increased in the zygote, maintained at the increased level until EGK.VI, and decreased at EGK.VIII and EGK.X. The transcripts of *TDRD5*, *TDRD7*, *TDRD9*, *MAEL*, *PIWIL1*, *PIWIL2*, *DDX4*, *SNRPG*, *TDRKH*, and *NANOS1* genes are also increased in the zygote. However, they are decreased, maintained, or increased after EGK.VI. *NANOS3* transcripts are slightly increased from EGK.VIII. *DND1* transcripts are sharply increased at EGK.VI and EGK.VIII and slightly decreased at EGK.X (Figure 2B,C). In chickens, the 1st wave of ZGA (results in a small number of zygotic transcripts) occurs in the zygote, and the 2nd wave of ZGA (results in a large number of zygotic transcripts) occurs at EGK.III-VI, just before the MZT time [21,22,23]. The maternal RNAs and proteins are degraded just before MZT, which could be the reason for the decrease in *DAZL*, *TDRD5*, and *PIWIL2*. The 2nd wave of ZGA could play a role for the genes showing a similar level or increased level after EGK.VI. In addition, the expression of *DAZL* is strong in the oocyte and intrauterine embryos right from the zygote. Therefore, it could enhance the translation of *TDRD5*, *TDRD7*, *TDRD9*, *MAEL*, *PIWIL1*, *PIWIL2*, *DDX4*, *SNRPG*, *TDRKH*, and *NANOS1*, for functional cooperation during germ cells specification and development. In contrast, *DAZL* could enhance the translation of *NANOS3* and *DND1* for functional cooperation at a later stage of germ cells development.

**Figure 3:**
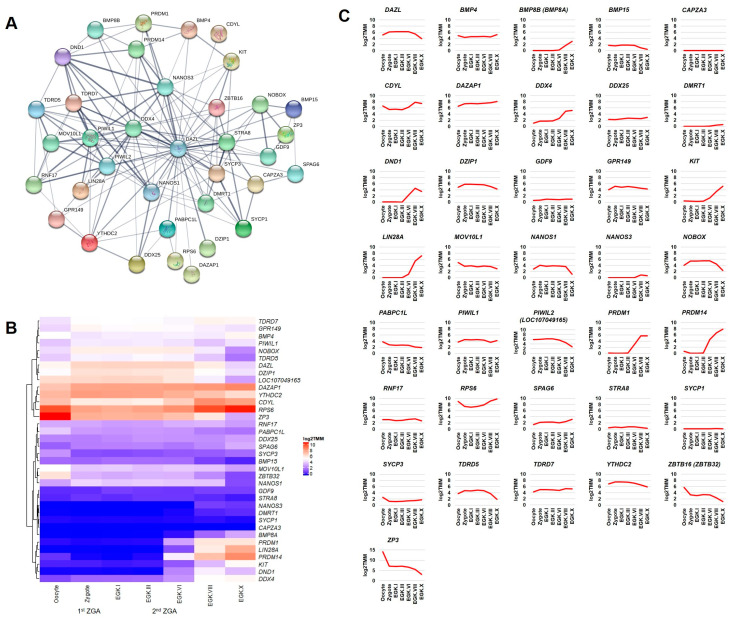
Interaction network and intrauterine embryonic expression of *DAZL* interacting genes identified in the germ cells development category. The confidence-based direct interaction of *DAZL* with genes identified in the germ cells development category is prepared using the STRING database (**A**). The expression patterns of *DAZL* interacting genes (germ cells development category) in the chicken oocyte, zygote, and EGK stage intrauterine embryos (EGK.I to EGK.X) are examined using the WTS data. log2 TMM-normalization is used to better visualize the gene expression through heatmap (**B**) and line graph (**C**).

Next, we examined the transcriptome-based expression patterns of *DAZL* and its interacting genes of germ cells development category (Figure 3A) in the chicken intrauterine embryos. At least nine genes (*DAZL*, *TDRD5*, *TDRD7*, *PIWIL1*, *PIWIL2*, *DDX4*, *NANOS1*, *NANOS3*, and *DND1*) identified in the germ plasm category are also identified in the germ cells development category using the AmiGO database. Although the germ plasm components are critical for early germ cells development, the remaining four genes (*TDRD9*, *MAEL*, *SNRPG*, and *TDRKH*) are not identified in the latter category, indicating the limitation of sources from computational databases. Apart from the above-described genes, the transcripts of *BMP4*, *BMP15*, *CDYL*, *DAZAP1*, *DDX25*, *DZIP1*, *GDF9*, *GPR149*, *NOBOX*, *RNF17*, *SPAG6*, *STRA8*, and *YTHDC2* genes are increased in the zygote (during 1st ZGA); however, they are decreased, maintained, or increased after EGK.VI. The transcripts of *BMP8A*, *KIT*, *LIN28A*, *PRDM1*, *PRDM14*, *RPS6*, and *SYCP3* genes are increased at EGK.III-VI (2nd ZGA). The transcripts of the *DMRT1* gene are slightly increased from EGK.VIII. The transcripts of *MOV10L1*, *PABPC1L*, *ZBTB16*, and *ZP3* genes are continuously decreased during the intrauterine embryonic development. Only a few transcripts of the *SYCP1* gene and no transcripts of the *CAPZA3* gene are detected during the intrauterine embryonic development (Figure 3B,C).

The expression pattern of most genes described above are not reported in earlier studies during the chicken intrauterine embryonic development, and therefore, our study could be the first. We suggest that the genes transcriptionally active in the zygote (1st ZGA) could contribute to the germ cells specification and development than that of genes transcriptionally active at EGK.III-VI (2nd ZGA) or EGK.VIII-X (post-ZGA). To support our suggestion, for instance, the expression of *DDX4* (a germ plasm component) is detected continuously in the chicken oocytes and EGK.I-X stage embryos and reported to play an important role in the germ cells specification and development [34]. The expression of *PRDM1* (not a germ plasm component) is detected at EGK.V-X stages and also reported to play an important role in the chicken germ cells specification and development [35]. Moreover, during mouse development, *PRDM1* and *PRDM14* (key genes for germ cells specification in the induction mode) are activated in a few posterior epiblast cells by BMP signals from the extra-embryonic ectoderm. The *PRDM1* and *PRDM14* positive cells give rise to PGCs through the repression of somatic genes, activation of germness genes, reacquisition of pluripotency, and genome-wide epigenetic reprogramming [3].

#### 2.3.2. Transcription Factors Category

**Figure 4:**
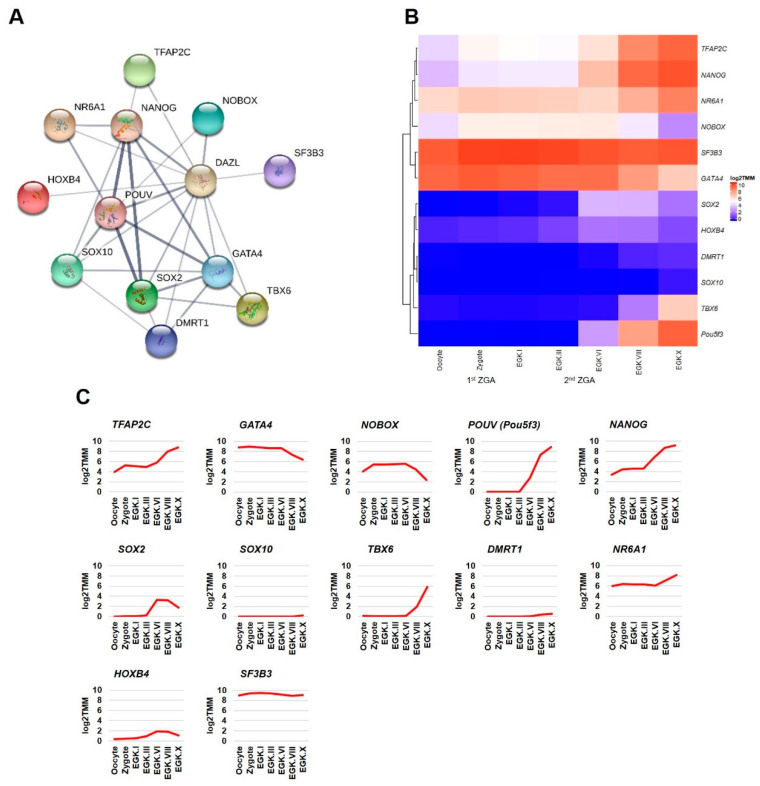
Interaction network and intrauterine embryonic expression of *DAZL* interacting genes identified in the transcription factors category. The confidence-based direct interaction of *DAZL* with genes identified in the transcription factors category is prepared using the STRING database (**A**). The expression patterns of *DAZL* interacting genes (transcription factors category) in the chicken oocyte, zygote, and EGK stage intrauterine embryos (EGK.I to EGK.X) are examined using the WTS data. log2 TMM-normalization is used to better visualize the gene expression through heatmap (**B**) and line graph (**C**).

This section examined the transcriptome-based expression patterns of *DAZL* interacting genes of transcription factors category (Figure 4A) in the chicken intrauterine embryos. Among the *DAZL* interacting transcription factors, *NOBOX* and *DMRT1* are identified and described above in the germ cells development category. In the transcription factors category, *TFAP2C*, *NANOG*, *NR6A1*, and *SF3B3* genes are slightly increased in the zygote (during 1st ZGA) and largely increased from EGK.III-VI (during 2nd ZGA). Moreover, the expression of *SF3B3* is high at all stages compared to other genes in this category. *POUV*, *SOX2*, *TBX6*, and *HOXB4* genes are increased only from EGK.III-VI (during 2nd ZGA). Although detected in the zygote, *GATA4* shows a decreasing pattern as similar to *NOBOX*. Only a few transcripts of *SOX10* are detected at EGK.X as similar to *DMRT1* (Figure 4B,C). After fertilization, the zygotic transcription during 1st ZGA and 2nd ZGA depends largely on maternal transcription factors stored in the cytoplasm of oocytes. Several transcription factors, such as *NANOG*, *SOX2*, *POU5F1*, *POU5F3*, and *DUX*, are reported as key genes for initiating the transcription of large numbers of zygotic genes in different species [21,36]. Among the *DAZL* interacting transcription factors shown in this study, the expression patterns of three pluripotency-related transcription factors, such as *NANOG*, *POUV*, and *SOX2*, are reported in a previous study during the intrauterine embryonic development in chickens [37]. The expression patterns of *NANOG* and *POUV* showed in the present study are similar to the previous study. In contrast, the expression of *SOX2* is not detected in chicken oocytes or during intrauterine development in the previous study, indicating the sensitivity of different techniques.

#### 2.3.3. Other Categories of Processes Related to ZGA/MZT

This section examined the intrauterine embryonic expression patterns of *DAZL* interacting genes identified in the chromosome and/or DNA replication associated category (Figure S2A–C), mRNA and/or tRNA biogenesis category (Figure S2D–F), and spliceosome complex category (Figure S2G–I). In addition, this section examined the intrauterine embryonic expression patterns of *DAZL* interacting genes identified in the ribosome biogenesis category (Figure S3A–C), translation factors category (Figure S3D–F), RNA degradation category (Figure S3G–I), and ubiquitin and/or proteasome systems category (Figure S3J–L). These categorized processes are crucial for the ZGA/MZT in developing embryos. In the classic nucleocytoplasmic (N/C) ratio model of ZGA, an increasing quantity of nuclear material relative to a constant cytoplasmic volume, by progressive cell divisions, alleviates transcriptional repression [38]. In addition, during the pronuclear stage, dynamic changes in the chromatin state and histone modifications drive transcriptional activation in parental pronuclei [39]. The newly produced precursor mRNA transcripts are converted into mature mRNA transcripts by RNA splicing, in which introns are removed, and exons are joined together. Spliceosome, a large ribonucleoprotein complex, catalyzes the RNA splicing [40]. The maternal RNAs and proteins are cleared before MZT by maternal mode and a zygotic mode. In the maternal mode, RNAs are cleared by maternally provided factors, such as RNA-binding proteins. The proteins are cleared by three mechanisms, including the autophagy-lysosome pathway, ubiquitin-proteasome pathway, and endocytosis. The zygotic mode mostly depends on newly produced zygotic transcripts (including microRNAs) and proteins [21,38,41]. Figure S4 shows the direct interaction (with *DAZL*) and intrauterine embryonic expression patterns of *DAZL* interacting genes that are not identified in the gene ontology enrichment terms discussed in this study. Overall, it is suggested that the genes strongly expressed from zygote to EGK.X could play a predominant role in these categories for the mentioned functions.

#### 2.3.4. Differential Expression of *DAZL* and Its Interacting Genes between Consecutive Stages

**Figure 5:**
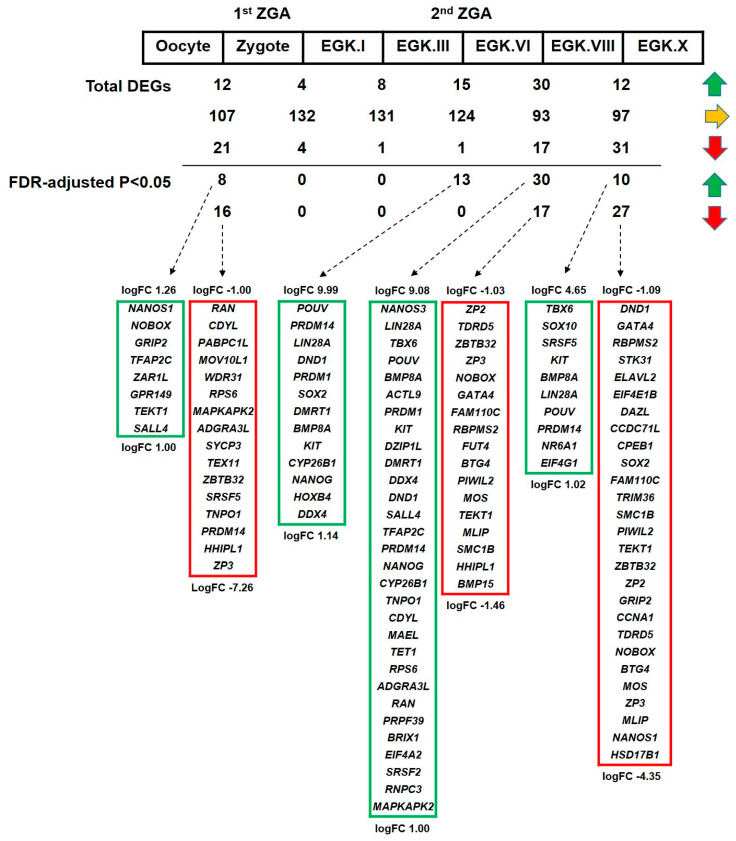
Differentially expressed *DAZL* interacting genes between consecutive stages of intrauterine embryonic development in chicken. The differentially expressed genes between stages are detected using a negative binomial-based GLM. In total, six statistical tests are performed: oocyte versus zygote, zygote versus EGK.I, EGK.I versus EGK.III, EGK.III versus EGK.VI, EGK.VI versus EGK.VIII, and EGK.VIII versus EGK.X. FDR-adjusted *p* < 0.05 is considered for significant genes. logFC > 1 is considered for upregulated genes (green arrow), logFC < −1 is considered for downregulated genes (red arrow), and genes fall between these logFC conditions is considered as unchanged (yellow arrow). The logFCs of genes listed at the top and bottom in a box are mentioned.

The differential expression of *DAZL* and its interacting genes (upregulation or downregulation) between consecutive stages of intrauterine embryonic development in chicken is detected using a negative binomial-based generalized linear model (GLM). We performed six statistical tests, such as oocyte versus zygote, zygote versus EGK.I, EGK.I versus EGK.III, EGK.III versus EGK.VI, EGK.VI versus EGK.VIII, and EGK.VIII versus EGK.X. The false discovery rate (FDR)-adjusted *p* < 0.05 is considered for identifying the significant genes. The logFC (fold change) >1 is considered for identifying the upregulated genes and logFC < −1 is considered for identifying the downregulated genes. Among the total genes analyzed, only a few genes are upregulated or downregulated. In contrast, over 100 genes are unchanged from the 1st ZGA time (oocyte versus zygote) to the 2nd ZGA time (EGK.III versus EGK.VI). When we applied FDR (*p* < 0.05), significantly upregulated or downregulated genes are not detected at zygote versus EGK.I and EGK.I versus EGK.III conditions (Figure 5 and Table S4). This result indicates that most of the *DAZL* interacting genes undergo transcriptional silencing during the period between 1st ZGA and 2nd ZGA in chickens. Differing from mouse, the human, frog, zebrafish, fruit fly, and roundworm embryos undergo several cell cycles between the 1st ZGA and 2nd ZGA. It is reported in human embryos that the genes undergo transcriptional silencing during the period between 1st ZGA and 2nd ZGA [42,43].

#### 2.3.5. Exon–Intron Specific RT-qPCR Validation of *DAZL* and Candidate Interacting Genes

**Figure 6:**
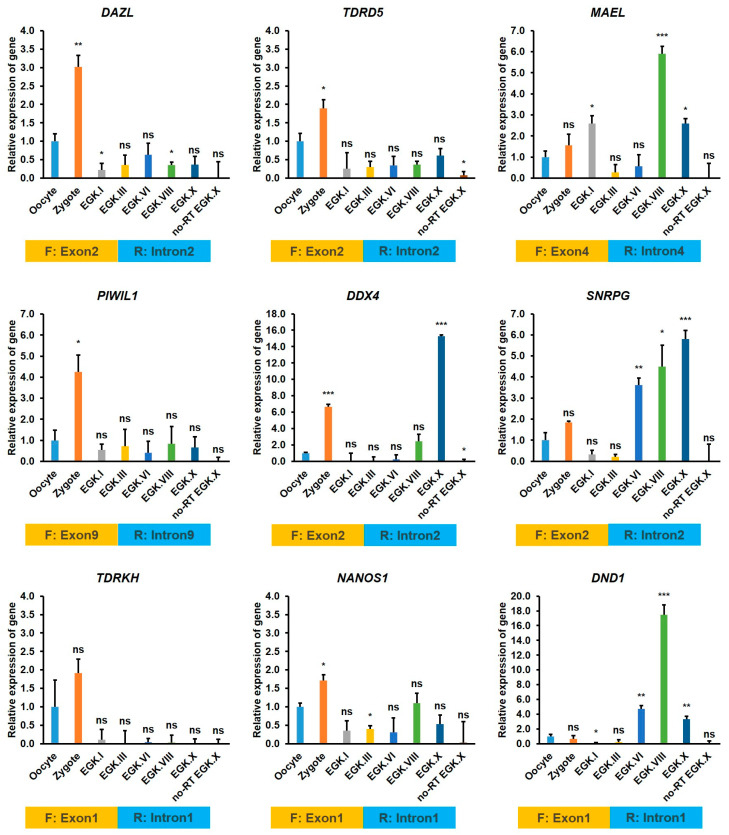
Exon–intron specific RT-qPCR validation of *DAZL* interacting genes during the intrauterine embryonic development in chicken. Total RNAs from the oocyte, zygote, and EGK.I to EGK.X stage embryos are reverse transcribed using the random hexamer. No reverse transcriptase (no-RT) negative control of EGK.X embryos are also prepared to monitor the genomic DNA contamination. Then, the cDNAs are amplified using the gene-specific forward and reverse primers. The forward primer (F) on a suitable exon region and the reverse primer (R) on the following intron region is designed with the GenBank sequences of each gene. qPCR is performed in triplicate with three independent samples. The relative quantification of the gene expression is normalized with the chicken *GAPDH* and a reference sample (oocyte) and analyzed by the 2^−ΔΔCt^ method. Significant differences between the oocyte and other test samples are determined using the Student’s *t*-test. Statistical significance is ranked as * *p* < 0.05, ** *p* < 0.01, or *** *p* < 0.001. ns—not significant.

Finally, we performed exon–intron specific RT-qPCR to further validate the newly produced zygotic transcripts (precursor mRNAs) of *DAZL* and its candidate interacting genes identified in the germ plasm category. We designed a forward primer on the exon region and a reverse primer on the following intron region with the GenBank sequences of each gene retrieved from the NCBI database. The preovulatory large F1 oocyte, zygote, and EGK stage intrauterine embryos, such as EGK.I, EGK.III, EGK.VI, EGK.VIII, and EGK.X are collected from the chickens as described early [22,31]. Total RNAs from these samples are reverse transcribed using the random hexamer, and the cDNAs are amplified using the gene-specific primers (Table S5). As shown in Figure 6, precursor mRNAs of *DAZL*, *TDRD5*, *PIWIL1*, *TDRKH*, and *NANOS1* are detected at a higher level in the zygote and a lower level in other stages of development (EGK.I to EGK.X), indicating that these genes are transcriptionally active during 1st ZGA. The precursor mRNAs of *MAEL* and *DDX4* are also detected at a higher level in the zygote (during 1st ZGA); however, they are detected at a more high level after 2nd ZGA (at EGK.VIII for *MAEL* and at EGK.X for *DDX4*). The precursor mRNAs of *SNRPG* is detected at a higher level in the zygote; however, the transcripts are detected at a more highly increasing pattern from EGK.VI to EGK.X. The precursor mRNAs of *DND1* is detected high at 2nd ZGA and peaks at EGK.VIII. These results reinforce that the germ plasm components are transcribed early during the intrauterine embryonic development and could play a crucial role in the germ cells specification and development in chickens.

## 3. Materials and Methods

### 3.1. Prediction of DAZL Interacting Genes and Motifs Analysis

The *DAZL* interacting genes in chickens (*Gallus gallus*) are predicted using the STRING database (v. 11.0) [25]. *DAZL* interacting genes are analyzed under medium to highest confidence levels (score: 0.4–1.0) using active interaction sources such as text mining, experiments, databases, co-expression, neighborhood, gene fusion, and co-occurrence. The number of interactors set to “no more than 500” for the first shell and “none” for the second shell. The retrieved gene symbols are updated according to the latest genome versions of chickens (galGal6a) in the NCBI database [44], and also the corresponding chromosome numbers, gene IDs, and RefSeq mRNA IDs are collected.

The 3’ UTR sequences of *DAZL* interacting genes are extracted based on the coordinates of the stop codon and the end position of each transcript defined by the galGal6a reference genome (RefSeq assembly accession: GCF_000002315.6) and the corresponding generic feature format (GFF) gene annotation available under the BioProject PRJNA13342. The previously reported DAZL protein binding motifs, such as UGUU(U/A) [17], UGUU [16], GUU(U/A) [17], GUUC [26], GUUG [13], and UUU(C/G)UUU [15,27], are investigated by screening the 5’ to 3’ direction of the 3’ UTR sequences of each transcript on + or − strand via an in-house Python script (Python 3.7.3). Using a sliding window sequence of 4, 5, and 7 bp in length from the 3’ UTR, the occurrence of a perfect match to the above binding motifs is counted. Motif counts on the 3’ UTR of the primary transcript variant are considered if a gene consists of multiple transcript variants. The python script and other raw files used to analyze the DAZL binding motif are given in the supplementary material (File name: Python script and raw files).

### 3.2. Gene Ontology Enrichment, and Sub-Interaction Analysis of Predicted Genes with DAZL

The predicted *DAZL* interacting genes, along with *DAZL*, are subjected to the AmiGO 2 database [28] and their involvement in the germ plasm and germ cells development categories using the Protein ANalysis THrough Evolutionary Relationships (PANTHER) overrepresentation test (Fisher’s exact and Bonferroni correction) are analyzed. Next, the gene lists are subjected to the KEGG database [29] and their involvement in processes related to ZGA/MZT are analyzed. Genes are arranged into the categories primarily based on the references in chicken and secondarily based on the references in human, mouse, zebrafish, fruit fly, and roundworm. Next, the gene lists of each category are separately subjected to the STRING database to analyze their interaction with *DAZL*. The number of interactors is set to “query genes only” for the first shell and “none” for the second shell, and finally, the confidence-based networks of each category are retrieved.

### 3.3. Preprocessing of WTS Data from the Chicken Intrauterine Embryos

The WTS data generated using the bulked chicken intrauterine samples, including oocyte, zygote, and EGK stage embryos (EGK.I, EGK.III, EGK.VI, EGK.VIII, and EGK.X) in our earlier study [31] are employed and analyzed differently in this study. A total of 21 raw-sequencing data (including biological triplicates) from the bulked chicken intrauterine samples are publicly available in the NCBI GEO database (GSE86592). From the Illumina NextSeq 500 platform produced raw paired-end reads (150 bp), adapter sequences and poor-quality reads are removed, and the clean reads (Table 1) are generated using Trimmomatic v.0.39 [32]. The quality of the clean reads, including minimum read length > 75 and Phred score > 30, is checked using FastQC v.0.11.9 (bioinformatics.babraham.ac.uk/projects/fastqc).

### 3.4. Alignment and Quantification of Mapped Reads

The clean reads are mapped (Table 1) into the galGal6a reference genome (GCF_000002315.6). HISAT2 v.2.2.0 is used for the alignment [45]. Following the alignment, the sequence alignment/map files (.SAM files) are converted into binary alignment/map files (.BAM files) using SAMtools v.1.10 [46]. Then, we used HTSeq-count [33] to quantify the gene expression levels (number of mapped reads) with the RefSeq genomic GTF of galGal6a (GCF_000002315.6). The read counts are normalized with the TMM normalization method, and the dispersion parameter of each sample is estimated before the statistical tests. log2(TMM) is used for the visualization of the gene expression through heatmaps and line graphs.

Then, differentially expressed genes between consecutive stages of intrauterine embryonic development in the chicken are detected using a negative binomial-based generalized linear model (GLM) as follows:

(1) $$log\left(E\left(Expression_{i}\right)\right)=\mu +Stage_{i},$$

where the *Expression* represents normalized counts and the *Stage* represents multiple groups containing seven developmental stages across the 21 WTS samples. In total, six statistical tests, oocyte versus zygote, zygote versus EGK.I, EGK.I versus EGK.III, EGK.III versus EGK.VI, EGK.VI versus EGK.VIII, and EGK.VIII versus EGK.X, are performed using the edgeR package implemented in R [47]. Likelihood ratio tests are performed to obtain the *p*-values, and the FDR is used for multiple testing correction. FDR-adjusted *p* < 0.05 is considered for identifying significant genes, while logFC > 1 is considered for identifying upregulated genes, and logFC < −1 is considered for identifying downregulated genes. The WTS data of the *DAZL* gene and *DAZL* interacting genes are screened and presented in this study.

### 3.5. Experimental Animals and Animal Care

The care and experimental use of White Leghorn (WL) chickens are approved by the Institute of Laboratory Animal Resources, Seoul National University (SNU-190401-1-1). Chickens are maintained according to a standard management program at the University Animal Farm, Seoul National University, Korea. The procedures for animal management, reproduction, and embryo manipulation adhered to the standard operating protocols of our laboratory.

### 3.6. Sample Collection and Exon–Intron Specific RT-qPCR Validation of DAZL and Its Interacting Genes

The preovulatory large F1 oocyte, zygote, and EGK stage embryos (EGK.I, EGK.III, EGK.VI, EGK.VIII, and EGK.X) are collected in triplicate: 5 oocytes for each replication; 3 zygotes for each replication; 6, 5, and 6 embryos at EGK.I for each replication; 6 embryos at EGK.III for each replication; 6, 6, and 7 embryos at EGK.VI for each replication; 5, 5, and 3 embryos at EGK.VIII for each replication; and 10 embryos at EGK.X for each replication. The samples are collected from the WL hens as described in our earlier studies [22,23,31]. Total RNAs are extracted from each replication of the samples using TRIzol reagent (Invitrogen, Carlsbad, CA, USA). The quality and quantity of the total RNAs are examined using the Trinean DropSense96 system (Trinean, Gentbrugge, Belgium), a RiboGreen Kit (Invitrogen), and an Agilent 2100 Bioanalyzer (Agilent Technologies, Santo Clara, CA, USA) [22,23,31]. Then, 1 μg of total RNAs are used for the random hexamers primed cDNA synthesis using the SuperScript III First-Strand Synthesis System (Invitrogen). No reverse transcriptase (no-RT) negative control of EGK.X embryos are also prepared in triplicate. The cDNAs are serially diluted 5-fold and equalized quantitatively for the qPCR amplification using a StepOnePlus real-time PCR system (Applied Biosystems, Foster City, CA, USA). The qPCR reaction mixture contained 2 μL of PCR buffer, 0.4 μL of 10 mM dNTP mixture, 10 pmol each of gene-specific forward and reverse primers, 1 μL of 20× EvaGreen (Biotium, Hayward, CA, USA), 0.2 μL of Taq DNA polymerase, and 2 μL of cDNA to a final volume of 20 μL. The qPCR thermal condition contained an initial incubation at 95 °C for 5 min, followed by 40 cycles at 95 °C for 30 s, 60 °C for 30 s, and 72 °C for 30 s. The reaction is ended after final incubation at the system’s dissociation temperature. The forward and reverse primers covering the exon–intron region of each target gene (Table S5) are designed using the Geneious Prime software (Biomatters, Ltd., Auckland, New Zealand) with the GenBank sequences retrieved from the NCBI database. For chicken *GAPDH*, primers are designed using the RefSeq mRNA sequences (NM_204305), containing exons only. qPCR is performed in triplicate with three independent samples. The relative quantification of the gene expression is normalized with the chicken *GAPDH* and a reference sample (oocyte), and analyzed by the 2^−ΔΔCt^ method. Significant differences between the oocyte and other test samples are determined by Student’s *t*-test using the GraphPad Prism software (San Diego, CA, USA). Statistical significance is ranked as * *p* < 0.05, ** *p* < 0.01, or *** *p* < 0.001.

## 4. Conclusions

In this study, we identified a set of *DAZL* interacting genes in chickens using in silico prediction method. Then, we analyzed the WTS-based expression patterns of *DAZL* and its interacting genes in the chicken oocyte, zygote, and EGK stage intrauterine embryos (EGK.I to EGK.X). Collectively, the *DAZL*, and most of its interacting gene transcripts, are increased either at 1st ZGA or 2nd ZGA, indicating their involvement in germ cells specification and development during chicken intrauterine embryonic development.

## Figures and Tables

**Table 1:** Summary statistics of the RNA-seq preprocessing.

Samples	Raw Reads	QC Passed Reads	QC Passed Ratio	Mapped Reads	Mapped Ratio	Uniquely Mapped Reads	Uniquely Mapped Ratio
Oocyte_S1	118,184,502	112,049,150	94.81%	100,138,495	89.37%	88,202,016	88.08%
Oocyte_S2	119,066,268	112,087,560	94.14%	100,319,697	89.50%	84,762,286	84.49%
Oocyte_S3	124,554,030	118,997,350	95.54%	105,789,456	88.90%	92,455,076	87.40%
Zygote_S4	110,348,652	106,756,296	96.74%	94,855,747	88.85%	84,724,358	89.32%
Zygote_S5	111,604,036	107,999,168	96.77%	96,008,462	88.90%	84,931,128	88.46%
Zygote_S6	102,075,504	100,055,858	98.02%	87,757,716	87.71%	78,370,790	89.30%
EGK.I_S1	116,899,114	113,818,628	97.36%	94,434,196	82.97%	80,459,294	85.20%
EGK.I_S2	125,479,340	122,894,028	97.94%	100,660,269	81.91%	85,257,994	84.70%
EGK.I_S4	103,691,686	100,377,694	96.80%	88,604,778	88.27%	77,917,230	87.94%
EGK.III_S3	125,126,674	121,753,362	97.30%	102,957,681	84.56%	87,289,704	84.78%
EGK.III_S4	115,130,736	112,715,380	97.90%	94,110,789	83.49%	80,524,216	85.56%
EGK.III_S5	93,275,856	91,430,970	98.02%	76,574,666	83.75%	65,587,730	85.65%
EGK.VI_S1	127,298,262	124,150,076	97.53%	100,420,929	80.89%	84,066,832	83.71%
EGK.VI_S5	133,416,206	130,446,328	97.77%	115,208,886	88.32%	100,992,302	87.66%
EGK.VI_S6	101,065,710	99,208,584	98.16%	83,024,395	83.69%	70,955,576	85.46%
EGK.VIII_S2	138,471,542	134,802,776	97.35%	107,760,966	79.94%	90,024,204	83.54%
EGK.VIII_S3	116,500,772	112,792,536	96.82%	87,374,002	77.46%	71,465,540	81.79%
EGK.VIII_S4	146,364,730	142,618,126	97.44%	114,447,799	80.25%	96,284,262	84.13%
EGK.X_S5	141,549,042	135,461,004	95.70%	110,279,481	81.41%	92,973,012	84.31%
EGK.X_S6	155,994,076	148,219,000	95.02%	118,622,560	80.03%	99,114,152	83.55%
EGK.X_S7	133,595,886	126,451,838	94.65%	101,943,745	80.62%	85,789,668	84.15%
Average	121,890,125	117,861,224	96.75%	99,109,272	84.32%	84,864,160	85.68%

QC passed paired reads are obtained from the Trimmomatic and FastQC. Mapped and uniquely mapped reads are obtained from the HISAT2.

## Supplementary Materials

Supplementary materials can be found at https://www.mdpi.com/1422-0067/21/21/8170/s1.
