# Supplementary material for: Whole-Transcriptome Sequencing-Based Analysis of DAZL and Its Interacting Genes during Germ Cells Specification and Zygotic Genome Activation in Chickens
Source: Int J Mol Sci. 2020 Oct 31;21(21):8170. doi: 10.3390/ijms21218170 (PMC7672628; doi:10.3390/ijms21218170)
Supplement: Supplementary file 1 [file ijms-21-08170-s001.zip › Figure S1_Final.pdf]

>*DAZL*, NM_204218
UGAACAAAGACUUUGAAGUACAUAAAUGUAUUACUUUGAUGUUCCUACAGUUCAGUUUAGUAAGAUGUGUAGUAAAAAGUGUAACCUUGUUCAAAAA
GUUGCUUCAAGUUGAUGUUUGUGUUCUGUUUUACCUGUUCCAGAAUAGCUAUUUUUGCUUGAGAAGUUUGAAGUUGUAAGAGUUGAAAUAUUUCCAG
GUUUUAUUACUAGCUUGCAUGCUUUUCCUGCUAACUAACUGAAAUGCUAAUCUUAAGGAAUUUAUAUGGGGAAGGGGAAAAAGAAAAACACUUUGU
UUGGUAUGUGUGGAUUUUCUUCUGAGCUUUAAGGUACAGUUUGUUGCAUGUUAAAAUUUAGUUCUUAUUAAACCACAACUUUAAGUUACUAACGUCA
ACCAGUUACCUCUUGCAGUUCAAAAGUUGAAGCAGUUCCUUGUCCAAGAUGGAGUAUUUUAAAACUGAGCUCUUAAUCAGUGGAACAGAAGACGUCA
CGGUGUAACUCAACUGAAGCCCUUUAAGUCCCGGUUCUCUUUAGACUACCUAAUCAAUGUCUUUGUUUGCUAACGACAGUUCAUCUAUGUGAAUCCU
AAAAUUCCUAUAUGUAACUUAAGAUGCAAGAAUGUAAUUAGUUACAUUGGCUGCUCAGUGGAGUAUGACUUUUUUUUUACUGGAUUAAUUUUAGCA
AUACCUGUAUCUUAAAAUUGUGAGAAAAUACUGCAUUUAAAAUAUGCCUAACUUUGUGACGCAAUAUGUUAAUCAAAGAAUACAUGUAAGCAUAUUU
UAAAAAUAAUUAUGUAGAUUUUAGUCAUGUAUUUUGAAACAAUUAAAAUUUUUAAUUUUGACU

>*DND1*, XM_015293536
GGGUGCAUGUGGGCACACGUUGUGCCCCAGCUAUGAAAUGAACCCGAGCCACCUGAUGUCACGUCGUGGAGCAGAGCUGAGCCCAGCGGGGCCGCGU
UCCUGUUCAGCCUUGUUUUGAGUUCGUUGGGGUUUGUUUGAAGGCCAUCUGGAGGAUGCGGGCGCUGCCCCAUCCCUAUCAGCACUGGUUCUGUUGU
UGGCAGCAGGCGUGCCCUGCCUGCUUCCAUGUUCUCCUGUUGAUGAAGUUGUUUUGUGUUGCUGCGGUGCUGCAGGGCUCUCAGGAGGAGCAGCAGC
UGCCUGGGAAGGAUGCGUUCUGGGGGCGCCGGGCCCCAUUGCCUGCAGCCCCAUUGUCGCCGUGGGGCAGCCAGGGAGCCCUGAGCUGUCUGCAGGC
ACUGCCCCCAGCCUGGAGGCCAGAGUCCACCUGCUGGCUAUUAAAGGAGCCUGGGAAGCACCGGUGGUGUUCUGUUGCGUUUUGUUGCUGCGGUUUC
CCUGGGUGGGUCCCGUUGUGCAGCCAGGCCCACAUUGGGCAGCACCCGCUGACGUCACCCCCACGUCACCUCCACACGCAGCUCCUGAGGCACAGCU
GCUGCCACCCGCCCUGGGCCGGCUUUAGCCCAGCGGUUCCUUCGGCAAACAGUUUCUGAGUUGCGGGCCCCGACUGGGGUUCGAUCCCGCGGGCACC
CCUGGCCCAUUUU

>*STRA8*, XM_015289566
GUAGUUUCCCUGUUUGUCAAUGUUUUGCCAAGCAUCACUGGAUACAGCAGGCAUGUGAAGAGAUUAUGCCUCUUCCAGAGGAACACCUCUCCAAUCA
GAAUGGGGAGACACUGUGUUGUGGUUUGUUACUGUUCACUUCCCUUUCCUCAUAAGUGCUUCAUAACUUAUCAGUUGAGCAAUUUAUACUUUUAAAC
GUUCCAGGUUAAAAAUAUUUAUAAAACCUAAAGGAAGAAUUAUUUUUCUCUCUGACUUUUUAAUGCUUAACCUCAUUUCACAAAUACACUGAAGCAC
GUGCAGUUUGGUGACCUUGCCUAAGGUGAGCUGUGGAAGAGAUGCAGAGAGGACAUGGCAACCUACGCGCUCCAGACUGCUACCUGCAAAACUCACA
GCGUGCCUCCCAGCAUCCUGGCAGUGAAUGCUUUCCUCUACAGUUCUUUAGCCUGGGCUGGAAACAACAGAGGAAUUUCUUCACAGAGAGCGACAUG
AGGCUCUUCCGUAUCUAUGAGGACAACUGUAGUGGCCACCCACUCCCAAAUCGCUUGGAGAGAUGCAAGUACAAUCACAAACUGUGGAUUAUUUUUG
UAAAGUGUGUAAAGCUGAAAGAGAACAAAGCCCCCUGUGCUGUUCUGCUGCAUCUUGUUCUUUGAUGAGUAGUACUGCUCAGAUGGUGUCUUGAAAU
UAAAAAUGCUGUGAAGUUAAAACAGAUCAUUCACAUCUCUGAGCUGUCAUUAGCAGCUUUCUGCAGACUCAGACAGUGUUCUGGCAGUGGUUAUAUU
GAGGUCCCCUCCUCCCACUUCCCCUCUCAUUUUAAAGUACCAUGUAUAUAUUGCUCUGGAGGGCCUGGGUUUUGGGUCACGUGAUGCUUGAAACAUG
UAUAGGUUUUUCAGGGGAUCUGAGGUCAAAUCAUUCUUUCCUAAAGGAAGGAAAACAAGAGACAAAUGUGUAAAUGUUUGC

>*DDX4*, NM_204708
AGCAAAUUUAGCACUAAACUCUGUUGUAUUCUGUUGUGGUUUAAGUGAACUGUUGCAGUUAAUGUUUAAUUACUGUUAAGUUUUAUGAAAUGCUUUG
UGUUUACAAGCUACCUUGUAUCUCAUGAUGGCGCUUUCAACAAUGAAAAAGGAACCCAUCGUUGAUUCUCACUCAGUGUAGGAAGGCAAAAGCCAUU
UUCAGGAAAAAAAAAGCCUGAAACUAAGUGUUAAAUGUUUCAGUCAGUAGGUGUUUACUGAUGCUUAUAUGUUACUACAAAUAAUGUGUUGCUAUAAA
AGAGUGAGUUGUCAUCCCUGUUUUGCUUGCAAGUGGAAAAUAGCAUAUCACUUGAACUUAAUACUUUGUUAUUAAAAUUCAGUUAAAUUGCAACUUG
GAAGCAGUGUAUGUAUUCAGUGCCCUGGACAGCAUGACUCCUUUUCUUGACAAUUUUCCGCAGGAUUUGUGUAUCCAUUAUGACUUCAGGUCAGAAG
CUUGGGGUCAUCUUGAUUUGCUAGCUGUAGGAAAUACGUAUGAGUAGUAAUUUCUGGAGGGAGAUGUACACUUGAAGUCUUCCGUGUCUAUGAAAAG
CAACCAGAGUAACUGCAGUCAUGGGUCAUCUGUAUCCUAAGAAAAAGCCUUCGAAGGGGGCUGACUUAUCCUUGUUUCGAGGAUCACUUCAAUCACA
GGGUGCUUU

>*NANOS3*, XM_025145794
CGGGACGCAGGCUGUGCUGAGCGCUCCGUGGGACACCAAAGAGCCGGCAGUGGGUGUGCACUGUCACUUGUGCAAGAUCACUUGUGCACCAUCAGUU
AGAUGUGCACCGUCAGUUGUGCACCAUCACUUAGAUGCGCACCAUCAGCACUGCUUGUGCACCACUGGCAGCAAUUGUGCACCACUGGUUGUGCACC
AUCGGCACAACACAACGGCCGGGCCCCACGCUGGGGCUGCUGUUGGUUGGGUGGACGGAACUGUUGUGGGGUGGGGGCACUUUGUUGUGUUGGUGU
CACACGGGGGCUCUUUGUUGUGCUGUGUCCCUCCUGGUGUCACACGGGGUCACUGCUGUGUUGUGUUGGUGUUACACGGGGGCACUUUGUUGUGUUG
UGUUGGUGUCACACGGGGGCACUUUGUUGUGUUGUUGUCACACGAGGGCUCUUUGUUGUGCUGUGUCCCUCCUGGUGUCACCCGGGGUCACUGCUGU
GUUGUGUUGGUGUUACACGGGGGCACUUUGUUGUGUUGUGUUGGUGUCACACGAGGGCUCUUUGUUGUGCUGUGUCCCUCCUGGUGUCACCCGCAGU
CACCGCUGUGUUGUGUUGGUGUCACACGGGGUCACACUCUGGUGUUGGUGUCACCCGGGAUCACUGCUGUGUUUUGUUGGUGUCACACGGGGUCACA
CUCUGGUGUUGGUGUCACCUGUGGUCACCGCUGUGUUUUGUUGGUGUCACACGGGGUCACACUCUGGUGUUGGUGUCACCCGGGAUCACUGCUGUGU
UUUGUUGGUGUCACACGGGGUCACACUCUGGUGUUGGUGUCACCUGUGGUCACCGCUGUGUUUUGUUGGUGUCACACGGGGUCACACUCUGGUGUUG
GUGUCACCUGUGGUCACCGCUGUGUUGUGUUGGUGUCACACGGGGUCACACUCUGGUGUCGGUGCCCCCCGUGUCCCAGGGGUGGUUUUAUUGGGC
CGCUGCAGGUUUUGGUGCCCCCAAUAAAGACUUACAGCCGUCGGCGCUGCUUCA

>*SYCP3*, XM_004937837
UGGCUCAAUGGAGAAACAACUUGUACCUAAGUAACACGAUACAAGUAUAGGCGUUAGCCAUGAAGAAGUUAUCUUAAGAUUUAAGUAAGUGUUUUAA
AGUUCCUAUUAAAUACUUUAAUGGAUUGUUCUAAUCUUGUGUCUGAUAAUGUUGUAAGAAGGUAUAUUAGUAAAUUCGUUACUAAGGGGAAAAAACC
CCAAACCAAUUAUGGUCUGUCCAAUUAAUAUUUACUUUCAUUAGCAGUGGAGUGUUCAAUUGUUUCACUUUUUAUAAUACUCAGUUGAAACAAUAGG
UAGAAUUAAUGAAAAAAAAAGACAGAAUUUUGCAUAGUAACUUAACAAAUCUGGGUACACUGCAAGUAGAAGCCUUUUACAAAAGCCAGAGCUUCAA
GACUUCCAUAGUUUCAACACAUAGCCUCUAGACCUGUCAGCAGUUAAAGUUCACGGAUUAUGAAAGGUGGUUUAAUGAUGUAUGUUAUGGUAAAUGA
AACGUGAGGCCUUCUGUCAGGUUAGUCAUUGUGAUUUCUUUUUACAAUAAGAGAGAUAUCAACUGCUGUAACUUGUCCAAAAAGCAAGUUUCUUUAA
UUGAAUAAUGAGCUUAGUACCACAGCUUUACAGGACAUCUGCUCAAGUAAGCAUAAUAGAAUAUUACUGCAUGCAUUUUGGGUUAUAUAUUUCUGUA
CACCUCUGUUGAGGUAAUCUAUCUCACUUACUUGAAAUGGAAUAUUAAGUCUUGUAGUUUCAAAUGACUAGCAGUUCAGGGAGGUCUGUUUUAAGAA
UAAAGCAUUUUUCAUUUA

>*PUM2*, NM_001039292
AAGGCAAAAAAAAAGAAGGAAGUGGAAGAAAAAUUUUAUUGUGAAUGAUCAAAACAUACAACUUAACUAUAAAUGUUCUGAUUUUUUUAAAUCUAUU
UAUUGACUUUGUUCUCCAUUUGUAA

>*PRDM14*, XM_025147991
CUCUCAUGGUGUGGGUCACAGGAGUGCAGUCUCAAAUACCUUUCAGCGUGCAGACGGCUGGUUUACAGCACGCUGACUUUGUGCUGGGGUGAGCUCU
GACAUGCGCUGACCCUCCUUCCUCUUCCUCUCCAUGCUCCCUCAUUUUCAGGAGCAUCCCUUCCCCAAAAGCCUCUUAUGUGUGAGUUGCACUGUAA
UGGAUAUUACCAGACCUCUAAUUCACUGCAGCUGUGGGCUGCCUGGUGCUGUCCAUGCCAAAGCUUGUUUAGAGGUGUUCUAGUAAGUAUGUAUACC
AAAGACAGCUUAACCUAUUUGUUUUAUUUAGGCACAUGACCAGGUGUGGGUUCUAUCUCCUUGGAGUGUGCAAGUCUUUUAAUGAAUCCCUAUUGAU
AAGAGAAUGCUGCUGGAGCAGCAUAAGAGUAUUUAAUUGUGGCUUCAGGGCCAGCUGAGCUGACCAUGGGAGCUCUGGGGCAGUGACACCGACUCUU
CCUUAGCCCAGGAGAGAGGUGGGGUGUAUGCAAAUGGCUGCAGGUGUGUGAAAUGGUUCCAGCAGCACUGUGGGGUGGUGGCUCUGCCUUCUCUGUC
UCUGCAGAAGACCAAGCUUGAAGUGGUUCUGGAACACCGACAGCUCUCCCAGGUGGGCCGUGGGCACUCUAGCAUUCUGUAGCAGCUUUGCACUGGG
CUUGACACAGGCCAGGGGUAGGCAUGAUUCACCCUAGGCUCCUCUGUAUCAAGGCAUAAAGGCUUUUAGUCAACUCUUAUACCUCACUUAAGGAAAG
GGGGAAAGCCUUUUACCCAUCUUCACUUUAAGAUGAGAUGUGUAUGACAGACAGUCUUGGGGCUUCUCAAUCCACAAAUCUCUCUGCAUGUGACAUU
GCCUCCCCAAGCCAUGGGAAUGCCUUUACUUUGCAGGAGGGCUGGAGAGUUUUAGCCCUGCAUUUCUCGCUGACAAUGCAAACAAAUUCAGAGUGAU
GUGUGGGCUUUCUGUUGAUUUCUUUUCAGCAAAAAGGAAAAGGUUUGCUCAUAAAAAAUGCCUUUUGUUUUCUAUAGGAAAAGCUGCCCAUUUAGCUA
UGGGUGAAGAAUUAGUUUGCUAAAACUGGGUUUGCUUUUUUUUUUGUGCUCUGACUUAAGAGUAACCUCAUGCAUCCUGCCUUUCAGUACCUACAAA
CCACCCCAGGUGAAGCAUUCUCAAGUGUAUAUUAUUCAAUGGCUAAAAUACUUUCAGCAUAAAAUCCUUAGUGAUACUUUUUGCAAAAUAUUCCCCA
AAUCGGUAACUGUGGCAUUCCCUGGUAAUGUGAAAUCUCAGCUUACGUGGAGGUCUGCACUGACAGAGCCCUCCAUCUGUCAGUGCUGGGAAGGGGC
UGUUUCCUCGGGAGGCUCCUGUGUCGGUGCAGAUAUGUGUGCUGUGUUGCACACAGGAGACAAUAGCAGCCCUUGCUGGAGCUGAUUCUUCAAUGCA
GGUAAUUUGGUGUUAUUGGGGUGAUAAGGUUCUCUUAACAUCCUAGGUCACCUAGGAUUAACAAGGACUUUUUGAGCUCAAGUACAGAAACUGAAUG
UGUAUUCUGUGCCCAACAACUUGAAUCUCUCUUUAAAUACAGUAUAAAACUAGGAAAAAAAAAAGUUACUUGCACAUUCUGUCUUGCAGAUACUGUA
UAUGUACUGACUUGCACUAACUUUUAUAUGGCUUAUUGUAUUUAAGUUGUGUUGUCCUCAUUUUCAUGGUGAAAAUUGAAAAUAAUAAUAAUAAUAA
UAAACUAUAGAAAAGUAUUGAGGCCCAGCACUGGGCAGUAUCUGAAAUAAGCAAUCCAUAAAUGAAUGCAGCUGAGGGCACUGAGCCUUAGUUAUGG
CAGCCUGCCUACAUACAGGUGUUUACUCCCAUGCAUCUACAACCCUGGGUGACUGAAACAUGAAUUGUGCAUGGGAAGGCUUUGUUGGUUUGGUCCC
AUAAAGAACUCCAGAUUUGUUGAAAUCAUCAGCUGCUACUGAAGGAAACUGUGACUCAGACUGGAUGAGAAGUGCUGUGAGGUGCUUAGUUAGCUUC
GCCUGUGGGUAACCUUGCUGUUUCUUGUGAGUGGGGGUUAUUUAUUCCAGUGGUGCAUCCUCUGCAGUGCUUUUAGCUGUGUGUGUUCUCCCCUAUG
UGAAGAACCCCCAUCUACCUUGUCCUCAAAUAUUAUUAGCACUCUUCUACAGCAUCGUGUGAUGAGUUGUCUGCAAAAGAGCAUGUGAAGAGUUUUG
CUUGUGCUUGUUUCUCAAGGUACUAAUUGUCUUGCACAUUAAACUCGUAUAUGUAAAUGUUUUAUGAUGAUUAAUUUCACAGAAAGGAAGAAAAUAA
AUUAUUUGAGGCUCUGGUAUACGUGAA

>*DZIP1*, XM_015274761
UAGUUAUAUUCGUGUUGGGAGGCGCUGCUUUUUGGUUUCUAUUGAGUGUGAAAAUGUAAUUGAAAUCAUCGAGUGUUUCCUGCUCAUUUCUCAUGCU
GUUCAAGUGACAACAAUGAAUACUGUUUACAGAGCUCCUGUGUCUUUUAGCCCUGAGUUUGUACAAAGAAUUUUCAUAUGCACAUCGCUGGCCAUCA
GUAUUUGGAAACCAAGAAAAUUGUUCUUCCUUGUGAAGUCAGUGCUGAAAAUCCUUGAUUUCACUGAGGUGAAGAUUUUGCCUCUAUUGUUUACUUU
AAAGACCAAACCUGGUAUUAGAAGAAAACCUGUUCACCAGUGAACAGAAUUUACUGCAGAGUAUUACUUUAUAAGAUUUUUUAAAUGCAUAUUUUCU
UGUAUUUUAUCAUAAACUUUAUAGAAAAUGGUGAGCAUUUUAAAUAGACUGUUUCCAUGUAUUCAAAUGUUAAGUUUCUAACAUGGUUUACAAUAAA
CGCCAAAGGGGAAAAAAAGGCCUCAUAAAUAAAAGUUACUACUAAAUGUCUCCUAUUUUGUGAAGUCACCCUUUUUCCCGCUAUUAGAAUUAACUCU
UAGUUAACAUAAAAGCAGAAAAGAAAAGAAAAUGAAAAAAACACUUCAAUCUGUUUUAAGGAAAAAUAAUGACCUUUAAUUGAGCGCAUACAUCCAU
ACUUGUUGUGUGCCCACUUACUUGAAAAGACACAGUGAGGGUUUGACUUAAGUUCAAAAGCAAAAUAAAAGAAGCCAUGAAUGACUUUGUAAUUCCA
AUGUAUUUUAGGUGGUGUCAGAUAUAAAAUUUUCUGUGUUGCUGUGCCAUUAAUAAAUUGGACUAUCUACUGUUUGUUUUUA

>*TDRD*7, NM_001305104
UCGCCGCUUCUGAAACGUAGCAGCUCAGAUUCUGUAGCAAUAAAGUAAUAGGCUUCAAAGAGAGAUUUAACUGUUCUUGUAUUGCCUUGAAGAGAAU
AAGGGGAUAAACUUCAAAAGCACGUGUUCUGUGAAAAAGAUCUGCUUAACAAGUUUGCUUUCUGUUGACUUCAGUUGACUUUAUGAAGUUUUGCACU
UUGUUUACUACUGUAAUACUAGACCGUUUGGGUGGAAAUACUAAAAAAGUUAUUUAUCAAUAAAACAGUGCUGAACUAUU

>*PUM1*, NM_001012840
GGCACGGAGCUGCCCCGGCCAUCCGGCAUCCACAACCAGCAACCAGCCCUAUUCCUAUAUACAGUUAGAGAGCGCGUUACGGUUGCUAAACUACUAA
AAGAAGAAGAAGAAAAAAAGCCUUUGUAAAUUUCUUUAAUUUUAUUAUGCAUAACAUGUACUAAUUAUUUUUUUUAAUUAACUGAUUGCCCUGCUGU
UUUACUGGUGUAUAGAAUACUUGUACAUAGGUAUCACAUGUACAUGGAAGGCCACAUUUUUGUUCACUGUUGUAUCUAUAUUCCAAAUGUGGAAACU
UUCAGGGUGGUUGGUUUGAAGUACAAAGAAAAAACACACGACGUUUUUAAUCCAUCUGCCUUGCAGGCAACUCUGUGCAGAUACCCGUUGUUCUCCU
CUGACUCAGAAGUCAGCAGCAGGUUUGGAUUUUAGUUGAACGGCGCGAGAGACGCAUUUAAUGCUGUUUAUAAAUAUUAAAAAGAAAAAAAAAAAA
AAAA

>*POUV*, NM_001309372 (3’UTR is not known)

>*NANOG*, NM_001146142 (3’ UTR is not known)

>*NANOS1*, XM_025151916
GAGCGGGGCGCGCGGGGACUGGCGGGCGGCUUCGGCGCGGCGGGGACGCCCUGAACCCUUCUCCUUCUCCCCAUCUCUUUCUCGAUCUUUUGAUGCU
GACGGGGGAGCAGAAGCUGGAGGAGCAGCAGGGGCAGGAGCUGACCGCCGAACUGUUUCACGUUCUUCCCCACGUUCCUCUCUCCUAUCGUCUGCAC
GCCGCCAUGAGCUUGCUACAAAAA

>*PRDM1*, XM_004940353
GACUUUUUGGCAAAGUGAUUUUUUUAUUUAUGACUUGGCAAGUCAGGGUGCCUGUAGCAGUUGCUUGUACAUAGUUUCAAUGCUGCAAAGCAAUCUU
GGCUUACAGUAGUUUCCCUACGCUCUCCAGCUGAAAGAAGGAACUCCAAAGUUACUGUUUUCUCAGGGCAUAAAAAGAGGCAAAGGACUGCGUGUUG
CCUCGCCAGUUACUAAAAGACAAUCAUCUAUCCAUAAUUAUUUUUUCAAUGAUAGUACUUCAUAAUUUAUUAUGCAAUUUAUCAUUCUAAAAGCAAU
AAUUAGAAAAAUGUUUACAAUGACUGGGAAAUUCAUUGUAAUUUUUACUUUAAGUGUUUUUAUUUUUUGUUUUAUGGCCAUUCUUCAUUGAGUUUUU
UUUCUGCACAUCUGUUUUAAGAACCUAAGAUUGGGGUUUCAGUAACCAUGUUUUAUGUACCAAUGUCUUUUAAACAAAUGUGUUUUUUUCUAUUGCC
AAAGUUGGACAUUGACAUACAGAAUCCUAGAUCAGUCAGACUGAAAAAAAUGCUGGGUACUUUUAUGUGCAAAUCACCCCAUUGGGAUCGAAAAGGA
GAAGAAAAAAAAAGAAAAGGUAGCAGCAAGCAGAGAAGAAACUGCUGCCCCGGCCUCUCUGAGGGCAGGAGAGGUUCUUCUACCUGCAGCUCUACCC
AGAACAGAUGUGACACAGCAAACACCUGCAUGGACGAUACCGUAGGUUUUUAGAGAACAGCUAUAGGCCCACACCUUGUCACAUAACUUUAUGACCA
AAGGAACGCAUCAGCUUAAAGUGGCUCUUCCAAAAUAAUAUAUCACUUACAUGCUUUUGUUUCCAACAACGGUUUAAAAGCAAACACACCAAUAUUC
UUAAUAUUCUGCCUCAAGGGAGAUUUUCGGCAGAAUUCUUUCUUUUCUCCCUCUUUUUCUUGUUUUGUUUGUUUUUGGCCAGAGCCUUCCAAGGAUA

CAAAAAACAGUCAGACUCCCUGAGAAAUAAGGGGUUUGGUCUUACAUGUGUUUUUCUCACCCGCUUUGCCUGGGUGUAGAAGUGGGGAACAAAGUCC
UUUUCUGGCACAUACAUAUUUUUUCUUUCCUUCCUGUACAACUCAGAUUUUUCUCAGUAUUUGUGUUUGUACAUUUUGUGGUUAAUUUAAUUAAAGA
AGAAAAGGGCAUUGCAAAGUUGUUGAACAACAAUUACCUCAGUGCUUGUGUCCAGUGGUGCAGACAAUGCUGUUUUAUCUAAAUGCUUUGCUACUUU
AGAGAAGAAACCAAAAUGUGCACAGGGAAAGAUAGAAUGCACGUCCUUUAUCUUUUUGUUUUGCUAAGCCCAAAGAUGAUAUGGUGACAUUUGAGGU
GUAGCAAAGAGUACAUGUAUAUUUAUAGAUAUAUUUAUACCACUAUACUAUAUAUGUAUAUGUAUAUAUAUUUAUACCACUUAAGUUGUGAGCCAAA
CCAUGUAAUAAAACCUAUUUUUCAUCUAAGCGUGAAAAUCAACUGUUAUCCAGUUUUGCAUAUUACCAAUGACCUCAAAACCAAAAGGUUGUACAAA
GGCAUUUAUCGCUUGCAAGUACACAUGCAGGGCUGUCUUUGCAGGGCCCGAACUUGCAAACGCCUGAUGUGAAAAACGUCACUCACAUGGGGAGUCU
GGGCAAAGUCAGGGGGAGGAACCGUGUCUGCAGGACUGGGGUCCUCAGUGCCCUGCUCUCCUGGCAGUGUGUACUGGGGGGAAAAGAGGCGGGUGGG
CAGUGAACGAGCAACCAGAAAACUUAACCAAAAGAACCCAAAAAGAAGCCAAAUACUACGCAUGCAUGGUGUGAGUGCAGAGCACAGUGAAGGGUAG
GCUGAAAGUCAGUGGGGGCGAGCUGUAGUGAGCCCCCCAGUCUCAUUAUAUCCUGCCAUUAACAAGUUGCAUCAUCAACUCGCAUCAAUUUCAGUAG
CAUUAGCACAGUCUUCCCCUCCAGGUGCAUCUUUUUGUCUGUUUCUAUUACAAAUUGUAAGGUCUCCCACUUCAUCAAUCCAGGAUUUGGUGAUAUU
UUUUUCCCUGCAGAACUUUAUUUUAAUGCCUUUGGUUCAACAAUCCCAACAUACACACAGCUUUUGAAGGACUACAGUUCAUUACAUUUCCACUCCU
GCAACUUGUGAGGCUGACAUCUUUUAAAUGUAGCAAUAGUUCAUAAAUAUAGUACGUACUUGUAGGAUAAACCAAAGUAUCAUUACUCUGUCACUGG
ACACUCACUUUUCCUAUAUUUGCCUGUAGUGCUUUCUUGUAUUUGAUACAAAUUUUACAAGAAUUUAUAUGCAUCAGCUUUAAGAAUUGUUACUUCU
CUUUACUGUUUCCCCCUUCCCUUAGAAGUUUUACAUGUGAAUGUAGCAACAAUCAACAGUGUUUUGGGGUUUGUUUUUUUUUUUUUUUUUUUUUUU
GUCUCUCUUAAGAAAGACUCUGACCAAAGUUAACAGGCUUUUUACUUUGCUAGAACAACAAACUAUCAUAUGUUUACGUAUUGGUUUACAUCAUUAU
UUAUGUGCAAAUUGUCAAAUGUAAAUUAAAUAUAAGUGUUCAUUGCUUUA

>*DDX25*, XM_015302207
GCAAAAAACAUGGCUUAGCUCCAAAGAACUCACAUUUUGGUUUAAAAUGUAUGGGAAAUCCAGGAGAAAAUCUCCCAGUUUUCUAGCUUCCUGGAGG
UACUUGGUUUUGUUGUGACAUUUUAGAAAGAGAGCAGGGGAAAAACCCUCAGCCUAAAAAAGUUAUUCUGAUUGGUUUCCUUGGUGUUCUGGGGGAA
UGUACCUUGUUUUGUGGAAAUGCUCCUUGGUUUUGGGGCAGCAUUUGCCUGUUUUGGGUACUGUUGCUCUGUUUGGGAUACUAUUGCCCUGUUCUGG
GCUCUUUUCCUUGUUUUGAGGCAGUGGUUUAUAGUUCUGGGGCACUGUUUGUUGUCUGAGGGUACUAUUCCUUGUUUUGUUUUUUUUUUUUAUUUGU
CUCUAAAAGAAAUAAGUUUAAAGCAGACAAGUCCCAGUUCAACCAUCUUGGUGUCUGAGGUUUCUGCGGAAACUAGUCUUGAAGAUAGCAAGCACUG
UUCUGUAACAUGUUAGUUUAAGGGGGUGUGAAGAGGAGUUGGAAGCUCUGGGGAGCAAAAAAACGGUUCAUUAAAAAUAAUAAACAACAAACAUAAA
UAAUGCUA

>*PIWIL1*, XM_015275259
UUGGUUUAAAAAAAAAAAAAAAGUUCCUUUCUGGGGAAGGUGGAAGUGUGGUUUGGGUUGGUUGGUUUGUUUUCAUGAGACACAAGCAGGGGUCCAUG
CAGUUGUGGAACUUUUAUUUUCACUGUAGCGGGAAAAAGAUUGAAGAUAGCAGAUCUGUGUUUUGAAUAAGACUUUAUUCAACUGCAUAGAGGGAAA
CAUUGUUAGAGACUGCUACAGAUUCAUAGGUGAAUGAACUCAGUUUUGUGGUUGACAUUGUUCGUUACUUUUCUCAUUUAAUAAGCAUUCUCUUGAU
UUCCUGUAAGGAACAUCAGUAUCUACGUCAGCACUGCCUUGAGAGGGGGAAAAAAAAAAAAAAAAGAGUUGUGCUAUUUACAGGGCUUUUUGGUUUU
UCAGCUCCAGUAAUUGGAAGGGCUUAGGUGUCAGUUCGGAUUGUAAUUGUAAGGUUCUUGCUGGUAACUGAGCAGGAGUGGCUUAAUUUGUAAGGGA
AAACACGGAGUACUUCAGAUGUUGUAUUACCAGCUAUCCCGUUAAACUAGAUGAGACAUAAGUGUUUGGAUUAGUGCUGUUUUUUGAAACUAACUUG
UGAACUUUGUUACUUUUCCUUGCAAAUUCUAAAUAAAAUUGUAAUCCUGC

>*YTHDC2*, XM_004949271
CACUAGAAGAAUAAAAGGAAAUAAAGUUGAUUUUAAUAUUGUUCAUGUAUUAACAAAAAUCAUAAGAUUAUUCACCAGCCUGGUUUCAAGUGAAUUA
UUUUUUUCUAAAGUAAGAUAUUCAGUAUGCAGUUGAUUACUCAGAAAUAUGGUAAAAUUCAAAACUAAAUUUGAAACAGUGCACCAUGCACUGGGUU
UUAAAUAGCACAGCAAGUACAGUUUAAACUGUUUAAGUACACAGUCACAUUCAAAGCUUUUUCAACAUUAGUAGUAAAGAAACCAGUGUAAAAUUAA
UUAGUCUUGCAGGUUGCUGAAAUUAAAUACAUUUCAUUUACUGAAAUGAAUAAAAGAUUCCACUAAGUAUUAGUUGAAAUUCAAUGUGAGAAAUCAA
UAUGUAUUCAUGCAAACAAUUGAGCAGUUGGAGGAUGUUUAAAUCUAUUUAAACAUGUAAAUAAAGGGAGGGUGAGACUCCUUUGAGAGUUCUCUUA
UUCAGUGUCUCCUCUCAGUCUGUUGUUUUACACUUUUUUAAAGAUGAGUUUAGUUUACUAAGUCUCAAUUGCUUGGUAGUACGUGAAAAUCUAAUAG
GGCACCAGUGUGGGCAUUCUGAGAAGAUGAUUUUGGUUCAUUUAAAAAAAAAAAAACAACUGCCAGUAUAGCUUUCUUUGUUUUACUGAGCUUCUCU
UUACAGGUUAACGUUUUGUAAGAUUGGUGCAAAUUCACUCUGUCUGCUAUCAGCACAAUGUUUGUAGUUGAUCUUAAAAUAAACACAGAGCCUUUUC
UGCCUCUGA

>*SDAD1*, 420597
AACAGUAAAGGAAAGGAAUAGUUACCAUCCUUGAAGAAACAACUCUAGAAAUACUUGUUUUCUCCUCUCUCAAAAAAGUAGGGGGGAAAAAAACAGA
ACUGAAUUGCCUUGAGGAUUCAUUUUGAUUCCUCUCUACUCUGUUCAGAAUUAGAAAUUGAAUGCUGCCAAGUUGGAUAUUGCAUCUAUAGGACACU
GAAGAAGCCUUAAUCCUCCUAGGAUUUUUAUUUUUAAUCACCUUUCCUAGAAUGCUCUACCUUUUUAUUGAAUAUAAUUUAUUUACUGCUGUUUUGU
GUAAGUAUGAAUUAAAACAUACAAUUUUACUAUAAAAAAUAUUUUAAUUGAUUAAAUAAAUGAAGAGGUAAUUGGAAGGAAAAUGAA

>*PABPC1L*, XM_015296733
GCCCGGGCUUCAUGACUGAGAGUGAACCUUGAGAACGUCGCCUUUGGAGAGAGCAGCAGCAGCCGCCUCACCUCACCUUGCUUUUGGAACUGUGCCC
UCACACUGACCCUAGUUUGCCUUCAAUGCCCCAUCCUGCCUUUUGUGUGUACUGUGUGUCUUGCAGAACCACUUCCUCCUUGCACUGCUGUAGAAGU
UGAUUCUCUUCUCUUCUGAAAGCACCUCAUGAAAUGUGAUGUGGAACUCAGUCUGACUCAGUUGCCCAGGAAAUGACAAGUGCAGAUGAAUCAUCAC
UUCAGUGAAUUUAUUGGGUUCUGUAGUUUUAUUUGUUCCUGUAGUUGAUGCUAAUAAAGAACUGAUGCUUCCA

>*ZP3*, NM_204389
GUGCAGGGCGAGCCGUGAAUAAAGCCUGGAAAGGACAAAAAAAAAA

>*ZBTB16*, NM_001321488
GGGACAUGGAGGGGACGCGGGGCUGAGUCGUGGAGGACUGGGCAAAAAAAAAAAAUGCAUCGUCCAUGGC

>*ELAVL2*, XM_004949537
CAAGUUAUUCUCAGUGCAUUAACAUUUGAAAUCUAUACAACAAAGGCAAUUUAUGAGAAGCUUUCUGCUAGUAAAUGCCUUUGUUGUAUAUCGGUGU
GGAUAUGGGGAUAAAAUGACUACUUAGCAUCCUAAGAAUAUGUGAGAUUUUUUUAUUGCUACUAUUUGAAUUAGAACUUCUUGAAUACCUUUUGUGU
UUGAAUAUUGAAAAGAGGUACAGGGUUUUUACCUGUCACAGUGCAUUCUAUUGCCUUCUUUGAAGAAGGUUGACCUUUUAAAAUGUUUCAGCUAAGG
AAGAAUUUUUGUGUUUGUUUUUUUUUUUUUAUUACAUGAUUGCCAUUAACCUAUGAGUAAAUAUUUGAGGCUUUGUGUUAGACUAUUUAAGUUUGUUU
UAUAAUAUCGCAAUCCGCCUUGUGUUUAAUUUACAAUUUGAUGAGUUGCUGUUGUUGUUGAGAAGCAUUUGAAGUUUACAUCUUAUUUAUAAAGUUA

UACGCAGUAUUUAUUUUCUAGUUAGUGUUUGGGUUGGGGAAGGGGAACACAUUAUAGAAGCUUAUUGUAGGAAUACUGGAGAACUUUUCGUAAAGCA
GUACCUUGCCAAAGAGAUAAGAGCCUCUUUGAUGUGGGUUUAAAAAAAAAAAUCAUCUAUUUUUAUAAAAAAUAAAAAUAAAAAAAAAUUUGGAGAAA
CUUUUUACUGGUCCUGGGACAAAUAUUUUGACUUGAAUACUUUGAGAAAUCUCUUCAUAUGACACCUUGUGAGCUUUUAAAACUUACCAGGAAAUUU
ACAGUUGUUGGGGGAAAAUUUAGAAAGAUUUAUGAUGUAGAAAUAAAUUUGAGACAUUUCUAUUAAUAAGUAGAGUUGAUGGGAUGCACUGUUGGUU
UCAUUUUUGUAAUCAUCAGUGGAGUUUUUGAUCAUCCUGGUUAUAUAAGUGGCUCACAUAAACUUGUGAUUUUGAAACAAGAGAAAACUGAAAAAAA
AAAUCUAUAUAAGAGCAAACAGGAAACUUAAAUACUUAUGAGACGGGGGAAAAAAAAAUCUGUUCUUCCUAAAGAAUUCCCUUCAGAUAGAGCUCAU
GGUGUUUAGUGAUGUACUCGCUAUUGUUUGAAGAGUUGUUUUGUCUUAGGAAAAGACAUUGUGCAUGGUUUGCACAGCAGUUGGGUAUGUUUGGAGG
UAUUUUGAAAGUUAUGUUCAAGGCUAACACCUGAGCUUUGUGUAUUGUAAAUAAGACCUUGAGUUAUGAACCUUUCAGCUAAUUUUGAUUUUUGUUU
AUUUUUCAACUUACAUGCCAAGUGAUGGUCAAUUCUGAAUGUUUUUGUAUGAGUUUUUUCUUUGUAAAUGGCAUUAACAUUGUUACUUGAGGUCUGC
UUAUUCAUUUUUGUUGUCCUGAGGACUUGAAUUUACAGUGCAUCAGAUCUGUUGCUAUUUUUGUCUGUAGAUAGUCUAGCUUCCGCUGUUUAUAGUG
AUGCUACAUUUUGGGUUAUAAAUAUGUUUGUGGUUAAAAACGAGUAUAAUCAUAAGUUUUGAACAAAUUUCCUUACAUUUUUCAUACAAAACCCAUG
AAUACCUGUAUGCUACUGAAAUUUAACUUUGUAUAAUGCUUAAAUCACUAUUUGGGGACAUAGAAAGUCUUUACCAUGUAUUGAAGAAAUUAAAGAA
UUACAGAAUAUUUUCUGAUUAGCAUCUAGCUUAUAUUAAAUUUUUGAGGAGGAAAAAAAAAAGCUGAGGGUUUUGAUGCCUUCGCCAGGAUGCACUGA
CAACUAUAUAGUUACGUCCUGCUUUUUGUAUAAACUGAGAUGCUCAUACGCUUCCCCUUAGAAAAAAGCGAUGUUCUAUGCAUAAACAUAGUUGUAC
ACUAUCUUUGCAGUUGCGUUUGGUUCUUAUUCUUUUAAACUGUAGUUAUAAAUUUUUCAGUAUAGUACAGUACAUAUACUGUGAAGCGCGUGCUAAA
GUGAAUAAGCGAGUUUUCAUGCUGACCCACUCAAUGCUAUUCGAAAAUCAAUUGGCUUCCUGUGCACUUUCUCAUUCUUAGGUGCAUUUAGAUUGUU
AAAGCUCUUCUGCGUUAAAUUGUAUAUACUGUAGGAAACACAAUCUUCCCAAUAUAGGGAAAACAGUAGCAACAUGCUUCUUUCCUUAUACUACUCU
AGCAAACAACGGCAUUGAUGAGAAGGCAUGUAAAUUGGGCUUCACUUUACAGUGUUAUCAGAGCACUUAGUAAAAUGUAAGGUUGGUAUUUAUUUGA
AGAUGUAUAGUUAAUUCACAUCUGUUGGAAUAGAAAAUAUAUUCUGUUGAGUAUUUAAAGAGGUUGUACAUGUUUUCUUUUGUGUUUGGAUUCUUUG
UACUUUUUCAUGUUCAGUACAUCAAUAAACAAAGUUGAAGGGAAAACA

>*PIWIL2*, XM_025142807
CCCGGCGCUGCGGCGCUGCUUUGCUUCCUUUCCGUGCGUUUCUGCCCGUCGUUCCUUUGGGGCGUUGGAAAGUGAGUGGGGGGGGGGGGGGGCGGUG
CGCGC

>*NOBOX*, XM_015292279
UGAAAAGUCCAGUAGAUUUUUUUAAUUUAUUUUUUAAAUGACACAUGGAGGUGAAAUAAGGAAAAAAUACUAAGACAGCAGUAGCUCUUCUGUGUGC
ACUUGUGGGGUUAUGAAAGCAAAACUGUGGCGUGAAUGGGAAUUCAAUUUUCUUUUUUUGGGUUUUGACCCAAGAUUGGCCCAAUUAUCCAAUAGGU
GGUUCUCAGAUUCAUCUCUUCUUAGAAAUGGAGAAUAAAUGGUAUGAGUAAAAUAGAUGGCUAAACUCUGUCUUGUAUCAGACUUACUGCAUUUUAA
GCUGAGCAGCUACAUAGGAUUGAUUAUUUCUACAUGACUCCUCAAGGCAGAACUUCUCGAGAAAGAAGAGAGGAAGGGAUAGCUUUUUGUUUGAUUU
CCCAAAUGACUUUUCAGAAAAAUACAUUUAUUUUUAGAGACAAAAUUAUAUUGCUCUAGAUUUGCUGUAAAAAGUUGGGGGAGGAUGUUUGUCUAAU
UUUGCAAUGUGAAUUACUUGGAAAAAAUUCAGGCAAUUCUAGGCCAUGUGAGGAUUGCAUUUUAUUAACAAUCUGUUUUUCUUCUAAUGUUAAAGAA
GAAUUGAUUGAUGCUGUGCUCUGAGAAAAAACAAGAGGAAACAUUACUAUUUGAACUGCAAAAUAAGAGUAGAGAAGAGAAUAUUAUUUGAAUUUUU
UUUAAACUAUUAUUGCUAUUAUUGUUUUCCUGCUGAGGAAAGAAGCUGAAAAAUUUAAAUCAAUCUAACUUCUAGUCUGUGUUGUGUUUUUGUUGUU
GCUUUUUUCCAAAUCUGACUUUAUUUUUAAUAAUUAAUUAAGAUCUUGAGGGGCAUGCAGAAAGGAAAGACCUGAUACAGGCAACAGAGUCCCCUGG
GUAGGUGGAAAUUCCCAAGAGUUUGAAAGAAUGGCUAGUAUCCUGGGAAGAGAGCCCAGGGAGAUUAAGUUGCGUACAAAAUGAGGAGGAACUCCAG
CAAGUACAGCUAAGUUUAGGAACUACAGACAGUACAAGAAGUGUUGUGAGAAGUCCAGUCCAUCAUUUGUUUCAUGUUUGCCAGCUUUUAAAUACAA
AUACUAUAAGAAUUAAUAUAUUCAAACUGUAAUUGUCAGCAAUAUAACCAAAUCUCUAUCAGUUAUUAGUUGGUUUGCAUUCUGUUUGAAUUGCUGA
GAAUUAUAUUGUUUAAACUUACCUAGGAAUUGGUAACAAAUAUUUGGUGGGUACCACUUUCAUGAGAUACUAAGAUCUGACACAACACUUGAAGUGG
CUCAGUUUACUGCAAGAUGAGUUAAGCUUUUAACUAACUUCUUCAGUUAACAGGAACUGUGGACAGUUCAUGCGUUAUGCCUUGCAUUUGCAUCUGC
AUCCUUUCAGGAUGUGUUGCAGUUUAAUCUGAUGGGAUGAUUCAUAGCUGUGGCUUUUCUCUCACAUUCCUGCAAAUCUUAAUGCAGGAACACCAAU
CUGCAGUGUUAUUGUGAGUAAUUUAGUACAGUAAGUGAUCUUGAUUCAGUAGUCUUGGAUGCUGCUUUACCAGGGGCUGUCCUCCUAGGAAACAGAU
AUAAAAAAUAAAUCUCUGAAAUUAGUAAUCAAUGGUAGCAGCAGAAGACUUGACUGAAGAGGCUUUAUACCCUUGUUCAGAACCAAAUGGUUACAGU
UUCUAGGAACUGUGGCUUCCAAAUAAGCUUUAACCAGCUGAAUGCGUAGGAUGUAUGAGGAGUGUCUAAAAUGUCAGGAUCUCGCUGUGUCCUUCUG
AGGACAAACCAUUGUUUUACAUGAAGUUGGCAGUGCAUUUCCUCUGGUAAUUUCUGUGUUGAACUGUGAGCCUUUUCUCUGUCUUUUGGACACAGGC
AUGGCUUGAGUAAUUUUUACAUUACCAUGUUCUAUAUGCCUCUAUUGUACUCUACGAAAUUAAGCAUUGUCAGGUCUUCUGCUUCAUUUUUUUUCUG
UACCAUAAAAGAUGUUGGUGGGAGUUGAAUGCUGCUGGUCCAUUUUUUUUUCCCAUUUCAUUGUAUUGAACAUUGUACAACACUAUCAGUGGCCAAA
GGCUAAGCAGUAUAGGCAGGAACAGAUUUAUAUCAUGAAUCUACAAAAUUGCGAAUGAAU..... (truncated)

>*SYCP1*, XM_025143817
GGGAAGUAGGUUUGUUUCCUUCGUGGUUCA

>*BMP15*, NM_001006589
CCUGUGCCCGGGGCUGGAUUUCUUUUGGAUGUGUUUUGAUGCUUGGUGGGUGGUUGGUGUUUUUUGGGUUUGUUUUUUUUGCUGGGGAGGGGGUUUG
UCUCCACUCGAUGCGAUGCUCUGAGGGAAAGUUUUACAGUUUUAUGGAAGACUUAUAAAAAGAAAUGAUGUAGGAUUUUAAAGGUGGGAGCCUGCCC
UUAGCAUCCCUCUGUCCUGCACUGCUGUGUGUUGUAGGGGCUGGAGCACGAAGCUGGUGGAGAGCGCGUCUCCAUGCUGUCACCUGCACCCAUAGGA
CUCAGUCACACGCACCCCAAGCCAUCUCAUCCCUGGGUAUUUUGGGGGCUCCCCCCGUUGCAGCCCCCCUCCUGGCUUUCCUUGGGAUGGCUAUGGA
ACCUGGUGGGAUGCAAGGAUGGCAGUGUCGCCCCUUUAGAAAAAAAACCAAAAAAAAAAAAAAAAAAAAA

>*CAPZA3*, NM_001278118 (3’UTR is not known)

>*GDF9*, NM_206988
UGGGUGUACCCUAACCUGUCUUACACUUAGUUUGGGUGUUUUGCUUUGCUAACAGCUCUUUCAAACUGAAUAGGGUUUAGCAUGGAGUUAGUGGGUG
UUUAUAUAACAGCACUGUUUUUGUAUCCACUCAGCCUUUCUAGAAUGUGAAAACAAAUGUAAAUAUCUUUUUAUUGGAAUAAUAUUGCAUUUGAACA
GAAGUGUUUUCAACUACCAGAUGCCUCUUUUAAGCUGUUUUGUUACAGAUUUAAUAAUAAAUAAAGUGAAUGCCUAUCACACACAUCACUUAAAAAA
AAAAAAAAAAAAAAAAAAAAAA

>*SNRPC*, NM_001293166
AGACGUGGAGUCCUAUGAUUCUUUUCAUAUUAUGAUCUCCCAUGUGCUAAAAUGCCAUAGUCCUGCAUCCCAAGGAUUUUAUAUGAAAACAGUGUGU
AGAUCUGCUCUCAUUUCAGAUUGCAGACGGUUUGACUUAGAUGCGCACCGGAGUGAUGUUUUUGUAACUGGUCUGGUUUUUGGUUUGUUUUCCCUGU
UGAAAUAAAACAUUGAAGCUGGUUUAUGUAUGGAAAA

>*LIN28A*, NM_001031774
UGGCAGGCGGCAGGGAGGGCUAACACCAGGAUGGAGAGGCUUUGGCAAGGCAAACGGGGAGGGAUGAGGGCAGAACCUUAUUCUCUUUAAAGCCCGG
CUUUAAUUGGGAUGGCUCCAAAAAUAGGAGGGGGAGCAUCCAACACACCUCUCCCGGACAAAAAACAAAUAAAUAAAUAAAUUGGUUCAUCUUUCUC
CAGGAAGCUAGGAGGAGGAGCAGGGAGGCACAGCUCAGGGAGGCUCUGGCGGCUCCUUGCUCUUCGCCCCGUGGCCGGAGCAGGGAGAGACGAGGGU
GGCAGGGCUGGGGGGAAUGCCCGCAGGCCGGCCCCAUGGCCCCCACAGCUCCUCGUCUCUGAAACACAGGGCUGGAAGUGCUCUUCUUUACCACACA
GAGUAGCACAUGGCUCAGUGGAGCUCAGCCUUCUGCGUGGAUCAAACCCACGUGGGACAAACACUGCUGCUCCUCCUGCUCACGCUGCACCAGAACC
CUGGGCUAAGCCUCCCAUGGGUACAGGAAAACAGGGACCUGGGGUGGCAGUGGCUGCCCUCAUCCCUCCCCAGCCUUGCCUUUAUUUAUUGGCCCCA
GUGACAGCCCUUCUUCUGGGGCCGGAUGCCCCGAUUGUGCCAAAGCCAAUGCCAAGUGAAAGCUGCACUAGGAUGUGCGUGAAUGACGUAUCUUUGG
GUUUGUUUGUUGUCUUUGGUUUGUUUUUUUGGUUUUUUUUUUUUUUUUUGCUUUUUAUAUAAAUAUUCAGUUUUUGUAUUUUUGUAUAUUUUAAUCUU
UAAAAAAA

>*SMC1B*, XM_416467
AACAUGGAUGGAUUCAGGGCCAGGCGGCAGGUAGAACUGUGUUUACAGCUGACUGUUAAAAAUCUGAGUUUGAAUUGUGGCCAUUAGUGGUUAAUAG
AUACAUAUUUACCUUUAAAAAAAAAAAAAAAAAGCAUUUUCUUCAAGUAUUUGCUUGUUUGUUCUGUUGCCUUGAGAUGAAUGAUAAAAACACCAUUA
CUGAGCAUAAGACCUGUGUUUUUUUUUAUGAGAAGAGAUAUGUUUAGUUGUAUCUCCAGUGCAUGCUAACACUGGAUUGAUCUCGAGACCUUUACAG
UCUCUUUUUUUAUGUUGCAGCUGCCUCUGUUUACAUUCAGGUGAGAUUAUUUACUUUUUUGAAAAAAAAAAGCAAACAAACAAGAUACUGGAAUAUG
GCUGUUGCUUUCUCUGACUUUUGCAAUAACUGUCAUACAUCCGUUGUCCACUGUAGCAUUAUGAAAAUAGUCAACUUUACAAAAGCUCAGCUCAUCC
AGGUUUGAUGUACCCUGGAAGAAAUUCCCAAGGCAUUUUCCCCCAUACAAAAUGGGGAAGAAUAAAACAGGUGUGAGGCAGCUGCCAGACGAGUAAC
AGCCUUAGAAAAAAAAGCAUCUUCUAUUAGUUUGCAGUUGAGAAGACUUAAUAUGUAGGUGGCUCAGUGCAUUCCAGGUGGAUGACUGUGCUUCAUC
UAAGAAGGAUAAUGAAGUUGGUUACAGAACAUAGUUUGAGGUCUAGAAUCUCUUAAUCUGGCCUAUGUUGCACAUUGGUGUUUACUGAUGAGCUUUA
UCUGGUUUGUGCUCGCUCCUGAUUAUUUCUUGUAUUCAUAUACCAGCUUCCUGCAGUCUACCAUGGCUGUUAAAUACUUAGACCUUGUGAUGUGUUG
CUAAUACAGUGCUUGUUGCACACAACAGUUGAGUUUACUACAUAAGGAAGGAAUGACCUAUGCAAACAGUAGAUAGAGUGUUCAUAGGGAGAACACA
ACAGUGACAUAAAUGUGCCCACCCAGCUGUAGACAAAGGUUUAUCGAAUAGUUGUAUUGUAGCCCUAUGCCAUUAAAUAAAGUUAUUUACUCUGAAC
AUAAUCCACGAGUAAGCUUAAUUCCUUUGUAUUAAUGUAUUAGGCACAGCAGUGCUUUUAUUGGUGUGAAGGAUAAACAGGAGAUCAGACUUGUAGU
GUUUUUCAGGGAUCUUUGAUCCACGAGUCAUCGCACUGUAAAUAGAGAAGUUUUGUUUUUUAACCAUUUAUACAGAACACUUGGUUGAAAUAUAAAA
AUACAGUAAAGAAUGAACAAUAUUGAAAGGAAGGAAAAAGCUACAUGUGGUAGCUACUACAUGAGAAUGGUCCAUUUGGCUUUAAGUAUUCUUAGAG
UUUAUAUCAUCAGAGAUGUUCUAUCAACUUCAUUACACACCAUGAAUAAAACAGGGUAAAUUGUCAAAAGACUGUUCUCAGUCAUUUGGGGCCCCAG
ACCUGGAUGCAGUAUUCCAGGUGGGGUUUCAGAAGGGCAAUAUAGAGGGGGACAAUUACCCACCCUUUCCCUGCUGGCCUCCCCCUCUUUUUAUGCAG
CCCAGGAUACAGCAGGCUUUCUGGGCUGCAAGCACACACUGCUGGCUCACAUUAAGUUUUUCACCUACCAAGACCCCUGAGACCUUCUCAGCAGGGC
UACUCUAAAGGAUUUCUUCUCCCAGUCUUUACACAUACCUGGGGUUGCCUCAACACAAGUGCAACACCUUGCACUUGGCUUUGUUGAAUCUGAUUAA
GUUUACGUGAGCCUACACAAGUACACUGAGAAUAACCGACCUGACUAUUAAAGACAGAAGCGAAGAAGGCAGUGAGAAUUUCAGCUGUUUCCUUGUC
UUUCCUUGUCCUCAGUGAUAAUGUUUCCCACCAUUCGUUAAUUCAGUGAUGGAUGGCUGCAUGCCAAAUAAUGUAGCCAAAUAAAAAUAUACCUUUA
AAUACAGGUUUAUGGUCAUCUAUAGGAGACUUAUAAAACUAUAUUAGAGAAUUAGGUAUGAAAGAGGCAGGCUGUGAGGAUGCCUUUGAAAAUCCAG
AUACGAUGAAAUUUUCAGCAGGAAUGAGAGGUCUCAUCCUGCAACAAGCCCCAGCACACCAGUAUGGUAUUUUGGUAUCCAUACUAAACUCCCUGGU
GGCCUC..... (truncated)

>*SOX2*, NM_205188
GACAAAGGGGCCGGGGGGGGCGGGGGUGGGGGGGGGGAAGGAGAACUAAACAAAACAAAACAAACAACAACAACAAAAAACUUUUAUUAGAAAUUCU
GGACCUUUUUUUUUUCUUUUUUUUCUUUUUUUUUUUUGGACUAUUUUUGUACAGAGAAAACGGGGGGGGGGGGGGGG

>*KIT*, NM_204361
GUGGCAUCUGGAAGAAGAGGAGCCCAGCUGUACUUGCUGUUUGUAUUGUGUAGGGGGUGAGAGAGGGAGGACUUCUAUUUCUUUUACUUUUACUCAC
UACUACCACUGUGUAGCUGAAAUGUUGUUGUAAUACUGUCCUUUACCACACACUGUUACACAUAAACUUAAUGUGCUGAGCACGGUUACAGGCAUCA
UUGCAAUGUUAACUGAAGCUGUAUAUAUUUUGCUAUAUUGUGUGUAUUUGCAGUAGAGAGCCAGAUCUGAAGAAGAAGAACAAAAAAACACAAAAGA
AACCCUGGGCCAGGGUGUUGAAUGAGAUGACUACAGAUUGAACCAAAUCUGCAGCGGUCCUUCUCUGGAUCCAUGCUUGGGAAGCGUGUAGUCAUUU
UCCAGUUAAUUUUGUUUUGUUGAAUUUAAGAAAUAAACAGAUAUAAAGCAAAGUGCUUUGCAAACCCCUUUCCAUAUCCAGCCACACCUGAGAUGCU
UUCCCCGGCAGACACUGCAGGUUGGAGGCCAGGAGGGACAGACAGUGCGUCCGUGGGCAAGAAGUGGAAGCCAAGGGAUGGACAUGGGCAAUACAAC
AGAGAACUUCCAGACACUCUGGGGCAUGAGGCAGAUCUGGAAGGGAAAGACCUCAGUCUUUAUUUCCUACCUGGGCAGUCAGCAGUGCAGAGCAGUC
AGUAGGUUUCUGUGGGUGCCUGCACCACAUCCCACUGGCAUUCCACCACGGAGCAUCCACCGCAGUCAGAAUCGCAGGGAUGAGAUGCUUGGUUUGC
UAAGAGUCAUGUCCAAAAGCAUUCACCAAAGAUAGGUGGGCUCUGCUGAGCUGGUCAAAUGACAGGAGGAGGGUUGCUUACAGUAAAUGGAGUGUCA
GAGGAGCAGAGGCCGUUUAACGAGACAUCUUAUCUUGGCAGGAGCAGAAGCAAAAAAUUCUAUCCUGAGGGCCCUUUGCUUAUCUUAUGAUAUGCAG
GUGAAUAUUUCCCACAGGCUGGCUUGGAUAAUGCUCUCAGGAAAGGGCUCUUCCUCCCACAAGUCAUUUCAUCUUUCUAUCUCUGUAGUGGUGCUGA
GAUCCCAGAGAAGAACUUCACUGGGAAGCAUCGAAUUUUUUAAUGGAAUAGAACAAUGUAUACCUUCGGUCUUGUUUGGAGGCAGAUAUGAGGCUGU
GGGGUCAGUUUCUGUUGGGCCUGGGAUUGUGCUUAAGUAUCUUAUAAUUCCUUUUCUUCCUGUUGGAAACACUGUUGGUUCUUUUUUUUUUUUUUUU
UUUAACCCUUGUUCUGUCGCCUUUAAAUUAGUAAAUGCGUCAAGAGGAGCAUUUUUCAGACAGAGAGUACCAGUUAAGUACCUAUUUAUUUACAUUG
CACUUAAGCACUCUGUAACUUAUGUUUUACCAGGAUUUUGGUUGAGUUUACAUGCAUUUUAAAUGUGUAACUAACUGCCCAAAAUGCAAGACGAUUG
UAAAUACACACACUGGUAGUACAUUUUAGGUAGUGUUGAAUACCACACUCUUUAUAUGCCUGUGCAUUUGGUUUAUUGUUACAGGAAUUCAUCUUUC
AGAGUUAGUCGAGAAGUAGCCAUUUGCACUGAACUUACUUAGGCUGUUGCACCUUUCCAAAGUUAGCCAGUUGUUUGGAGGCACUCUUAUGCAUACU
UGUUGUUGAGCAUUUUCAGUUUAAUGCUAUAAAAGCUCUUUCGUAGUGUGUUGGCUUUCAGAGCAACUGUAGACAAACUAGUUUUAUAAUCAUAGAU
GUCUUGGUACUUUAUGGACAUUUAGCUGAAAAAAGUUUGGGUUUUUUCUUUCUUAAAAUUUAUAUUUUUAUAAUUUGGGGGGGGUUGAAACUUAUUU
UGCAAUGGCUUAGUGUUUGAAUGGUUUUAUGCAUUGUUUUUGUAAAUAUGGAAAUGUAGCAAUAAUGUCCUUUUGACUAUUCUCAGUCCUUGAGUCU
CAAAAAUAUUUAUAUAUAUAUAUAUAUGCAAAAACUAUAUGUAUAAAUAUGUAAGUGUUCAAAAGUUUAUGAAACACUUAUGGAUAUGUUGCUCGGU
UGUAGAAUUGCAGUUCAGAAGAGUUCAAAUAAAUCUGUAAAUAUGUAUUCAGAUGCUACCGAUGUGUACUAUGUUAAGAUGGAAUAUUUUCAUGUAA
GUCUUAAUAAAUUCUUGGGUUGAAGC

>*GPR125*, XM_420763
CAUUAUCUUCAGUGUGGGACAAAUCCAUAUGAACUGUGAUUACACAUUCCUUAAAGCUAUUAAAUGCUUCUAAAGACUGUUUACAAAUUGUAGGUAC
UUUAUGCAGGGAUGGAACAGAGAUAAUCUGCCAAGCAAAAGAUUGUAUGCAUUAUUAUCUUUAUUCUUCCUACAAGGAUGAACUUUUGGUAUUUUGU
GAUUUGUGUUUUGGGGUUUUUGUUGUUGUUGUUGUUUUCUUUUGAAGCCUCAAUCUUCACAGAACUGUAGAAUAACAAAACUGAGUUGGAAGGGACC
UCUGGAGAUCAGCAAGUCUAGCCCCACUGCUAUAGCAGGUUCCGCACAGUAGGUUACAGGUAGGUUAGGGGAACAGUUCCUUCCGUGUGAAACUACG

CUAUUUCCACAUAUCUAGUUACAUUAAGCAGUAUAUUCCCACUUACUUCUCUUAGCAGAUGGAUGUGAAAGAUGUCUAAUAAAAAAAAUAAUGAAUU
UGACUACUGUCUACAGUACAGAUAGAGUACUUAGGAUGGAAAAAAGUGACAGGGAAAAGGAACGUAGACAGCACAGGCUUCAAAUAUAAAAGAUAAA
GCCUGUUAAAUUAGGUUCAAAAAAUUUUAAGAGUUGUUACAUAUGUAUUUUUUCCAAAGUGAUGACUUUUGCAUGUACUUUGUAUGCAACAGCAGUC
UGAUGCAUUAAAAAAAAAUCUGUACAGGCACAGAAGACAGAAACAGAACGGAUAAAACUUUGCAACUUGUCUUUUUUGCAAGUUUGCUUAACCGACU
AACUCUGUUUAAUUAAAUUCUGACACAUCUAAUCCUCAUUCUGUCUUUAGUAAAAGAGUAGCUGAUAAACUAAAAGGGUCAAAUUAGCUCCAAUAGC
GGUAGAAAAGGUUCCUCAAGAUUGAGCCAAAUUAGCACAGCAAUCACUUCUUUGAAUAGAGUACUCAUGUCUUCUGUUUCUGUAUCCCCUUACCAAU
GACUGGAUUUUUACUGCAUUUUUCAGCCUUCGCCUUUCUUCCUCUUGCUUUUGCCUCUCCUGAGUGCGAUGCAGGAGAGAAGCCUUUUUUUCAUUCC
UGCUCCCUCCUCUGAGAGACACCUUGAGCCUUCUUUUGAAAGCCCCCUCAAAGCUAAACAUGUUUACAGUUCAUCCCAUCCAGAGCUGAGCCCAGAC
CCUGAAGCUGACACCAUCAGGGGUGUCCCGGUUGGCCAACCAAGGGCUACAUGGAGGGUCCGCCUCCACCUCGGUGGCUUCGCUUCCAUGGUCACUG
UCCUUUCCGUCUGGUGGCUGAGUGUAGUUGGAAGGAGCAAAGGAGGGGCUGGCGGGGCUGCUGAUGUGCACUGGGGGAGCAGCCAGCUGCAUCUUGC
UGAGUGAUGCGGAACACAACUUUGUACUUUUUUAUUUUUCACAUGUAACUCUGAAGAUGUGUCCACAACGCAUUUCAAGUUUGUAUUUAAAUGUAUA
AUAAAUAGUAUUUUCCCCAGCA

>*SYCP2*, XM_015296671
AGUAGUACUUCCAUAUACUUUCCUGAAUUUCAAGCUCAAUAUAAACACUCAUUUUUAAUACAAAAUUUGACUUUUUUUUUUACUUAAGGAAAGUACU
GUAAUGAAAGCAUAUUUUCCUUAAUAAAAUCGAAGUCUUUUGACUAAGACUUCUCCAUAAGACUUCUAUUACUAGAAAAAUUUGGGAUUUUACUGAA
UUUACUAAAAUAAAAUACACAUUUCUUUUAUUUUUAGCUAAUGCUAUUUUUGAGGAAGUCUUCAUUAACAUUUUUCAUAGGUUAAUUAACUGAACUU
UUAAAGAAAUCACUUCAAAUUUCACUUCAAAUCUAUCUCUUGGGUCGUUGUGUACAGAAUACCACCUUUCAUCUUUUUUCUGAUCCAGAAUGCAGUA
AGAAAUUAUUUAGGGAUCCAGUCUCAUGUGCCAAUCUCAAUCCUGUUCAUCUGUGGCAUUAGACUUCAGUAAGAGUUUCUAACAGAAAAUGACUGUA
AAAUUUUGUGGUUGAAUGAUAUUGCAAAUAUUUAUAUUAAAUAUUU

>*ZP2*, NM_001039098
CGCAUUUGUUAACGAACGUGUAUUAAAAAUGUGGCUUUUCAGGCAAGAGAUG

>*GRTP1*, XM_416941
CUCAGCAGCAGGAACCAGAAAUGGACACAUUCCUCUUUUUGCACUAAAGCACACUUUAAGCUUUCCAUGAGUUAACUGACACUUGACCAAUUUUUUC
CUGCCUUCCAAGUAAGUGUUUUUAACGCUUUGUACUGUAUGUCAGACUUGAUAACUGGAACUACUGCAACUGGUGGAUUUGUCAUUAUUAUUUUUUA
AGAAUUAAAGUGUUUUCAGAGUAGAAUAUGAUCAACGUAAGGACACUCCUCUUGAAGUCACUGUCCUUGUCUUUUCAGGGUGUUUUGUAAUGUUGAA
ACAGGUGUAUAGAAUUACAUAUCCUUCGUAUUGUAUUAACUCUGGCACCUCUUACCAUUUGUACAAAAUAAAUCUAAUUUAAUUUGUCAGA

>*GFRA1*, NM_205102
CUGCAUUGCAAAAGGACAUGUAAAAAAAUCUGUUUCCUGUCCUCUGUUGUUUAUCUGGAAUUCCAGGUCUGGGGGCUAAGCUGAGGCACUUCUGCUA
GAACAGUUUGUCAGCUGGAAAAAUUUUUUCUCUCUAAAAAAGCUUCUUGUGAUAUUUAGAGGCUUUGUGAAUACUUGGUGCAGUGCUACAUUCCAAA
CCCAAGAGGCUUUUGGGCAUGCAGUGUUUUGAAGAGACAGUGUAUAAAUUUGCCUGUAAAGAGAUCUGGUUGGAUUAUUUUAAUAAUUAUAUUAAUU
CUGGCCUUUACCUGAGAAGGAGGAUGGCAGUUUUCUUAAGAUCCUAUUUAUCUCAUUGGAUGGUUUUGGUUUUCAAAUUGAUCAAACUCCAGAUUAU
CAAGGAUGUCAGGCUUUUGUCUAAUGGUGAAUGUUCUCCCAGAGAGUGGACUUUAUGAAACUUCUUCAUUUGAUAAAUUGCUACUGAUGUUAAACUC
UUUCAGUGUAUUAGCAUUUUCCUCUUUAAAUGUUUACGUACUGUAAGUGAUUCUGCGUUCCCUGCUGAGAAGCCAUUCUAAUUCACAUACAGGUGUA
ACGUAUGUCUUUCAGUUAAAUUCUUAUAGAGUGUGGCAUAGAACUUUCUAACAAAACAUUUAUCUUUUAAUUAUAAUCAUCUAGCCUUAACGAGGGU
GAAGAUUCUUUAAAUUAACAAGAAGCAGCCAUUGUGAAAGCUCCGUAAACGUACAUUUCAUAAACUUUGAGGAUGAACAGUAGAAAAAAAUUUUGCU
GCAGGGUUUCAGCUGUACAGUCACCGGUCUUCUGUGCUUCCAUUGUGACAAUAUAGUCCAAUUAUUAUACUGUUCAUUCAAUAAAAAAGAUAUGCAU
UUUACUUGCUCUACU

>*HHIPL1*, XM_421358
UGGGUGCUGCAGAAGACAGCUGAGGGACAGAGAGCAUGUCCCCUUCCUAAUCGACCCUCUAAAGCAGUGUAUGUAUUGAGGAAGGCUUUCUCUGCCU
AGGGGAUGCUGCAUCUAUUUGAAGUAAUCUCGGCAUUUUCUUCAUUCACUUGCAUCUGCCUUGGAGGGAAAGAAAAAGUAAAAUUCAGCAGUAACUU
GAGCGUGUCCCGGCCAACGGAUGGGUUUGUAUGGGGUCUUUUACAAGAAGAAAACCCAAACCUCCUGAAUUACAAAUAAGAAAAAAGAUGCGUACAG
UUGCCCUUUCACCAUACAAAAUGCAGCAUUCCACCACCACACUUCACAAUGUUUGGCUGAAAAAGAAAUUCCCAAAUGGCUGUGUUUCAGUCUUUCU
UAUGCAGGUGUUUGCACUGUUUUCCUUGCUUUGCUGGUUGUAGCUCUCAGUGGUGGUUUUGAAGUAUGCAUCAAUCGGUGCCUUUCUUUGCAGAAUA
CAGAAGUAGACAUUUUGGCUAACUUUACACACUAGUUGAAAGGAAAAAGAUUCUCUUGUAAUGCUUAACAUUGGUGUAAGUCUAUGGAUUAUCAAAG
AUAACUGCAUCCAAGCAACAGUCAGGUAAAUUACUAAAAGUUAGGGCAUGCUGCCUAGAAAACCCUUCUUCGAGGUAUGCUUGCCUGAGAGAAAAGA
AAAUCAGGUCUUUUACAUGAACAGAAACAUUAUCCACUUAAACACAGAAGAUACUUUGUACAUGUUCUCCGUUUCAUCAAGCAUUGUGAAACCACAU
AUUUUUGCCUUCAGUUUUCACAGCUGUGAAGGAUGUAACAUGCUGGGGUAAUAGCCCUGUAAUUAUUUACCCCUUUUUAUAAAAAAUAUUAAAUCAC
AACAACCUGCAUUUGUCUGAAA

>*UNK*, XM_015295331
CCUCCCGAGGACGUUCAUUCUAAUCAUAUCAUAGAGAACUUUUUAACUUUAUACGUACGUACAUAUAUAUGUAUGUGUACAUAUAUAUAUGUAUGUG
UAGAUAUAUAUAUGUAUAUACGUGUGUGUACGUGCGUGGCUGUGUAUGUGUAUGGAUAUGCACACACACGCACAUACCCACACAAAACAGAAAUUAG
UUGCAGAGCACUUUGUAAUAUUUUACAUCCCGUAUCUAAACUGCCCCCCACCCGUGCCCCACUAAUUCUAACUCUUGAGGAACUUUGAGAGCUGUUU
UUAAUACAGAUCAAAGGGCCGUUUGCAAAGAGUCUGCGUUCCUCCUCUUUUCUAUUUAGGUGAUAACCAAUUUUUGACUAUUUCCAGAAGGAUUUAG
AGUGGGCGAGAGGGGCUUCCACGUGCUUUCCAGAGGAAGAGUUGAGAUCAUGGGGGAGGAUCGAGGUGGAUUGAAAUGGCUGUUUCACGUCCUUGGU
CUCCCUGCUCUUUCACUGACCCAGCCUGGUUAAAGCCGUCCUCCCCGAUGGGAGCUCUGCUGGGUCCCUGCCUGGUGGGCCUCACGUUGCUGAUCUC
UGAAUGUGCAGAAACUUUGUCUGUCGUUUGAAACAGCAAAGUUCACUUUUUAAAGUUUUUUACACUCUUUGUAUAUUUAUAUGAAAAGAAGAAAAGG
GGGGAAAAAAUAUAUAUAUAUAUAUAUUGUAGCUAAAGCAAGCCAGGCCCCCCCAAAUUCAGUGACCAAUAAUCAUUGCUGAUACAGCUAACACGUU
GCAGGUUUGAGCAGCCUUUGGGUCAUCAUCCUUGCGUCAGCCGAAGCCCUUUGUGAAAGGGCAAUGGGUGCACAGGUCAGUUCUCUGCACACACCCC
UUCAUUGGGUGGCUGCCUGAAGAAUUCGUACCGUUUCAGACCAGAUUGGUUUUCUUUUAGCCACAGAGAUCUGUUUUGUAGUCACUGCUGGAUCACG
UGAAACAAUUGCAGACACAGCCUAAGGACUCUCUGACUUUUUGGACGGAGUUGGCAUUCCCUGUUUCAGCAGCUCAGGGGUGGGCUGUGGGCUGCUA
AGUGCAGAAGCCCUCAUGCCAGGGAUGGUGUCAACCUGUGGUGACAGAGAAAGCCCCGCAUGGAACAAACUGCUGAUGAACCAAUUGCAGCUGGAGG
CUGGAGCUGGAGGGGAGCGUACAGUCCUUGCUCUCUGCUGCUCCUAGGUAGGGAUGCUUCUUCCUGCUCAGCACUGCGACACAAAACGCAGUGCGUG
UUACCACUUCAGCUUAUAAACACAGCCACUGUUUGUACGUGUAUAUGAAAGCAUUGUUGAUGGGGAAACUCUAAAGCUGGUUGGUCUCAUGAUGCCC
UGUGGCAGUGAUUCCCACAUCAGUUAUGUUACAGAGAGGCUAGAAAGGAAGAGGGCUUGAAUUGCAGAUUCUCAUUUAGAGGUUCGUAACUCAUCAG
CUGCUCUCUGCGGUGCUGUGAGUGGUAGCGGCGCGGCGGUUGCUGUGUGUGGCUGUGUGUUGGGUGCAGGCUGAGGGGCUGAACCAGCCAGGGCAGA

GCAGAAGGAAGCAUCUCCAUCAGAGGAAGGUGACUGCGGGGCUCUGCGACCCUCUCGCUCUCUUUCCUCCCAUCUCGGCCCCCAUUGCUGAGCACAG
GGCUUCCCACCGCCCUCCCGUCGCUCUCACUGGUGUCUCUGACCUGCUGAGUUUGUUUCGGGCCGUUGUCCCCAAACACCGCCUGCAAAAAGAGCUC
AGGGCCUCUCUCCUCGUUAGGAGCCUCUUUGUAUUUGAAUUUAUCCUCUCGGCUCAUUUCAGAAAGGCAAAGGAGGAAAUCCCACAGGCUCAUCCAA
CGCGAGCCGCGCCGCGCCGUGCGCUUCUCUUGAAGGAAAAGAGAUGCCUCCAGCUCAGACCUGCCAGCAGCUGAGCUUUCCCAUCGCAUCCUUUACC
AGCACAUCCAUCGCGCUUUCCCGUCGGGUGGAUUGCAGUAACCUGCUGGGGGCUCAACCAGGAGGCCGCUCCACUCUCUCAGCACUGCUGCUCUGUG
CAGGGAUGCUGCCCGCGCUGCCCUCCCACCCUGGGAAAGCACUGGAACCUCCAGCAGCCCACUGUUGUUGUAGGGACACCCAAACCACCACCAGAGC
CUCUCAGAAAGCUUCCUAUUGUAACACGAAGACGUAAUUAGGUUCGCUUCCAUAAUUCCACCUUUCCUUUGUACAGGAAGGGUUAACACGCAUUCCU
GGACGAUCUCUUAAGUACAGCUCUGUGUAGUUCUCUUGUUUUGUUUUUCCCAUUAACGUGU..... (truncated)

>*SALL4*, NM_001080872
GAACUCGAGUGAAGAUGACGACAGAAAGAACAAAUAUAUAUAUACAAACAUAUAUUUUAAGAGAUUCCACUUCAGCCUCCUAUUUUCCUAUUGACGU
GCAAAUGAUUUUAUGGUUGUCUUUGUAAGAUUUCGUUGCCCAGAGUACAAUCAUGGUAUUGCUUUGCAAAUAGUAAAUAAUAAAGAAUAGGAUUUCC
UUUUAUUUGGAAAGAUGCGGGUCUUGCAAAGUAGUUCUUACGUGUGACUUUAAUGUGUACAGCCUUACAGAAGUUUCUACAACUUGAAAUUCCAAAG
GUUUAGAUUAUUUACCUCUUCAUUUAGAGUGAAAUACUUGGGGGUUUUGAAUAGAGUGGAAACAGCCUACUGUUGGUGGAGGAGCUUGUAUUUACUG
AUGAGAAAUGAAAGCUGUUAAUGCGGGUAAAUAUCCUGGAAUGUCUGCCACCCUCUUAGAAGUAGUUUUUCUGUUUGAAUUUUUGGCUCUGUGUGAU
UUCUGAAUGUACUUUUUUACUAUAAUGGCGAUAAAAAUGACUUUUUUUUUAAUUCCUUUUUGCCUCAGAAGUUCUAUGAAAGGAAGUUGCUGUUUUU
CUUCAAUCACUGCUUCUAAGGAAUUCUGUUUUGUUGACUGCUGCUUAUUUAAUACUACUACUAGAACAGUCCUCCAAGUGUAGUGCCAAAAUCCCUA
CUUCCAAAGAUGUGCAGUUUUUCCUAGAUGUUUUAUUUCAGUACCAAGAGCCCAAAACAAACGUGGGUGGGUAUGUAUAUACUUUUUUCUUUCUUGA
AGGGAAGACUUGCCUUGGGAAGUCAGGUCUAAAAGCUCUGCAAGGAAUCUCAGGUGACUGUUGUAAUUGGGCAUGGAAACUGCUUUCCUUAGCAAGA
GACUUUGUUGGUUUUUGUUUUUUUAGUUUUAUUGUUUUUAAUUUGAGUAAUUUAGAAGAUCAGUCUGUGUAAACCUGUAUUUAAAUAUGAAGUAUUC
AUGCUUAGGUUAAAUGUUUUUUGGGUUUUGGAUGGCAUUCCUACUCUGGUCAUUAAGUUUCUUGGUGGUCUGUGAAGUUACAUUCUGCCAAAAAGGU
AAUUAAUGAUCCGUGCGUAUUGUAUGAAGGUUGCUGGGAUGGUCAUCUGGAUUCUAAGUUAUCUACCUCAUUUUUUUUUCUUUUUGUAGAUGUAGUG
UCUAUUUUUCUAUUAAUGACCACUGUAAUAAACUGAGAUUCAAGCAGAUGCUUCCAUGCACUAUCUGAAACCAAAACCUGAUGUAUUAGUGGAAGCA
CCUGAAGACUUACUGGACUGACCUUUCACUAUUCCUCUGAUGUGUGCUGGAACUGUCGUGUAAUGGGAUCUAAAGCAGACUGGAAAUAGCGUUUGGU
AAAGAAGGACUGAGUGAUGGGAAGAAGUCUUUGUGGACCGCAGGAAAUCAAGUCCAGUCUCUGUUGUGAAAGGCUCCAAAAGUACAGAUUACCAAAA
GUUUGUGUAAUGUUGUUGCACCUGACUCUUGCUUAUGAUCUCUGUAUGCCGAGUUAUGCCUUUCCUGCUGGACUUGUAAGUGAGAACAUACCAGUAC
CUGUUUACACUGUAAGAUGGAUAUUGUUACAUGGAACAUGCAAAAGGCAUCCCUGUUGUCAAGUUUUCUGCAUUGUGAAUGUAUGACUUUUAUGUAA
GAACUGUAGUUUUUGAGUAUCUACUGACAAGUUUCUGUUGACAUUUUGAGAAUAAAUUUUGGGAUGGCUUUUUCACAAAAAAAAAAAAAAAA

>*GATA4*, NM_001293106
ACUGGUCUUCCACCCAUAGACUUGAGGCUGAUCCACUGGAGAGAUGAAGAAGAUGCCACGUAAUAAUCAUGUCGAUGUUGUUUCCCUCCUCCCCUGC
UCUCCUGCCAUGUUCCAACAUGUUGGAUGUUCCAACAUCCGAGAUGCUCCAACAAAGAGGUAAAGAGGACUUACGUGAAGUGUAGGAGAGCUCUUAC
CAAACAAACCAAGAAGACUCAAAUCUCAAAGUAACUAAUGGAUUUGAAACAUUUUUCCUCCCUUCAAACAAGUUUCCUUGUAUUUGAUUGUCACUAC
CUUCUAAGAAAUAGCCAGAAACACAAAGAUGAAUAUAUUUUUUUUGGGGGGGGGGAUGUCUAUUUAAGAACAUUUGCCAUUUGAGACUUAGACAGUG
GUGGCACAGACACAUCUUUUUCUCCUGUCACCAUCCAUGUGUUUCCUGACCAGAGGGAAUGCCAUCAUCAUGACACCGAUUCAGCGACCUUCCACCA
AACAGAGCCAGUUUCAGAACAGUUUGUUGAACAGACUUCAUGAGACAUGCUGUGGUGGCUUUAGUGGAUUUUAUAUGAUCUAAGGCAGAGAAAAAAA
GAGACCCACGUCCUCAUCCUCAGCUCAUGCAUUGAGAGCAUUGCCUUCAUUUCCCUAGAUGGUCUGAAAACCUAAUGAAUGUCUGUAUUUAAUAGUA
ACUCCUUUCAUAUUUAUCACUACAAAAUCAUGGGACUUUGUUUCCAAAAGAUGGUUGGCUGAGCCUGGAGAAAGUCUGUCUGAAGUCAAAGAGGGAU
UUUGGAAGGGGAAACUUUCUGGAAGUGGGUAGAGGAAACCUACCUUGGUCAUCAAAUCCUGGAGGAAACUGAUGGGCUUUUCCCUUACAAAUAGCUA
CAAGCCAGUCUUAGAGAUUAUCGUAGAGCACAGCAAGUGAAAAGGUGCUCUUUAACACUGUUGUGCUGUUGCUUUUGAGGCAGUUAAUUUAAUUAUU
UUGCAUACUUUUCUGUUGUCUUCAUUGUCAUUGAUGACAUCUGAUUGUCAUUUAUUUCUCGUUGUACUGUGAGUGCCCACUGCCCCCCUAGCAGCUG
GCUCAUGACCAUUUCCCAUGUCUAACCCUUAAGGAUUUCACCUUUCACAACAAGAAAAGUCCUCAGCCUCUAAUGGAUGCAGACAGGGGCCAUCUUC
UUUACUAAGCAUGUGCAUAGACUUGCUUGCCUGCAAGAGAAGCUUAACUCCAUUCAUACACGAGUAUUAAACCAAACUCAUGUGUUUGCUUAACCAG
AAGUCUCUUAGCAACAAAAGAAUUCUGGAAAAUUGAGUUGAGAGUGAUGCUGAGAGGUCACAGACUCAUUCCCUUGUCCAAAAGGGAUCAGUUCUAC
CUAACCUACCUUUAGUUGCUCUUUGUCUAGCAUGUGCUUAACAAAAUCCAGCAACGCAUAAGCUACAGCUCCUCUGUGCAGUCUAAACCCUUCCACA
GAUGCUUGCCAGGAUGUUUGCAUCACCAUUCCCAAGGAGGGACAUCUUUGCCUGUACAGGUAGAGAAUUUCUGGGACGCCUCAUCCUACAAGAUUCU
CCAGUCUUGUUUCUGUCUGCUCACAAACUCCAUUACCCUUCUUCUUGCCUUCCCAGCUCCUUCUUUUCCUCCCUGCAUCCAGAACUUGACUAUGGAA
CUGCCAGGUGAUACCAGAAGAGGGAUGGUAUGUCCCUACAAAAUAUGCUGAAGGAUAUAAGGUGCCACCCUCCAUCUUACACUCACUCUUCACCAUU
CCCACAAACAGUGUUUCUGACAACAUACCAGGAUAUGUGGCUUCGUGAAGAGGAAAGCCACAAACAUGGUUGAAGCAAAACCAAGACCUUGUAUUAU
CUGUGUUUGAACCCUCUAUUUUCCUCCUGAUGGUUGAAAAGUUUCUUUAAGGAGAAGGAUUCAACUGGACUGGUCAUGACAGAGAGUUGAAAUGGAG
CACAGUAGAGAGCUGGACCCCUCCACAACCCCUGUGCUCUGCUCAGUAACUCAGAUUUGCAGGGGUUUGUAGGGGUGACAGAGACUCUGCGCUACCU
GGAGAAAGACACCAUGGCAGUGAUGCCACGGGGAUGUCUGGAUGGUGUUUGCACCUUGAGGCUCCUCACUGAGCUUCCCCAAUGUCCUCCCAUUGAC
AAGGAGUGCCUUUCAGCUGGGUUAUGGGAAUGAAACUCCUGAUCUGGGAAGUUUUUCCAUCUGCCACUUGCAAGAUAACCACAGAAUUUGCACUCUG
UCCUUCAGAGCUAAUGUAAGGUCUAACCUCAUUAGUUUAUUAACUACAGACAAGGAAGAGACGUUCAAUGAAACACAAAUAAAACGUUACCCUGAAA
UCCCGUGAU..... (truncated)

>*GRIP2*, XM_025154652
GCAGCCGCAGCGGACGAUGCCUGGGGCCUCUGCUGAGCCAUUGUGGUAUUUGGGGUGUUCUUCCUGUUUUUUUUGCACUGGUAUUUGUAAACGCUGU
GUGUAUCAUCAGCUCCAAGGGGGGGAGGUGGGUCUUCAGUGAGCUGUCACCUGUAGCCCACCCAACGCUGCUGGGAAGGAUGAAGGUGGAUGUGCAU
UUCUUGGGCUGAGGUGAGCUGUUGCAUCCCCAGUGCAUUGGGGUGCACCGGGUCCUGCCCCAGCACCUCCUCGCUCUUCAGGCCUUGGCCAUCCUCC
AAACACACAUGGUGCUAUUGUUAUCCUUGUUUGCUCUGUUUAUCACAUGCAGCAAGAUUGAAAUGCCAUUCCUUUUCUGUUGUACCCUGUUCCACCC
UCCAAACACUCCUGAUAAAUUGAUUCCUGCAGUCCCUUUCUCUCCUGCUGUCAGGCUAUGGAGGGCCCUGUUUGAAAGACAGUUCAAGAUGUACUUG
AUGGUGUCUAGGUGCAGUUCUUGUUCAGAAAUUCCAUGCUGCCCCAGCAGGGCUGGCUGAGCCCCGUGCCCACUACCCAGGGCACCUCGUCUCGCCA
UCAGCAGUGUGGCUGUCUGUGCUAUAGAGCAGCUGUGACAGAGCAGAAGGCUGUGCCCGAGGCUGAAGACCCUAAAUGCCUGCUCGAUUCCCUAGCU
GUUGUUUUUCAGAUGGAGAAUAUGGUUUUUAUGGAGAUAAUGCAAGUAAAAAGCUGAAAUGUUGUUAUCCAGGGUGGCUGAUGUUUUGAAUUAAUAG
AACCCUGUUGUCCCCCACCAGGCAGUGACAUGAGAAGCCCCUUGUUAGAGGUUGUGGGUCCCCUGUUCCCACCUGCACUAGCAGUGCCCAGGUGGCU
UUGCUGUGUCAACCCCAUUGCCUGUUGUUGGCUUUCCCAUGGCUUUCCAAGCAUGGUGCCCCUGCUCGUCCCUCCCACCCCUGUGUAUCCCCAGGCC
AUGGGGAGGAGGCAGUUGAGGUAUCAUCUUCCCCAUCAGCUGGAGACACAGUCAAAAUGUGGCUGUGGGCUCCUCCUCCUCUCUUUGCCAUGAGUUC
AUCCUGUGGCAGAUGUGAGCUGCACUCUGUGUUCCAUGUCUUAGCACUUUAUGAUGAUCUUAGCAUCCUGGCAUUGCUUUGCCUGUGAAGGCUCAGG
CGUGGUGGAGGUGGUGUGGAGGUUGGGGGCUGCAGCCCUGGUGACCACUGCCCCCACUCACCAUUUCAUUUCCUCUAUCACCACCCCCUACCAGUGC
UGUGUUUAGGUGCCAUAGGGCUGUACGCAGCAUGUUCCUCAUUUUGCUGAGCCAAGACUCACUCCUGCAUCCAUCCCCACUUUGCAGCUCUGUCCCU

CCACGAUAGCGCAGCGUAUUGGGGUGUUUCUUUGAGGCCUGUCCAGCCCCAUCCAGCUACUUGUCGGUGUAGCCAUUUUCUCUUCGAGUAUGCAAUA
UCCCAUACCAUUGCUGCCACAACUAGAUAAUGUGCCAUAACUGAUACUGUGGAGCAUUGACAGUGCAGCCGAAAGUGUUUUUAUAUGCAAUAUUCUG
AACAGUGGAAGCUCGCUGGGGCUUCUCAUAGGUUUUUAAUGAAGA

>*EEF1E1*, XM_418953
GGAGAAUUUUAAUGCUGUGUCAGAGUGGUAUUUGGGAGUCUAGAAGUUACAUUGUCCAAAACCACUGCCUUGCAAAUACUGGUUUGUAACCUUCCAU
AUGUGAACCAAAACUGGUGAUGCCUCAAGUAAUACAGUUCCAUUGAAUUGUUAUUGUUUGUUCAAGAAUUUGAACUAGGUACAGAAUUUUAUCAUGU
AUCUGAUUAAAUGCAACAAUGACAGAAAGAUGGGAAUUGAGUUUUAAUUCACUAUUUUUUAAAGAUGAAAUGUUCUUUUAGUUAAGCAUUCAACCGU
AAUUACGUGGUCUUUGUAGGAGAGAAUUACGUAUUUUCCUUUGACUCCACACUCAUUUUACUAUUUAUUGAGUUUCUGCGUACGUCCCUCUUUUCAU
UUUAUUUUCCAUGAAUGUUUAUGCUAGUUUUGUAAAAGUUAAGAGCUAUAUUGUGUGCCAAUGAUGUGAAAAAAAGGGAACAUAAAAAUGUUACAAA
GUAUUUAUGUGACUGAAUAAAUUAUUUUAAAACUGUAGUCUUAA

>*TEKT1*, XM_004946828
AGCCAUAGUUUGUGCUGAGAUUUUUAUUGACAAACUCUAAAUUUAUUGA

>*TRIM36*, XM_015280829
GCUACUGAUCUCCUGGUGCUGUUCCGAAAAUAAAAACAAGAGCAGAAGCAUUUUACCUUAGUUUUCAGUCCUGAUUCCAAUACAGUAUACCUAAGUG
CUGGUCACACUUCUGUGUUGCUUUUUUUGUUUGUUUGUUUGUUUGUUUGUUUGUUUAAAUGAACAGUCCAGACAUUUCCAACAAAAGGAAAUGUUCU
UCCAGGAACUCUCCUGCCUUUCUUGUGAUCUGUGUGAUGGUGAUCUCCACAAGCUUUCCUCCUAGCGAGGGUGGGGAGGAAGUUUGACUAACAACCA
AAACUACUCUUCAGGGAUACAAAUCUGCCUUUUAUGUAACAUAGAUUCUUAGAUAGUCAGUUUUGAGUUUAACAGACCAAACUGACAGCAUAUGAUU
GCCAUUAUUCUUUGAGAUACCUGUCUUAAUGCAUCUGCAGGAUCAACAUUGCACUAACAGAAUAAAAAGCAGCGAGAAAUGAAAACUGCAGGUGAAC
AUUUAGGCUGUAUUUGGAAGGAGGACGUUAAACUAGAAACUUUAUUUUAUGUAAGUGUAAUGUAACUGUAAGCUGAAACUAGUUCUAACGCCUGAUG
UAUAGUUGAUGGUAUAAUUGUAAGUUCCUGUACUGUUCCAGGCCUUUAGUGUAGACAUAAAAUAUAUAAAGUGCAAUUUGACCCGAAGUGUCAGACU
UCCAAGUCUGAUAUCCGAAGUUUUUGCAUGUGUGAGUAGUAGUACCUGGUUAUAGGCAAAAGUGAAUGAGUACUUGACAUGACAAAUAACAUGCAAA
CUUAAAAAAUUCAUGUAUUUCUGUAAUACAUGUUUUUCUAAGUUAAUUUUUUAAUCUAACAAUGGCUUUCUAGUACGAAUGAUAAAAUGCACUGAUUC
AGUUUUAAGACCCAAUUUCUGCUUCGAAAGGCGUCUCCGUUCAGAUUUCUGUUCCUCUCGUGUUAAAAACUGUAUCAUGUUGUAGUGCUUGCAUCUG
UUUGGAAGAAAUUUACACAAACAGAAAUCAAACGUUUGAGUCACUUCAACUGUUAAUUGUGAAAAAAUAAGGUUCUGAAGCAAAGGACAUUUCUAAA
AUACACUCAAUUUUUUUAUGAAUUUGGGGAUAUAAUGUUGCAUCUUGAUGAAUGUGAAGUAUGUUUACUGAGUACCUGUAUAUAGCUGUAGAGCAAU
GGACUGUGACCUGAAAAUUGCAAGUGGCACCACUGGUGAUGGAUUGACCGUUUGUCUUUAUCUUAGAGGUCUUCUCCAAAUCUUGUGAUUCCUUGAU
CCUCGCAUCUUAGGAGACUGACUCUCUAGCUGUGAAGGACGGUGAAAAGAUUUGGGAUUUUCUUCUUUAUGUAAUCUUAAAACUUAUCUUUUUGGUC
CAUACCAGAUAUUAAACUGAUGAGGACAAAUUUUCUUCUGUAGAAUUUUCCGCGUGAUCCUAAUGGUUAGCUCUGAACAAGUUGCACGCAUGCAAGG
CUUCCCCAUCUGAUGUACUGUUCUGCGUUUAAUAAGAUUGAGUCUCUUCUGCCAUCUUGCCUCAUCACUUGGUACUGAGGGCUUCCUGCAGCUCUCU
GGUCUUUUGAUUGUUUUGAGUAAUUUUAUAGUGAACACAAAUCUUUUCCAAAUCUUGCAGCAGCUUAAGUUAUAGUGAGACUGAGCAGUUUUGUUCC
CACUGUUAAAAAUGUACUGUUCCUCCAAUUUUGUUAUUCGGCCACUGAAAGCCCUUACAUUUUUGUAUGCUCUGCUGACUGGAAGACAACUGGGUGA
CAGCAACGACUUUAUGAACUCAAGUUAUUAAUCAGACUCAGAAGGCAAGAUCCCCUUUUCUUUAGCUAUGGCUUAUCCCAAUGUGUAAUUUUCAUUC
UUCCAUUUAUCUACCAAAUCUAAUAUCAGCUUAAAGCUAGAGAUUGUAGGGUUGGAUUUCCUAAUCUCUUAUCCCAGAUCAAAUCUCGUUCUACAGC
UAAAUAUCUCGUUUUUAAUUCUAUUGUUUCAGAAGUUGUUCUAAAUUAUAGGUGUUAUGUCUCAGUUAGCACUCCAUAGUUCCACACUUGGAUCUUU
GGAAGCAUCUGGACCAAUAUGACCAGGCUUUAGCAUUUCGUUACUUUAUGAAAAUAGUCAUAAAAUAGCCACACCAUCUUAAAUGUGAAGAUAAGUC
AGUCUGAGGGGCUAUCUUUUAAAAGAAGGGGGGAAAAAAAGGUAGUGGUUUAAGAAAACAGAAGUAAAGGAAGAAGAAUUCAGUUUUUUCCUAUUAU
AUCUUUGCCUAACAUUCAUUUAAGAUGUGCUCUGCUAUGCAAUUACUUAUUCGACUGGACACAGGCUGAUACUACUUAAAACAUUUACUUGCCUUUU
GGUAUGUAAGUAUCUCAUUCAGCAUUUUAAAAAUGGUAUGGCUUUUGGUGUAGAAUACCUUGGAUGUGUUCAUCACAGUACUGUGUGGUGCUUGCUC
CAGUUUAUUAAAUAGAAGCUUACUGUCAGAGGCAAAUGAUUUUUUCCACCUGUCAGAGAUACAGUUCAGUAGUACAUGCCUACACUGACACUUAUUU
UCCAAAACAACUUUCUUCCUACAAAACAGCACUUUACAAUGUGGACUAAGCUAAGAAUGGUGUGAUGUUGUGUGGAUCUUUGUGCAAUAUAGCUGUC
UGGUGAAUACUGUGUGAUUUGCAAAGCUCCAUUCAGGUGUGAAUCAUGUAGACCCUUCUUAAGUAUCUAAAGCUUUAACAAAAAGGCCUUAUUUAAA
AAUACUCUGAAAUGUAAAUGUGAUGUCAGCUGUUUUUUUCUAAAUAAAAGCUGAGAAUACUGUA

>*ZAR1L*, NM_001171543
CUUGUGUGCCCUCCUCCAAUUUGCGCUCUGUACUUUGUCAGUCUUUUGUGAUGUUUUGACCUUAGGCUUUUGACCUGGCAUUGGAUAAUGGAUGAAG
ACUUCUGUGUUAUUUAUAAAAUAUUUAACUUUAUUGUAUAUAUGCUGUAAUUAAAGUUCAAUAAAAGUUUGCACUGAUUUGUUA

>*MOV10L1*, XM_015273353
CUAAUUUUUCUGUGCUCAAGAUGCAUUCAGUCCUCUUCAGUUUUUGAAGCUAAGAAUACUUAACAGGAAUCCAGCACCUUUUGGCCUCAGGGAAAGG
CAUGCAUUCUGAAAUUCAUUGAAGUUUAUCCAAGAGAGGCUUCUUAAUGCUUCCGUUCAAGAAGUGAAAAAGUUGAAAUUUAUCAUUAUAGAAUAGU
ACUGGCAACCAGUUUGCUUCUUUUGUUUAGAAAGUGUAUGGUUGUUCUUCUGCAUAGAGCCGGUGGGUGUGAGUCCUACAAGCUUCAGAAGAAAGUU
AUUGCUGAACUUGUAUAUUAAUUUUUCUUUAACUUUUUUUCUUUAACUUUUUUUGACAUUUCAGUUUUUCUCAGACUUAUGUAGUGCCUCCUGAAAC
UCUUCCAUCCCAAGAAUAUGUUUUUGUGUUAUUAUUUGGUUAUGGAGUGUGACAUUUAUAAUCCACACUGGACUGAACUGGAAUGAUGCAUGAAUAU
CUUCACUUGGCUGAUGUUGAGUAUCUUAAGCCAACCAAACUUAAAGCCCUGCUUUUUUUGUUUAAAGCCCUUCUAUAUGUGUCUGAGAUGACACAGG
AUUCUGUUUCUCAGCUGCAGUCCAGAUGCCUGCUAUGGUAUACGUCAGAAUGCCUCAAAUGAUGGGUUUAAAAGGUGGGUGGGAGACAGUGAAAACU
ACUGUGUUUCUACGAAGAUUCUCUACAAUAGUGUUGUAUUUAACUUUGGCAAAAUGGUAAACAUGAACAUAAACUGUAGCUUUAUUGUGUACUGUGC
UGUUACCUUAAACUUGAAUGUGUUGUUCUCUCUGAGUUGACUAAGUACUGCCAUGCAGCAUUUCAAAAGUAAGUUGGACUGUUAGCUGUUGUCUUUC
AGAAACGGCCAGUACAACCCAAUGGCUUUCUGCAACAGACAGAUAUUGUAGGUAACCUACAGAGGAACAAAGAGGGGGUAAAAACAAGAUUUGGUUU
GCAGUCAGUUCGUAUUACUUCCACUUCUGGUGGGCACUGUACUUCCUUCUGCAAUACUUAAUGUGUAGAUAGGCAGCUUAUAGCUUGGCAAAAAAUC
AAAGCCAGAUGUACAGUACAGUGACACACACGUGCAGAACCUAGACUAUACUUGGUGUGUUUAUCCAGUCUGAGUCACAGCUGUAUUUGAGCAGCAC
UGCAGUCUCAUCAGCCUUGUCUGUUUGGUAUGUUAUUAUGCCAGCUUAAUUGCUUUUACUCCUGAACAAGUCAUAAUAGACAGUUUGUUGCAGUAUU
AUACUGAAAAUGUCAGUGGCUGUUAUGCCAUUCCAGUAUUCUACGUAAAACUUUGGAAUCUGUCCAAUAAAGAAUCUGUUCCCUGUUGAAGGAUUUC
UUCUAUGUCUUCCUUUGAGGAUUAGAUGGACCAGAUCUCACAGCAACAACGUAGUCCUGUUAAAUGAACUUCGAGUCCCCUUCCACUCCAAGGCAUU
CUAUGAUCCUAUGAUACUUCUGCUUAUAAGUACUUGUUUUUCUCAAGUGUUCUCAUGUUCUUUUUGAAGAACAGUGAAGUGGUAACAGAGAUGAAUA
GCACUUACUUCAUCACUAGAUUUAACUAAAAACUCGUUUGUGUUUCCAUGGUCUUAUGUAUAGUAAA

>*MLIP*, XM_015284903

CUGUAAUGCCUUAAAUUAGGAUCGAGAGAUAAGACCACAGAGUACUUUUGGGCCUUUUUCUCUGCUGAGAAGGAAUACACGUGUUAUCUAAAGCACC
ACUAAAAAAAAAUUAUAAUGCUUCCAGUAUGGUAAUUGCUAAUAUUCUUUUGAAAGAUAAUUCUAUGUACUAGCAUAAUUAACCCCUGAGAUACACA
UACAAACGUGCCAUAUUUAUAUACCACUUCUUGAAGUGCUUGCCAGUUUAAAAGAUGCCUAAAAUAACACAACAUAUUUUAUUUAGAAAUGAUAUAA
AUGUCAAAGUUCAAUAAAGGUGUAUUUUUGUUGCAAA

>*TERT*, NM_001031007
GGGAUGAAGGUCUAUUUCAUAAUCCUUCCAGGCUUAAAAAUAAAUCUUAAAUUUUUAGAUUAAUUUUCUCUGUAGAUAAUUUUGGUAGACUGAACUG
AAAGCAUCUCUACUUGAAAGUUUUCUUUUUUUUUUUAUGGGUACCACCCAAUGACCUUUAAAAGAGUAGACUGUUUUGUUAACAUCUUCAUGUUUGU
GUUUUUCUGUAAGUUCGAAGGCUGUUUGAAGUACAACCUGUGAACUUGACUCGAUUUCCUUCAGUAAUUCCUUUCUUCUUGGUGAAGACAUAUUUAA
CCUAUCUGUAUAAACAGAUCAAUAUUUUUGCCAGUAUUGUUUCUGAAGAACUGUUCUUUGAUUUUUCUAUUGCAGACAAUUUUACAAUAAAUUGUCU
ACAUAGUUGCAAUAAUGCAUACUUGCCUCCAAUACCUUUAUUUUGUAGUAAGCUUCAACCCAAUCCCACCCAACUUGGCAUUGUCUUGAAAUGCACU
UAUUGGUACAGGGAAAGUGCAUUUCAUGGCUGCGCCUAGCAAGAUAUGUGGGGUAAAGAUUCUUUCACUUUGGAGAACACAGUCAAAAUUUGUAUGC
CAAAUCUGGGUAUGUUCAACAUGUUUAAUAUAUUCUGAGAGUUUACAGAGAUGUACAGAACAAUGGUGAUGUAUUUAUAACUACAAAAACAAGGGGA
ACCAGAGUUAUUGAUUAGAUAAUAGAAAAGUAAUCUGCUUGGUA

>*CPEB1*, NM_001293219
AGCGGCUGUUGGGGUGCGGGCGCGGGGGAAAGUCCUUUUGUUAACCUGCCAAGUGGAGAGCGCACGAGCGUGCCUGCUGGUGCCAGACCCAGGCUGG
GAACGCGCUUCCUGAGGACUAAUGGGAAAAUCUUACAUUCACGUUUGCACUUGCUGGUCUUUUUGUUUCUGCACUAAUGCACAGUGAAUUUUGUCGG
GUUUUGUUUGGGGUUUUUGAGGGGGGGAGUGCCCUCUCAAGUGAUUCUGCAGUUCCCAGACUUUGGUUCCUAGUUUGACGAGAAGCAAAAAGGGCCG
CGUGUUGAGAAGGUGUUUAUGCAGAUGCCUUCACCUAGGUUUUUUAUUUAUUAAAGGCGCUUUUUUACAUUCCUUGCAAUACUGAUGGUAAUAAUGC
AGGUCUCAUUGGCUCCAUUUUUUUGCAGUUGCCAUACAGUGCCUUUCCAUUCAUUUAACCCCCAUCUGAACGGCAUAAACUGAAUGUUCAGCUGGCG
UUUUUUACUGUAACAACAAGGAGACUUUGCUCUUCAUUUAAACCAAAUCAUAUUUCAUAUUUUACGCUCAAGGGUUUUUACCAGUUCCUUUUUACAC
UCUUAAACAGUUUUUAAGUCGUUUGAAAUGAGAGAUUUUUCUUUCCUGGCAGCUUUUAACAUUAUUGCAAUUUGUGUCUGGGGGCUGCUGGUGGGAA
GUUGUAAAUCGAGGUGUUUGCAACAGAAACAGGACAGGAUUUCUGGAUUUGAAACUGGACCGGAUAAAUGAUCUCUGAGCUGCUGUAUAUAGUUUUA
AAUAGUUUGUUGCACUUAAGGGGGGUUGAUAGAGGUUUUUAAUCACAAAGUCACAGGACUUACUUUCCUUUUGUAACUAGCUAAAAAAGGGCUGCGU
UUCAGUGGACGGAUGGAGGUUCAUAGGAGCCCUUUCUCUUAGAGGGGACAAGUACUCUUCCUUUUGUUUCAGACAUGUAUGAAGUAAUGGUGCAGUC
GACUGAAAUGCUGCUGGGAUUUGAGAAUUUUUUUUUUUACUUUCCCCUCUAUCAGAAGCUGUGUGCCAAAGAACCAGUGUCAUAAUUUCUCCCUCUCC
UGCUGAGCUGAUGUUGCAUUGUUGCAUAUCCCAGCUGCUCUUUGUGGGGUUUUCGUGAAGCUGUGUGUGAAGUCUCUACCUCAUUGUAGUAUAUGCA
GGCAAGACUGCACUCUCCUACAGACGCGUGGAGUAAGCUGUGGUGUAGUUUUUGGCACAUAAUAAACACGUUGCAGCAAAAAGCCCUCUGUGCUCCC
UUCUUUCAGCCUGCACACACCUUGUGCAUGGGCUCUCUGCCCCUGGG

>*ACRC*, XM_025150071
CGGUGUGAUACGUUUACUGCUUUGAUGCCACUCAGUUUUUCUGUGUGACAGUAAAUAGAUGAGCCUAUUGUGGAGCCUGUGAGCGUGUGUUGUAGAA
GCUUCCAGACCAUUUGAAUACUUUGGAUUUAAAUACUUCCAAUAACGACCCUGAGGCUUUUUCUAAACUGGGCAAAAGUGAGCAAAGAGUAAUGAUG
AUGGAAAGUCCAUGGCAAUAAUGCACAGACCAAUCGGUCAGGCUCUCUGUCUUAGAAGACAGACCCUUCCUAGGACACAGCUGUUAAUGUUUGUGGU
UUUCUGAUCAGUAUUUACAUGCAGUGCUUCACGUGGUCCUUGACUCCUGUGAGCAAUGGGUUUAUUGUUUCACCUGUGCAGCUGACCUGCUCAGUUG
UGCAGAUGCAGUACAAUCUUGUGCGUGCUGAUCUGCCUGGGCCUGCCAGAAGAAAAGCCAGGUACCUGAGCAGCCUUCUGAUUCUGUCUGGGUGACU
UUGGGGCAGGAUAAGAGGCAGAUCUGAAGAAAACUUCAACAUAAUUCAGUUGUCCUCCCAAGGAGAGUUUAUCUGAGUGACCCUUGGCUGUUCUUGU
UGAAAGCGAUAGGUAAGCAGUGCCUUGACUCCAUUAGCAGCAGGUUUUGUGAAGUUGGCUGGUUCCUCUAGUGGGAUCAAAAUCCUCUGAUUAAAUG
UAGACUUCAGUUUUGUCUCUGUCACUGCUAUAUUUUUUUACCAUAGAAUGUGAAGAUCCAGUCUGAUGAUGCAACUUUUUCAUGCAGAGUGUGAUUA
AAUUAGUUAUGGCGUUUGUGUGUGCUGUGAGCAAAUCACUCAGGUGGCUGUUACUUUGGAAUAACCAAUAAAUGGCUUUUCAUACCACA

>*STK31*, XM_025147425
AUAAGGAGCUUGUCUGUCAGGUGUUUGACACUUGUUUUUGUUUCAUUUUGAGAUCAGUUGUCCUGAUUUAAAAUCUCAAAAUAUAAAAGGCUUGAUA
GGGGCAGUCAUGUUUUGUGAGGUUUAUGUAAUCGCUGCUUUCUUUUAAAGUACAUAGUCUAAGAGUUCAGAAAUAUGCGAUUAUUCCUUAAUUUGUC
ACCCUUUUCUUUGCCUGUUAUGAAGGCUUUGCAUUUAUGAAGUAUAGUUUGUUAAUGACAGAAGUAUUUAAGGUCUAGUGACUUUUGUAUCCAGUUU
UUUGGUGUUACCUUUUCAGUUAAGUUUUUUGUUACUGCAGUAAGGUAUUUCCUACUGCAACUGUCUUCAAGAUUUAAGAAAAAAAGCCUUCAGCUGG
UCAGGAAAAUAGGAAGCAAGCUGCCUCUUUUCUCUUUCUGCACCUCAAGAUUUCUAAAUAGCAGACACUGUUUCAGUUGCUCUUUCACAGUGUUGCU
GUGCUAAGUGGAAUACUAUGCACACGUGUGCUGCAGAAAUCACUGCUUAUCCGUUCUACUGUGUGUAUCAUCUGUUCUUAGGUAUUGUUGACUGCAG
UUUGGCAUUUUGAGUGUGUUUACUUUUCUGCAGUGCUUUUCUGUUAGCAUUACCCUUAAAAAAGUUUUGUUAGCUCUUUAUUUUCAUAGGAAACUGU
UGCUUAUCUGUAGUUCUUCUCUCUUGGGAGGGGGAAAGGUUAAUGUUCAGAGUUCCUGUAGCAAUAGAUUUAGCCUCUGGGUGUUGCUCUUGGACAG
UGCUGUUGGCAGACUAGUAUUUUGCAGAGGAAUUAUUGAAAAAGCCUGUUGUCAGAUGAAGAAUGUAAGAAUUUGGUGUGUAAUGGGCAAGAUGCAG
CAGCAGGGAAACCAGGAAACCCUUAGGUACAAUAAUAGUCAACGGUAGAAUAUAUCAUUCUGGCAGUUCCUUCUGUGAUAACAGGCUGGCUUGCCUA
UGUCACUGUUCUUUACUCAAAGUAGCAUGUAAAAUACCUGGUCCUGUUUUCAUUGAAUUGUAAUGAUUUUUUUUUUAAUUUCUAUUCUGUAUGUAAUU
AAUGUUAUUAAAACACUUGUUAAAAUAAUUUGGACUCUUUGGUUAGUGUGAUUAAUGUAUUUCUGUUGCUGAAAUCACAGAAGACUAAUUCCUUCCU
UUUUGUUGGUGAAAACAACAACUGUGAGCAGCUCUAUGGGAUGCUUUCCAAAUGCUGACUGAAGCCUUUUGCUGUUCAUAAAUGCUAACUUUAAUAU
UAAACACUUAACAGAUUUUGUAACUGUAACCUGAUGUUCUGUCAUUUAAUAAACUUUUGCCCUGUUUUCUGCGCCUGUUUUCUCCGCGCAGCCUAAA
CUGUACUUCUUAAAUCCCCCAAUUUCUAACUGCCUUUGUUUUCUGAAACCAGAAGUCUCUGCUUGUGCUUGAUUAAAAAAAACUGCAGCCUUUUCAG
AAUCUCUCCACCAGCUGUAAGUAAUCUGAUUUUUACAUGUGGAACCCAACAUAAAUGAUAAAAUUAUUUCUAAAAUGUGCAGGUAAGCCUUUGAAAA
CUUCUGGUAUAUACAAGCAUCAGGGUAUGGGACAGCUCUUCUAUUCAAAUUAAGUGUAGAUUUAAGUGGUGACAGUCCUACAGGGUCACUCGUUUUG
UUUAUUUCUGCUUUGACCUUUUCUGUAACGUAGCAUUGUCUGUCUUUUCCUUUGUUGUUUCCCAGAGGCCUUAAAGCAUGUUGGUUCAGGGUCAUGU
UGCAGAGUUAUCCUGCCAAAGCACGCUAAUCACUGUAAUGGACUGCCUGCUGUUUAGUUUGAGGCACAUUUUAUUAAACAGCAUCUCUUUUGUCUGG
AAGAUGGGACAAUGAGAUGAUACUUGCUAUAGAUUUGGUGUCUCUACUAAUUAACUAAGAAGCUCAAGCUAAACUUCCCUCAUUCACCCUUGUUACC
AUUUUGCAGUUUUAAGCAGUAUCCUUGCGUGGUCUAAGAGGCUAUGUACUGCUUCAUUUCAGCAUACAACAGCAUAUCCCCUUACUUCUAAGACAUU
CGUAUCUAAUGGCUUUGAAUAGUGAACCGGUUGUUCCAGUUUGCAGUACUGACUUAAAAAAAAAAAAAAAAAAAAAAAAAAAA

>*FAM110C*, XM_015284930
AGGCUUCCUGCGGCGUGUGCUGCAGCCUGGUAAAUGCAAGAAUCCUUGACUUGGAAGUUAAACAAGACCAGACACAGAAAGAAACUCUUCCUUCAUC
UGGCCCAUCUGGCUUCAUCUGACUUCAACUUUGUCCAAAUUCAGUAUUCUUGCCUCUUUACCUUGUCCUCAUCCCCUAGCAGCCUCACUCAUGUCAU
AAGCACAGAGAUAUCCAAGAGAGUGAUGACAACUGGAGAUCAUGAACUGUGCUGCAGCAAGGCAGCAGGAAAUGAAAAGUCCCGGGAACACAAAGUG
UGCUGUCAACGCGGAUAUCCACCAUCAGGUCACUACCCCAGAAUGUCACUCCAGGCUGUGGCAGAAGGUCCCGUUUCUUGCCAGUAAUGUUUGUUAU

GCACCCGUCUCUUGCCAGUAAUGUUUGUGAGUAGGAAUGGGAGGAGAAAGUAGGAAUGAAGUGGCAGUGACCCCUGGACAAGGUGUCACCCUGCAGG
CAAGCUGGCUCCACCCAGCUUUUCAAAGGCCCAGUCCCAUGAGGGCUGUUUGACACUGGCUCAUCCUGGCAUCAUUAAGUGUUUGCCUGGCUGGCAA
GGACUAGCUGUGGAGGCUUUAUCUGGGAGAUCUGGCAAACACAUCGUAAGCUAAAUCUCUGUCAAAUGUCUCCUUCUCUAAUGGUAUUCAGGCCACA
UUGUACUGCCUUGUGUCUGUCUAGUUUGCUGUGUCUUACACUGUGCUCAACCAGUGGGGAGGAAUAGCUUUUAAGACACUGUCUAAAAUCUUUUUAA
CAGCACAGUUCCAAGAAAAAGGUACUAAAAAAAUAGGGUUGCCUUGCUGCAAAGUGGUCUAAUUGAAAUUUAUGCAGGCUCCUCCACUGUGCUUGCC
ACAUCUCUGACUACUGGAGAGGGUUUCAUUACAAUGUAUUGCAGUUCAUGGCUCAACGCCUUGCAUUUUAUGCACCAUGAGUGAGCAGUUGGCUUGU
AGAAUUGUACUGGGAAACUGUAUACCUAUAUGCAAGAUCAAACUUCCCUAGUGUAGGUGUGGAGUGGUGUUGUGCUUGGAAUGGACAUGCUGGCAUC
CCUGCACCAUGAGCCAGAGGACCUGUUAGGGGCUGUGCCAGGUAGAUGUCCUAGUGGGAUAAGAUCCAGUGUACACACCACCUCACUCAGUAUUCUU
AGUAGAAUGCUUCAUUUAAACUGGCUAAUCAAAACACGCUUCAGAAGUACUUUGAAUAACCAGUGUAGUGAGUGGGGCCUCUACAAUAACUUAUAGG
CCUAGCUGGCCAUGCCCCAGGUACUUUAGUCUGUCAUUACUUACUAGAGAGUGUCUGAGGCCAAAGGAUUUCUUUGCAUGUUAAAACAUACACAAAA
AGAUGUGAAAUAAAAACCAAUCAAAGACAAUCCCCUUUUGGUCAUAUUAAGGCCAACCAACAACAGCUGUGCAUGCAGGAUUCCAGCUGGGCCUCCU
ACAGAGGUUAGGCAUGAGCUCUGUGUUGCCAUGGGCCUGGCUUCCUGCUCACAAUGCUGGCCUUCAGCUGGCCCUCAAACCUGGGUUGUGGGAAGUC
CAGAAAAAGGAGCUUUCAUUCUCCACAUUCCUACUUGGUCGCACAGAACUCUUUGGGCCUCUUCUGGCUCAGAACAGCCGCCAGAGCAGCCAGGUGG
AAGCAGAGCUCAAAUGAGCACUUGGGCUGCAUUUUUGAAGUGUUUGUUGCUCUUGAUAAAUAAACCUGCUGGAGUGCAAAGGGCAGAGAAUAGUGGC
ACUCCUAGAAAACACCUAUCGUGGGAGGCGCAGUUUCCCGUUUUGAACUUACACAAAUGUGGCAUGCUGACAGAGUUGCCCUUGAAGCAUUCAUCGA
GUGCUGCAGUUGGCAGUGGUUAAAUGCACAGCAAGAUGGCUGGGCUGCAUUUCCAUGUGUCUAAAAUAUGCUUGUCAGUCACUGGAAGGGUAGUUUG
CAAUUACAGCAACUGUUUGAGAAUGCAGGGCAUUUGCCUUCAUUUUCUAGUGUCACAUACUAAGGUCUGCGUGUCUGAAAUUGUCUAGGUAGGUAGA
UAACACUUCCCGAUAAAAUAAUUGUUGAACCUGUUUCAAAGUUAAGAGAGAACCAAAUAGUUUGCAUGAGUGAACCUCUGCCAACUUAGAGGGAAGC
UUGUAUGUGGAGAUAUCUUUGUUAAAUAAUGAAAAUUCCAUGUGCAUUUGUUACAUUAUUUCCAUAGGAUGAUUCUUAGAAAACUACAACUCAAGAU
GCUGCUAAUAUGAUUUAUGCAAUUAAGGUGCAGUUUUUAAAGGUACAGAGGCUUGGUUUAUUAGCCAGGAACACAACCUACUGUAUCUGUUGUUUUC
AUGCU..... (truncated)

>*BMP4*, NM_205237

CCCCUUCCCUCCCCUCCCCUCCUCUCUCCCUCCCGUCCCACCCCAGAACUGCUUGCUCUAGCUGGACUCUUCCUCUAAACUAAACGUUCACCUUGAC
CUUAUUUAUGACUUUAUGUGCAAAUGUUUUUUUUUUUUGACAAUAAUGAUCAUAUAUUUGACAAAAUAUAUUUAUAACUACGUAUUAAAAGAGAAA
AAAAUAA

>*SPAG6*, XM_025147182

ACUUCAAUUUUUAUUAGAGCUGUAUUUCACGUUUACGCUUUUAUGACUGUAAUACAAAUACAGCUGCUGAAACUCUUGUUAGAUUCUGAAAUCUACU
UCUACUGUUUGAACCACAGCAAGACCUCGGUAUUUCCAGCAAGCUGUGUUUUGUUCUUUAUAAAAAUGUUGCUAAGUUUUUGUUCCUAUUUGUUUUU
CUCUAACUUUAUGACAAGACACUGCAGCAUAUAUCAGUGGAAUUUCAUGCAAGGGUGUUGAUUUAUCACAGAAGCUUUUUUAGAAUCAGUCAUUUCC
UUUUCCUUCCACAAGAAAGGUUUGUUAUUAUCUCAUUCAAAUUUCAUCCUUCUCAGCACUUUAUCAAUUUUCCCCUUUUGUCUUUGCGUAUUGUU
UAGAGCUUCCCAAGCUUGAUGAGGGGGAGGAAAAAGAUGUCCCCUCUGCCUUUUCUAAUUAUAGAAUUCAUUCUAUGCUGAGAUUUUUUUUUUCCCA
CCACUACCUUUUGGAAAGUGAUGUGAUUUUUGCAUCCCCAAACAUAUUAGUAGCUGCUAAAUAGAGAAGUAAUCAGAUCUGAUGAUGAUAAAAGGGC
UGUUUUCUGCAUGCUACAAUAGUGUCAUGAUCCUGGCAUGCUAAAUAGUACAUCUGCAAAAAUCCUCCUAAUUAAGGCAAGAUUUGCCACAGAGGGA
AGAAGCGAUACAUUAUUUGGUGUGUAUGUUUUUGUUCCUAUCGGAAAGGAUAGAGUGGACUCAGAAUUCCCACAGGUACAAAGGGAGAGAAAGGACU
CUGUGAGGUUCUGCCCCAUUUGGGUCGGCAGUAUCUGGGCCUCUCAGGAAGAGGGGAGAAUUGUUUGGCUUCAGGAAUGCUGUUAGCGAAGUUUUGA
GCACAUCCAGAGGAAAGCUGAGGAUACUUGAGUCCCACUGAAUUGAGCGGUGGGACUAACACUGCUCAGUACUUUCUCUUCAAGAGAUGAAGUUGCU
GCAGAAUUGAGUUGAAACACAUUUGGAACUUAGUCUUGAACUUGCAAAUCGCGUUGACAUGGAGAUUAUUGGAGGUAUGAGCUCUUUUGUAUACAAC
GUCCUGAAAAUACUUUUGCCCUCUUAUGUGGUGUAAAAAGACUUUCUACAUUCUACUUACGCUGCUGGUGUACUGUAGCAACAGGGAACAGCAUACA
ACCCUACAGUAUUCACCAAGUAAUAGGUAAUUGUCAUUCACCAUUUUUUGUUCUUCUAGCCAUUCUUCUCUUCCAAGUAUGCAGCAGGAUUUUUGUU
UGUUUGUUUCUUCUCUUCUUUUUCUUUCCCAGAGAGUCAUAUGCAGUGUUUCUGGGAUGGGGCGUCAAAAUUCUGGCAGCUGCGAGGGAAUACAAUA
CUUGAAAACCCCUGCAGGAGAAACUUGUCUACUUUCUUAAACAUAUUAGCUGAAUGAGUUUAUAAAACAGGGAUAGCAUCUUCUGUUACUCGUAAAA
CAGGAGCAAAGUCUUAAUGCAGUUGGAAAUGAUUUAGAGUGAGUUUUCCUUGCCAAAUUCCAAAUUACUGUAUUCUUGUCAGCAUAUUUGUCAUUGG
UUCACAGAAACAUUGGUGGGCUAUCUUCUGCUAAGGUCUACUUCUACAGAGGGCUGUGCUCCUGGUGUGAGGUACUUGGUGCUCUCGGACUAGUUUG
AGGAGAAGCAGAGGCUGUAUAGUUGUCUGUAUGGAGCAUAACUGAGAUGGAAACUGUUGGUGUCACGACUAUAAACCACACCAGCUGGGUUCUUGCC
UUAGCCUGCACCAGAAUGAACAGGUUUGGAACUGACUAACCAAAUGUCUUUUCCUUUUUGGUAUUUACAGUUUGACAUUAAUAAUGUUACAACACUG
GUGGAAAAAAAAAAUCAUUAGGUAUAUUAAGAAGAUUGUUGUAAUACUCAGUGAAUUUUCCUAAAUGCAUUAUGGAUAUUUAAAAAUCAAAUCUUAA
ACAUUCUUUGAAAGAAAAUGUCAUGCAAGGACUACCGGAGAGUGUUGAAGGAUCUGAAGGCUUUGUUUUCCCUAGCAGGGGAGAAAUACUGUAGAAG
GCUGAAUGUAGUGCAGCUGGGAGGCCGGGUAGACAUUCAGCUGUGUACCCAUAGCUGCAUCAGAUGUUUUGGAGCACGUGCACAGCACACGAUGCUA
ACGGGUGUGUUUAGUGUAGAGAGAGCAUAGAGGCUUCAGUUCCCUGACAAAUGUGUUACACCAUAAUGAUUCCACUGGGUUAAAUUUCAAUGCCUAA
ACCCUUUGUCCACAGUCUUCUCACAUGUUUUUCUGAUUCAUGACAAUGGAAGUGAUUGCAGAAGGCAAACCUUCAAAGCAAUCGGAUUUUGCAGUGC
ACAGUCUCAAUCAGACCUAGCAUAAAUCCCUCUUUAUGCCACUUACGCAGAUCU..... (truncated)

>*SRSF1*, NM_001113741

ACGAUCACUAGCUCUGAUCUUUUUGUAGAACCCCAUGUUGUACACAGUUUUCCUUUACUCAGUACAGUCUUUCAUUUUUUUUUAAUUCAAACUGUUU
UAUUCAAAUUGGCUGAAGUGUUGAAUUGCAUUCUUGUGUAAAAUCCCUAGUGAGUAUUUGCUCCGUAACAACGACAUUCCCCUCAUGUCUUUGGUAA
ACUGUAUUUUAAUUGAUGUCAGUCAGGAUUGUUUCAAUUUAGUUAUAGUUAAAUACCAACAUUCUUCAAACUGUGGUAUUGCUGUGUAAAUGUCUCA
GUGUUCAGCUAUGGUUUAUGCGAGCUGGGGAUGUUGGGAUGUUUGUGGCUAACUUGUCUCUUCUGGGGGAUCUUAUAUUUUAUCUUGCCUUUUCGUG
UGUCUUUCUGUAGACAUAUCUGAAGAGAUGGAUUAAGAAUGCUUUGGAUUAAAGGAUUGUGGAGCACAUGUCAAUCAUUUUAGGAUUGUCAAAAGGA
GGAUUGAGGAGGAUCAGAUCAAUAAUGGAGGCAAUGGUAUGACUCCAAGUGCUAUUGUCACAGAUGAACUUGGCAGUAUUGACCUUAUACUGAGACA
GGUGAAUUGAGAACGUGCGCCUGCGUGUCGCUUCUUCAGGCACCUCUUGGUCACUGCAGGCUACAAUUGCUCUUCUCUUUAAAACCCUCUUGGCAAU
UGUGGCUCGGUACUUGCUGUUGUAACUCCUUAUUCCAUUCUAGUUUUUUUUAAAGGAGUAAAGCGGGACAGGGGUGGGAGUGUUGGUUUCCAUUGUC
AUGCAGCAUUUGGUUACAAGUGUUAAUGUUCCGUAGUCCUACACUUGCAGAUCAAGGGUAUCUUGAAAUGAGAGUAUAAUAUCAAUACUGCUAAAAU
CUGCAUGUCCUCUGUGUGACUGAUACAGCGUUGCUAUUUCAUUUUUUUAAAGUAUCAGAAUGAAAGACAAAAAAUAAGAGAACCUAGUUGGGAAUAG
UUUGUCAGCCCUUCCCCCAGACUAAUUCAGAAUUGACUCACCGGUAUGCUUGAGAACUGCAGUUAGUUAACUUUGUUACCUUCCAGCUAGGGGUGCU
AGCUGUAGCUUAGAGGUGCAGUAGGCGGAUAGCUUUAGCAGUAAACUUUUCCAAUUUCAUAAACAUUUUUUGCAACCACAAAACCUGUAGCACACCA
CCGUACGAGCGAUGAGUAUGUUGGCAUUUUCAGUUGACAUAUGUAAAUAUUACAGUGUUUCCUCCAAAUAAUUAACAUUUCUUAAAAAAAAAAAAAU
AUAGCAUCACCAUUCAGAAGCUUGUUUUGGGGAAUUGAUAGUUAUUAAUGUACAUCUGUAUUAAUUGAUGGCAAACAUAACUGAUCAUUCCCCAGAA
UCCUAUUUUUUCAUUGCAGUAUCUAACUUUUUGCCUCCUCUUUUUUGGUUUUGCUGGUUAUAAAGGUUUGGAUUGGAGAGGGCUCACUGGAUCCCAA
UCCUUGGAGCUGGAUCAUUGGAUUCAAAUCAUAAUGUGGAUAGGAUAGGGAGGCUGAAUGGCAAGGAUUCAUGGAGCGGGAUCAGAUUACCAGGAAC

GUAGGAGUGGAUUCCUGCCCCAACCAAACCGCAUUCGUGUGGAUUUUUUUAUUCAACUCAAUUGGCUAUUCCAAAGAUUUUCUUUCCUAUUUUUGA
CGAUUGGAGCCCUUAUGAUGCACGAUGGAGUUUUCAUUUUUUGGUAAAAGGAGCAAAGCGAGGACCUGGAGAGGUACGCUGGAGCAAUCUCCUUGG
AAGGAUUCAGCACGAGUAGAUGGUAAAUGUGAAAUGGGAAAAAGGGGGGUUUGUUUAAACAAAAAAAAAAAAAAGUCAUUUCUCUAAAUUUAAAGUCA
AAAAUUGGAGUUAAAGAUUAAGUAGGCUUUCAAUUGGCUAUUGCGUUUUUUUUCUUUGACAAAUAAAAUUUUUAAAAAAUC

>*TFAP2C*, XM_417497
AGGGGAACAAACAGACUCGAGACUCUUCCUCCUUAACGCAGACUGGGAUCUUCUUGCCGGGGGGAACGUAGCAAUUUGGGUACUUUUUUUUUUUUCU
UCUUUAUUAAGAAAGGAGGGAAAAGGAAACGAGAACAAAACCAUUUGGGAUCUUCACUCCUCCUGGAUCCAGGAGACAAGUCUUUCCAAAAGCUGCC
GCAUCCCUUCUCUGUGGAAGCAGUGACCUGAAGAUAAGUACUUGGUACCCGUGUGUUGCCAUCCUUUGCUGUAAAAGUGGUGCUAUAGGGGUUUCUC
AUGGAAGUGUACGAAGAGAUGAAAUACGUAGAUUGCUUUCCCACAUCUCCGUACCUGGUGGCGACCAGCACCGUGUGUAGUUCUUAAGAGACAGAUU
UGACAUGGGAGGUUAUUUGUGAUCAGAUAAAUUUUCAGGGUUUUAUUCUCUCUUAAUACAACAGCAACAGUAAAGCAGUAUUAAACAACGCUAUAUA
GAUUUAUGUACAUUAAAAUUUUUUUAUAAACACCCGAAAUAGUAAGAUUACUCUUUUUCCUUUUUGUUCUUUUUCCCUUUAUUUUCAGAUUACCGUA
AGAGAGUGCUAAAAUCCUGGUGGAUAAUGUUAUCUGAUGGCAUUGGUCAUACUUAACUCUUAUGAAAACGUUCAGGCUUUUAAGCACUGCAGUCGUG
UUGGCUGCAGUCGAAAUUAUCUGGGUAUUUUCUUAAUGAGGAGCAGUACAAAAUGAGAACGCUUUUAGGAAGUUCACUUUCUGUGUCUGAGGAUAUU
GCAGCUUUAUUCACCUAUUUAUAUUGUCUUUUUUUUUAAUUGGGGGGAACUUGGAAAAAUAGGCACAUAACUUUUUUUUUUUUGAACUUUUUUUUUU
UGCCUCGUAAGGCCAAAAAAUGAUUUCCAGGGAGGCUUUAUGUGUAUCAAGGGAAGCAGUGUGCUGAAUGUAAAUAGUAAUUUAUGUGUAAUUUAAU
CGGAUCUGUAAAUAAUGGUGAACUUUUUAAUACUUUUUUUUUUAUAAAUGGGUUUUCAGAUUUUAAUGUUGGUAAGUAUUUCAGAGGUAAUGGGUAA
GCUGAACAUAUCUUUAGGUUUAUGCACAGGUAUGUUAUACAGGGUAUUUAAGACUUGUUUUUUUUAAAUAGCGCGAUUCUUGCCACUGAAAAUGAGA
UCUAAUCUGAGGUGUGUGUUCACAAAGCAGACACUUUGUGCAUCAUCCACCCGACAGCUGACCGGACAUGCAAAAUGAGUGUGUGAGUUCGUGUGUA
UGUGAAUAAUGCAGGUUCUGAACUAUCUUUACAUAUUUGUAAAUGUUCCCGCUAUAAUUCUGAUGUAUAUGAUAACCAUAUAAUAAAAAAUAUUCUG
GAUAGAA

>*CYP26B1*, XM_015286068
AGCCCACCAGGGAUGGGAGGCUGGGGAUGGAGGGGUGGGGUGGGGACAGGGGGAUGAACAGGAGGGGAGAGGAACGAGGAGCUUCUCCACGACGCUC
ACUUGGCAGAGCCUCCAGGUUGGGACUGUUUCCUCCACCGGCAAAGCUGCCAAAAUCCUUCUGCUCCGGGCAUGGAGGAGCUUGGGGUGGCAUCGUG
CCUCUCGCUUCCUCCAGAAAGAGGAAGAGUUGUGAAUCCCGAGUAGAACUGGGACUGUCUGACCAUACACAGUAUCUAAAUAUUAUAAAUAUAUAUA
UAUAAAUAUCUAUAAAACACUUCUGCUGCCAGGAGGAGCGCUGCGAAGCCCCGACGGUGGGAAAGGCUUUGGGAACCAGCUGGCAGCCAGUGGUCUG
GUGGCCCGGCGUGGCCCGCCAAGCUUGUUUUUAACAGUGUUAAAUUAGCUGGUGAUAACAGUGUUUUAUUUUUGUUGUUGUUGUUGUUCGUUUGUUU
GGUUGUGUUUUUUUUUUUAUUAUUUCGGGGCUCUGGGGGCUGCCCUGGGUGAAGGGGAGAUGAGUGAUGCCCAUUCACAUCCCAUGCCCUUGGGCAG
GUUUGGGGGAGUGUUGCCAUUUCACCUCUGCCGCGGGACCAAAAAACCCUGUGGUGCCUUGUCUGGGGCUGAGCCUUCCCUCCAAAGCCUUGGAUGA
GCGUGGUUUGGGGUAGGGGGGGAGCUCCAUUUUUUUUUUUCUCCUUGAGUUUCCUGCUGCUGCUACACAUCCUGUGUGCACCGCUCCCCCCUCCCCA
CGCAGAGGCAUCCGUUUGGGAGCAUUUCGGUGCCUUUGCUCUCCCUCAGGGUGCGGGCACCUCCGCGAUCUGCCGCAGCUCUGUGAGCACCGAGCUG
CUCCGCUUGGGUGCACCCUCUUCCACGGGAGGCAUUUUUUGCAUUAAAGGUGAUGUCUAUAAAUAA

>*GDF3*, NM_205211
CGGAGUGGAUGUGGGUAUGUGCUGUGGGGUCCCGUGUGGCACACGGAAUGAUGGAUGGAAUAAAUCCAAGUGUGACA

>*SNRPB*, NM_204599
GAUCCUCUACAACAUACCUUAGAACUGUGAGAAGACUAAUAAGGAAACAUACUGAGCAGUAUCACUUGUUUGAACUUUGCUAUGUAUAUUUUCUAUU
UACUGCUUGUAAAGUUUGCCCUUUUUAAUAAAGUUGGAAAACGACAGAGUUU

>*BMP8B*, XM_003642583
GCACCCGGCAGGGAGAUGGGGCACCGCGUCCUCACUGUGCGUGGGGGGGGUUUGCUGCAGCAGCACCAGGGCUGCCCACCAGAUACAACACCACCCC
CUCCGCCCAACCCUUCCCUUCCCAGACGUGGAUGUAAAUAAUAUAAAACUUCCCUUCUCUCACCACACAUAAUGAGAAACAUCCAUACUAUACAUGC
GCAGAUAUAAAUAUAUAGGAGAGGAAGAGGAUUUGUGGUCAGUGACGUGGGGCCGUGGCGGAUGUGCGGUGCCCGGGGUGAGGCAGGGCGGCUCCGG
GGACAGCUGGGCAGCCUGGCUCCAAGAAGGGCUUUCAGUUCCCUACACAAAAACACUCUCAGCCAAUUAAGUAGCCGGCUGACUAACACGCUCCUGU
UUACUUAGAGUUCACAAAACUCAGCCAAGGGGAAAAGAAAUAAUACAUAUUGAGCUACAACGCUGUUAUAAAAUAAAUGCCUAGUUUGGAAAGUAAA

>*TET1*, XM_025151680
GCUUGAGUAUCAAAUGCCUCCUCUUGGUGCAGCGUUACGCAGUGUUUCUUUAAGGUGCUGUUAAAGAACAAGUCUCGUGUCGUUUACUAUUUGCUCA
UCUCAACCAUUUUAAGGCUGAGGUAAAAAAAAAAAAAAAAAAAAAGAAAAAUAGGGGGGAUUUCUUCUUAAACUGUGACUUUAACAUUUGGUGGUGCUC
AAGCACAUUUUAAGCAAAAAAAAAAAAAAAAAGUUGUAUAGUUUUAAUACAUUCUAAAAAAAAAAGAUUUUGUUUAGGUUAUUAACCUUGAUUGAAAUCA
UUCAGUUUAUUACCUUUAGGAAUUUAUAUUUUGGUGCUUUAAAUCAAGUCUGUUUACUACAAAAAUCAUUUAUAGGGAUUUUAACAGGUUCACAUUU
UAUGGUGUUAAAUCAAGUUAUACAGUAAUAGCUCUACACAGCUCUUGGUGCUUAACCACAUCAAACAGUUAAAAUAAAUAAGCUGAAUUAUUACUUC
AUGGUGCCAUUGCUUUAACAACUUCCAACCAUUGCUACGUAAUACAAAUUGCGGAGAAACCUUUUAGAAAACCAAUGAUUCUUUUUCUCUACAUUUG
UCCUUCCGUAUGAAAUGAAGUUAUUGGCAAUGAUGGCUGGAAGUUGUUUUUAAGCUGUAAAUAUAUAUUUUAAAAGCACUUUCUAUUUUUAAAAUUA
ACUUGAAAUAGUAUAGUAUAAGGAUCAUAUUGUCUAAGGUUUGUGCAUACUCUACAGAAGAAAUCAUUUUAUUCUUGCUGUGUAGAGUUCCAUAUUG
UUACAGCUGCAGUAUGUAUGCUAAUAGUAUGUGAUUGUUUUUAGACUUGCCCUUUUUUUGUGGAAGAUUUUAUUACAUAUAAACAACUUAUAAAGUA
CAGAAACAUUUUAUUAUUUGAAAGAGAGUGUGCAAACAUGAAAUUAGGAGACAUCAAUACGUGUGUUGCUAUUGAGGAGUUACGAUUUUUGUUUUUU
UAAAGGCAGCAUUUGUGACGAUUUCUUUGAGCAAUAAGUGCAUAAGUCUCAUUCAUUUGGAACAUUAUAAGUUAACGUGACAGCCAAAUGUUCCUAA
GACCUCAGGGGUAGAAUUGUUUUGGGAGAAAAAGUACCCCCUAGAGUUGCCUUAUUCUAUUCUGAAAAAAAAUAUACGUAUUAAUUUGAGGAAAAUA
UAAAUGAAGUGUUGAAGAAGAAUAACAUUUCUAAUAUUUGACAGCAAAUUUUUUCAUCGAGAAAACCCAUCUGUAAUCUUCACCAGCUAAGUUCAGG
UUUUUUACAAAAAUGUUUUGUAAAUUUUUAAGUGAUUUAUACAUUUUGAGUAGAAAAAGUUGUAUUCUUCCUUCCAAGCAUAUGAAUUUUGUUUUCU
GGUUCUCUUGGAAAAGUGUCAUACCAUGAAAUGAGUAUUUGCCAAACCAGAGAGAAAACAGGCACUCCAUUGCUUUUAAAUGCUGUAGCAUCCCACU
CUUCUUUCUGAUUCUCCUCUCCUAUGUAUUUCCUUUUAAAACUUAUUUAUUUAAACAAAUUACCAAAUGACAAUGUUAGCUAAGGACUGACCUCAUC
CUGCAACUUCCUCCAUGAGAACAGUUCCACGUAGCCGUACCCGCCAGUCCGAGUGGGGAGCGCUGCUCAAGCCAGGCCUCUUCUCCUGGCUGUGAGC
UGCAGGAUCGGGGCUCAGAGACAAAGCUGUACCUUAAGUCUGCCUCAGAGUCCUCCCGGCUGGGUAACCCAUGGGCGUAACGAGGUUAGAGUUCUCC
AGUGUGAAAGUAUACUAAUAAUGUACUUGAUAUUAUUAUACUGUAGUGGUGCUGUAACAGAGGAACAUGAAUUUUCUAGGUCUCCUUUAUUUAAAAU
CUAGACAAGGUGCUUUUUCCUUGAAAAGUUGAACUGUAAAUGCAAUGUUUACAGUUUUGGUACGAAAAGUACUUAAAUGGUAUUUUUAACAUAUAGU
GCCAGUUAGGACCUUGGUGCUACAAAUACUUAAAUACGGCCGUAGAUCAUUGAGACUGUUCUCCUGUAUAAGCGUAUACACACGUAGUGUUGGCAGG
AGCAAGGUCUGAAGUAGCAAUCACUAUAUAACGACCUGGGGUUCAGCUGCACGGAUUCCUGUCGAAGUUAACAGAAAUGUUGGUGGCAGAAGAUGGC

UGAAUUGAACCCAUUCUUUGUAUCUUCAGUAAAAUUUGUAAAAACCGGGUAUGCUGCAAAUCUUAGUUCAGAGCUUAAUUUUAGUUCAAAUAGAGGA
UAUGAAGGGAUUGUAAAUAAUAAUAAAACAAAAACAAUAUUACACCAU..... (truncated)

>*RBPMS2*, NM_204869
UUUGUUACGUCUCUCCUGGGCUCCUGUUCCUCCUCCCUGCCUCUCCAUGGAGGGACGUGCCAGGGAUGAUGGCUCCCACCUCUCACUUUGGUUUGCG
GCCAGCGGACAGAGCGCCCGGCCCCGGCGCAUCCCCCUCCGGACUGUGAAGCCGCCGCAUGCCGCCUCCAUCCCUCCGUCCCUCCAUCUAUCCGUCC
AUGCUGAGCUCCACCAUCCGGCCGCGCCGACACCCGGCAGGGGCUCGGUCCCAGGCUCGGCUGAAGGAAACGAAAGGCAAAUCGGGUUUUGCAUGUU
UUCUGGCAUGAAUUAAAACACUUAACUUGUGUAUGUGUGAGUGUAAGUUUGUUUGUUCUAGCGUUUGUGUAACCGUUAGCAACAGGUCCUACGGACU
GAUCCAUCGAGGUUUUCAUCUUGCGUCGGUGAAGACUUGGUUUUGAGAAAUACGAACUGUCCUUGCCUCACCCCUUCCCCACCUCCGCCCUCCCCCU
UCCCAGUACGACCAGAAGAAUUUGAAAAAUGUUUUUCUUUUUUUCCCCCUUUUCCCCCUUCCCCCCCCCCUUUCCUCCAAGUACGAUGCACUGGGUU
UCUGUUCUCAUCCAUCCUCCGGUUUUAAAAUCAGGGUUAUUAUAUUAUUUUUUUUUUCAGACUCCUAUAUAUAGAAAGGCUGUAAGCUAUCAAACCU
CUUCUUUUUUUUUUUUUCUUUCCCUCUUUUUUCCACAUCCUUCUGACUGGAGAGCAUCCAGUCCUGCCAAAUAAUCAGCCGUCCUUAUGGGAAUACC
GAACAAAAAAAAAAAAGUACGAGUAUUUUUGUACCAUGUGUAAUAAGGAAAUAUUUAUGCAUCAUAAAGAAUUUCUUGUGUGUUUGUGUGCAAUUAAU
UAUUAUUAUUAUUAUUAAAAGAGAAAUUGUAAUCCUGUAAGAUAAGUUAAUGUUUAGUUAAAAGCUUGAAAAGAGAAGGGUUGUCUUCUGUACUAAU
GAUUCAGUCAGGCAUCGUAAAUGAGAAAUUGUGCAAUUUUUGUUUUAGCAUCUUAUUGCUUGGUAUUGAAGUGCUUUAAGUAUUAUAUGACCAUGGC
UGUCUCGCUUGUAUUAAAAGAUUUGAGAAUAAGUUGCUCUAUACUUGCUGAUCCUAUGAGAAUGUGAAACUGGAUAAUAUAUGAAAUGCAACAAAAG
AAAAAAAAUAUAUACCCAGAGGCUGCUGUGGAAAAAAAAAAAAAAAAAAAAAA

>*MAEL*, XM_004938325
AUUCCUGAAGAUGAAAUAACUGCACUUUUGUUACUAGACCAAAACCUAUGGUCAGAAAGUCUAUCCAAAAUCAGAUUUUCUCACAGAUGUUGCUAUC
UGAUUUGUAACACCUUAUUUUUUUGUCAUGAAGUUCUUUCUUGCACGCUUAGAUGUCUUGAGGCAGGGCCGUCUUUAAAAUACUGUCUCUCUUCUGG
AGUUGUAUGUGUGGAUUCAAGUGGUACCUGUUGCAAGUAUUCUGUUACUCCUGGAUCUAUAAAAAGUGCUUGAUUCAAGUCCUCUUCUCAGAGCUAA
AAUCUUUCUAAUACAAAGUAUGUGCACUCAAUUCAUUUGUGCAAUACGUGACUAACCUUCAAAGAGGAGCAGAAUUAAGUUUGCAAGACAAAUAACU
UCAGAAUUCUGAGUUUGCAGCUUAUGUUUUUGCAUAGAUAAUACAUACUGAAUUAAAUGCUCAAACUGUUCCAAAUAUAAAUCAUGAGAUCUUGUAU
GGACUUGGUGUUUGCGGAGGACGGAACCCCUGUCUCUCAGUUUUCCUGUUAGAAAUAUUGAAUGCAUUCCACUCCGCAGCAUACAAAUUUCAUCUGU
AAACUGUAUUUGAUUUAAUAGGUGAUUCUCCAUGUGAUGUGAAGAGUUUUCCUUUUGGAACAAGUACCAUUCGAGGAAGAGGAAUUACUCGAUGGCU
GGAAAGUACACCUGCUCUGAGCAAAUCUUUCAGUAACUGAAAAUCUUCAA

>*SNRNP35*, NM_001277175
CUGGCAGCACCUUGUCUUAGGGGAAUGAUGGGAAGCUUGGGUAUGGUGACUAUUCUUUUCUGUCUCUAGAGUAGUGUGAGACACACAGUACUGCCCU
UACUGCUGGCUGGGGCAUACCUCUGCUGUCAUGGUGAAUGAACUGGUAUGAAGGAGGUGAUGCAGCAGGUUCUGGAAAAAAGCUGUGCCUUCUGGAA
GAUGUGGAUGUGACUCAGUUGUAGAUGUCACCUUCAGCAUGGGGCUGUGAGCUGUGUUACCCAAACAAGCAGCAUUCAUGUUACUGUCUUGUGCCUU
UGACUUUUAUCUGCUAGAAGCCCUGUGUAGUGUGGUUCCUAGUGCAGAUAUCUUUUGUUGAAUUGUUCUUCAGAGAAAUUAAACUUUUCUAUUAAUA
UUUUAAAGUGCUGAGUAAGAAAUACAUGUGCUACCAUCCCAUCG

>*TDRD9*, XM_025151084
GUAUUGCUGGAAGUCUUUCAGAGGAAGAAAUAUUACAGCCAGUUCAAGAAUGUUCUGUCUUCUACAUAUAACCGUUAGAUAUGUGUUUCACAAGACU
GUUCUUUAACAUUAGUGUAAGUGUAUUAAAAGAUAGUUUGGUAUAUUUUUAUAUUAAAAGCCAGAGGUAUACUGACCUCAAGCAGAUACAUUGAUUU
CAUAGAGCAGGAUGCAGUUUUCUGUUGAAUAAUAAUUCUGAGCUGGUUUAUCACAAGGCUUGUAGAAGGCAAAAUAAAUUAGCUUUAAUUGUUCAUU
UUCUGUCUGAAAGCUCAUGAGCCGUGGCUUAUGAUUCAGUCUUGCAACUGACAGUUGCAUUUUCUUUUGUCAUUACUUUUUAUCUUUUUUAGACCUA
AAAGCUUGGGGAAGCUGAAUAUUUAGGUUGUAUUCCAAUUGAAAGUAUAACUUUAUAUGUCUGGUGCAAUUUUUCACUAAUUUUCUCACAACAAAA
CAUAAUGCGUGGUUCCUUUUCCUGUGUGGCAGAAGAAUGCUUUUGAAUUACUGAUUCAUUGCACCUUUGUUUCAUCCUGUCUGUUUGUUUUUCUGAU
UCGUGAGAUCUUUAGCUCUAAAAAUGAGGUCAGUGGUUUCAGAAUAAAAUGUAGAAGUGAAAUAAAAUGGAUUUAUCUGUUCUGUUGUGCAGUCGUG
CUUAAUAAAAGCUAAAUUUAGUUACUGA

>*CDYL*, XM_418964
UGAGGUGCCCAUUGACACUGGAAUUGAACUUCUUGGACAUCAGGGCAAACCAAAUAUGCCCGUUUUAAAACUUAUAUAAACUCACCCCUGGCUCAGC
UUGGGAACAGUAGAAAAACCCCUCCUCCCCACCCCCCCAUGUUAUUGUCAAGGGGUAUGAAAGUACUGUAUCACUUAAAUUUGAACAAAACUCCUUU
CUCCCUGAUUGUUUUGUGUGUGUGCGCAUAUAUAUGUAAAUUAUAUAUAUAUGCACAUGCAUAAAUUAUGUGUGUGCACAUACAUAUAAACUUUUAU
AUAUACAAAAUGUAAUGUGGGAGUGUGUACAUAGACACAUGCACACACCAGGUAAACAAUGAUAGAGUGCACUACCAACCAUCUAGUUUUGGUCAUC
AUUGGCUAGUACUGUAUCAAACAGAGAACGGAUUAAAAAGUGAAUGGUGUUGCAUUAUUUUCUGUAUGGCAGGGAAGUCUGGAAUAAUGCUUUUCUU
UUAUCAUUAGAAUACAGAAUUGAAACGGGUAUUAGCCAGCCCUUACUUUCACUGCCCAACAAGUCAGCUAAGAUGUAAGGCACUGUCUCAGAAGAAG
GUUGACAUACCUCAAUGGAACAUCCUAACAUUAUUCAGAUGAGCUCCUUAAAGUUUAAAUCAAAAAUAUCACCCAAAAGAGAGUGUGCUCAGGAUUA
AUGCUAAGGAAACACUCCGCCCUCUUCAGGGGAGUGGUAGAAAGGGGUGGGAGGAAGAGAGCAGGAAUGUGUUUAAUUCUUACCCAGAUAACGUGAA
GAAACCACUGUCUCUUAGGGAAGGGCAAUGAGGGGAAGCAGGGGGAAUGUGGAGGGGAAAGGAAAAAAAGACAAAAAAAGCUUGUUUUGCAGUAUUA
GUGAAUCACUGAAUAUCUAUGUAUGAAUAUCCUAAGUUAUAAGUUAGUUUUAGUGGGUUUUCAAAUAGCCUUUUUUUUUUUUUUUAAAUAACUACAU
AAGUGCUUCUGUUGCUGGGUGAGAAUACUACUUUAUAUACAGUUGUUUGGUUUUCUUUUCCUAUUUGCUGGUUUAAUUGAUGUAUUACACCAAAAAU
GCAUUUUAUGUUCAUAAAUUUUAGGUUCACUUAGAAUAUAUUAUUUAAUAAGUUAAAAUUCUUUUGGCACACUAUUAAGUAAAAAAAAACUUUUAAA
AAAACUACCUUAUAUUAGAUGUUUAAUGUUCUUUGUCUAUGCUUUUUUAUUAUUUUAAAUAUACACUAUAUACAGUAUGGUGUAAAAGAGUGCCCUG
UGUAAAUAGACCUAUUUUGCAUUCCUUUCAGGAGUGGUUAUUGAUUCCUGACUGCAGAUAUCUAUUAUUAUUUGGGUAUCUGUGCUUGCAAUCUUAU
UGAAUGUAUUAUGAAGAAUGGAAUAGAAAUCAAACCUUAUUUUUGUACAGUUUUGAAGUUUUUACUUGGAACUGACCCACCUCCCCUUCCCAGUGGA
GAGCUGUACAGUGUGUAAAUCUGCCUUUACCUUGGAUUGGUACAGUAUUUUUGCAUCUGUGGGACCUACUUUUUUUGUUCUAGUAGUUUGAACUAUA
UAUAAACUGUACAAUCUGUAAAGUUUUUAUAGAAUAAAUAUUCAGCUGUGAAAACUGGUUAAAUAAAACUAAUAUUUCUUAACA

>*BRIX1*, NM_001031449
GCCUAGAAGUUACAGUUGGAGAUGCCAAAGUCAUCUUUUAGUCUUCUGUAGCUAUAUCAAAUAUAUACAUUUCUUAGCUAUAUUGACAGAAUAUUGU
GAAUACUUGUUUUCUACAGGUGAAACUAACUGGAUUUCAGUUCUAAGUAAACAAAGAUCAGUUUCUUUAUUGAAAAAAAAAAAAAAA

>*SOX10*, NM_204792
GGAAUGGGGAACAGUGACCAAAGCAGCGACGGAAAGGCUCCGAAGAAUCUGCACAGGCAGAAGAGCCUCCGAACUCCGAGGUCACUCAGUGCCACCA
UCCAGCCACCAGCACCCACUGAGUAUCUGGAAGUGCCUUUCUUGACCCCGGCUAUCGUUGGCUCACCCGCCUACAGAAAGGUUUUUCGUCCUUUCGC

CCUGUACCGAUUUCACGCAGGCCACACAACUGGCUUGCAACACCUUAUUGGCCUUCUAGAUGAGGAAGACUCAAGACAUGGAGUGACGGGUCCAUGG
UGGGCUUCGUUGUGCGCACCCUGGACUGUAAGAGGAGAGAGACUGUCCUUUCCUUCCCCUCCCCAACCUCCUCCAGAGAAUUAGCAAGUGUUUCCAA
AAGGCCACCAUGGCCAACUCUUUGUCAGCUGCUGCGGGUGAAGGGUGCUUGCUCAGCCUUGGUGAACAUGUCAUGGGAGGAGGAGUUGGGAGGACGG
CAUGGCCGGCUUCCUUGCGUCAGUGUUGUGCAGCAGUCCUGCCUAAGAUUCACACAUGCAUGAAUCUUGAUCCCUGCUGGAAGACCAACUUAUGCUU
GCUUCCUUGCUGAUUUUCACAGCUGCACGCUAUAGCUUUUCUCCCCGCCUCUUCCCCCUGUCCCAUCUGUUUAAUCCCACACCCCUUCCUGCAUGGA
UUUUAUAAGGCUUGACUGAACAAUGUGGAGGAUUCAGGAACUGCACUGUCAGCCUUAGCCUUUCUCAUUUCAUAGCCCGUAUGUGUGCAUGCGUAUU
UGUGUAAGGAGUUGAAGAGAGGUGGGGAAGGAUCUGUGGAUCAUUCAGGUGCCGUGGGUGAAUCCUCUCCUCUUACCAUUUAAUUUCUGCCGUUCAC
UUGUGUCUCAGAUUUCUGGCAUCUCAGUCCUUCCCAAUCUCUUCAGUAUCCCAACAAUCUCGCAGCAGACCAGGUCCUUGCUCCUCCAGCAUUUGAC
UGUGUCUGCUGAAAACGUGAAGGCACCAAGAAAGCUACCAUGCACUUGCCAACACCCAGAUGUGGUUCCAAUAUUCCAAUACUCACUUUGAACCCUG
CUCCAAACUCAGGCCGAUGGGGUGUACAUCACUGCUCGUUUGUUCAUCACAUUAUCGCCAUCUUCAGCACCUCCCUCCUACUUUCACUCAGGAUGGA
AAGAUACUCUCGUGGAUAUAAGCAAGGUAGAUAUGCACCCAAUAUUGGAUGACUACAAAGAAAAGAACACUGCUUACGUGCAGAAGAACUCAUUUUC
ACCUCUGGGUUUCAAGCUGGAAAGAAGCAGCUCUUGCAGUCAGAUCAACCCCUUUUGUUCAGCCCAUCUCCUGAGGACUACUUAAUUCUCCUUUGUC
ACCUCCAGGACUGGGUGGCGUGCCCUGCCAGGGAGUGAACUGGCCAAGAAGCUGCUGCAGGAAAAAGUGACUCCUGACAACUGAUCAGAGAGAUGUG
UUGUGGGACCUUCUAACGGGUAAUUCAGUCUAGAAAGAUAAAAUACUUCGACUGAACGUGGACUGUGAUUCGUGUCACUGUUUCCCUCUGUUGAUAU
GAUGUCAGAUGCCCUUCCCACCUCCCUUAGCUAUGGAUGACUUCUCCUCGUGGUGUCUGAAGAGAUCACGUAGAAUGUUUGUUCCCUGUACGCAUGU
AACCUAUUUGAGCCCUGUCCUCUUAUUUAAGGCAAGUAGCACAUUCCUUCCCCUGCUCCCUUUCCCCAGUCUCUAUGCUGUCCUAGCACCAUUAACU
CUGCAUUAAACAUAUGGAAUAAUAAACUUAAAAAAAAAAAAAAAAAAA

>*TEX14*, XM_015296110

UUAGAAUGGAUAGCGAAUACAUCUUAAUACAUCAGCUGCUUACUGCUGCUGGUACAGUAUUCAGCCUGUGAACUGCAGCCCUUUUUGGUCACAAAG
ACUUUUAAUGGUCAGACUUGUUUGAUCUGCAGCAGCAAGAACUGACAAAAUUGGAGUGCUUUACUUUAUAGUGCAGUUUAUACUGUUUUUAGCCACU
GUUUUCUAUCAGGUAUACUGCAAAGGUGAUCUGUUUUAUAUCAGUUUUACUGCAAAGGUGAUAUGUUUUCAGAAUAGAACAGCCUUAGUAAGCUGGC
UGUUUGUUUUUCUGUAUGCACACUUUCAGAACCAAAAUAAUUUUUUUUUUUUUUGCUCUUUUGCAUUACACAGUUUUCAGUGUUACAGACAAAAUGA
CUGUAAUUCUGUGAACUGCUGUUACUGCUCAGACUCAUCUAAUUUUUCCUUGCUGGAGUGUGGAGCGCUGUGUUGCAGCCCACGUGGUGUAUGUUUU
UCCUUGCUCAUUAAAUUUAACAAGGGUAAUUUCUUGUUUUCUCUUCUCUUAGUGCUUUUGUGAUCUCACGUUGAACUGAUUUGACUGAUAAAGGGGA
UUAAUUGCUGUGCAGAAAGUUAAUGAGAAAAAAGCAGUGUUCUUUCAGAGAUGCAACUGCACCAAUAUUUUCCCUGUCAGCAUUCAACGAGAGUAUA
GAAAAGUUAGCAUUACUGAGAUAGCUGUUGUUUAAUUAAACCACGCUUCUGUGGUCCAAAAAAGGUCCCUGGGAAGUAAUUGUGUUUUGGAUCAGGC
UUUUGAUGGGCACAGCGAGUUCAUGUUUAACUGUUGUGAUUAGCACUGCCCAGGUGCAGGAUGUGUAAAGAUGGUCCUUAUCUCUGUUGAACACAGG
GCAAUUCUAUGGGCAUUCUUACAGUUUUGUGUACCAUGUGUUUCUCUUCCAUCCUUGAUCAAGGAGGGAAAUUUUGCCUGUAAGUGUUAAUUUGGUU
UAAUGAUACUAAUAAAAAUGUCUAAGCUUAA

>*TBX6*, NM_001030367

GCAGGGAGGAACCUUCCCAAUGAACAGACUGAAAGAAAAUUGUAAAUGAGAACAGAUUUGUUCUGGGGUGCUAACCUUGAAGCCUUGUCUAUAGUAG
GCAAAGAGGCAUUGCUGCAGAUGGUGGCAUGGGACGCCUUCACUUCCUUGUCUCCUUGCACAACAACCCUCACGGUAUUUCACAAGCAGCAGCCACA
CUGCUGGCAGUCCCAGCUGUGUCAGGGCACGUCUGGCUCAAUCUGUGUGUUCUGCAGUGCUUCUUCUCCAGCAGGAAUAGGAAACCACAGGAACUGU
GAGAACUGCAGUGAAAGAGCUGCCGACCUCUGUCCCAGUCCAUGCUUGCAUUUGGCAGCUUCCAAAAUCCCUUCUUCCCAGGACACGUGUGCAAGGA
GAUAGGGGCUGAGGAGUUUCAGUAAGCCAGGCCAAGAAGCUGUAGAGGAUUUUUGCAAGGAUGAGCCAGAAAAGCAAUUCCAGUGACCCCUUGAGCA
GGUUUCAUCACUUAGUAUAGUCAAACUAAAAUCUUGCCUUCUUUUCCAGGAGCAUGUGACAGUAAAACAUGCAGUGGUACACGCGGCUGUGGAUCAU
GCAGGCAGGGAAGAGCUCGUCUUCUGCACGAGAACCAGCAGCAAGGCUCUGUGUUGNGUCUGUCCCUCAUGCCCACCCCUAUCUCAUCUCCCC

>*CCDC71L*, XM_015275786

GGCGGCGGGAGGGGAGGGAAGGCCGGCGCGGACAAAGAGACCAAUAUCAGUCACCUGUUUACAUUGCUUUUGUCUGGAUCAUGUUCUUCUGCUGCAC
UUUACGGCUCGCUAGAACCGGGGCCGGGGAGCCGGGCCCCGGGCGGGCGAGGGGCCGGCUGCGGGUACCGCGCGUGGGGACCAAGUUCCGCUUCCAC
UUCUCCCAUCGGGCUACCUCACUGGUCCAGAAGUCCACCCAAGAAGUUGUUUUCCAAAGGAACUUAACUUUCAUGCUUGUAUAAUAAAGCACCAUCC
GAUUCUCGUUCUCCCUUCCCCCCCCCCCCCCCCCCCCUCGCUUCCCCUUUCCGAUGGAUUAUGUAUGCUUUGUGGUGUUGCACUAGUAUGUGCUCAG
GGUAUUUUGAAUCCCAAGCAUUCCCAAGUUUCAGUUCACUCUGACCGAGGAUGCGUUAAGAAUGGAGGUUCAGGACUUGUGGCUGUUCUUGAUGGUG
CAAAAACUUUUUUUUUUUUUUUUAAUUUCAUGGGCUUAGUGAUAUAUACAGAUGUAUUGCUCAGUAUAAUUGUUACACUUUUGUAUCUAAAGUCAUUU
UUAAAGUUUUCUAGUUGAACUAAGUAUUUGUUUCUAUUGAGCACCUAAAGAUAGAAUCAUACUGAUAUUUUAUAAACCUGUUUACUUUAGGGAAGCU
CAUUUGGUCAACCUAAGUGAACUUAAUAAAAGUUAAAGAAAAGUGAUGUUUCAGUUCAAUGAAACUGGACUGCACAGAGAUGGGUUCCUCACUGGAG
UCUGCCGUGCUUGUGCUUAACGGAGCCUCUGUGGUAUCCCCAGUAAACAAGCUUAGAGCUGGUGAAGAGUGUAACUACUCGUGGAAGCUGGAUAUAG
GUGUCUGAAGGUGAACUGUGGUACACGUAAUGUUGGCAUUAAUGGCAAAAGAAAAAUUAAAAAAAAGACUCAGUACCUGGUUACUGCUUAACAAAAUA
GCCCUGGUGUCAACCCUGGGUCUGUCAGAUGACUAAUGGUAGAUAACCAUAUCAUUGUAAUUACUUGCACAUAAUUAUUUUUAUAAAUCUCUCCUGG
GCUUCAUAGUGGACUGUGGUGUAAAAUUCAUAAUGUGAUUUAACUACUUGGACAGAAAAAUUCCAUCUUGCUGUUCCGUGUGGCGGGCAGUCCCUCU
UUCAUCUUUAGUUUUUUUGUUUUUUGUAAAGGGCUUUUCCUGGUUCUUGCCCAUUGCUGUUGGUGUGUAAGAAGCAAAGGUGUAGAUUUUCUUCUGC
UUGAUGUUUUUUGAGUUUGGCAGAAGUGUUUUGUGAGAGACAGCUUGUGUUCUUUGUAUCCUAGCAAACAAGGAACUUACUGGUUUCCCUAGUGGAA
UGAAUAACUUCACAAUUUGUUUGUAGGAUGGUUUGGUGACUUAAAAUUUCUUUCCUGAUUGCUGUUGACUCCUUGUUGCCUGCUGUUGGUGGAGGUA
GUAAGGCAGCUAGAUGUGUAGAGGUAGUUUGUAAAAACUUUAAAGUACUAAUCUGAAGCAACCAAAUUCUUCUAAAACUUCUUUAUAUUAUGUAGUU
CUUACUUAGCAAAGCUGUGAAGUUCGCAUUCUUCCAAAGUAACCAUCUGUUCUAUCACCAUUAAGUUUUGAAAUCGUUUACAUAACAUUAGACUGCC
UGCUGAUGGCUGCAGUAGUGCCACGUCACAUUUCAGAGGGGAACUAGGAAAUGAAAGUUCAAGGGUGCAUAUUGUUGUUAUGCUAUUUCUCUGGCUA
UAAUGCCCGCUUAAAUUUAGAGGCAAAGUUGUCUUUAAAACUCAGUAUGACAAACUGCACUGUGACCAAAUGCCAAGUAUUUCAGUUUGACAUUCA
UUUCAGAAACGCUAGCUAAGAGGAUUGUUGCUGAUACUUGAAGUGUCAAGUUGACUUCAAUGAAGUUGUGUUCCUACUGCUAGGUUGAAUUGUUUUU
UUCAGUAUUCUUUUGAGAAAUCUGGGUAGAGAUAAUAAAAUUUAAGCAGCUGUCUUGUGCGGAUUUUUCACAUAUAUUGUAAACAGGUGUAGGACAG
ACUAGGUCAAAUUUCUGGGACAGAGUUAAGGCUGCCAGAGGUAGGAUAUGGGAGAUACUAUAGUUUGUGCUGCCUUGUUUUGAUGCUUAUUCUGUAG
UGUUCUUUGUGGGUACGAAAUACUGUGAAAUAACAUCAACUCUGUUGAGUGAGUCUUGUGCAGGCUGCAUGCCUAAGGUGUACUUAUUUUUCUCUUG
GUUCUGCCUUGACUUCUUUGUUUUGAGGCAGCAGCUCUUUGGGGGGUAUGCUAUGCUUUGCUGGUGCUCUGUGAGGAAGUUGGAUCGAAGGUCUGGC
AGCCGCGUUCACAAAUUCUUAACUGAGCUAUUUCUGUCUGUGAAGUCAGUUAAGGG..... (truncated)

>*DDX43*, XM_426195

UGACCGAUCCCUUGAAAUUACUAAAACAAAAGAACUGGAUUACUGAAUGUAAGCAAUGUUUGUGUUACUUCAAAAUACUGUACUUUCACACUGAUGU
GCCACAUCUGCCAGUGGCAUGCAGAAGCCUUUCACUGGAUCACUUAGUUACGUAUAACUAACACCACCACUCUUGAACCGUGCAUUUCUUCCUUAAG
CCAAAGUGGUGUUAACAAAGAUGUAAAGAGCAUCUUAGGAAAAAGACAUUUGAACGUCAGCUUUCUUUCUGAUAAAUUUCAUUCACGCUUUACACAC

GAGUUUCGGGUUCUGUCACACGUGUUGUUACAGGUCAUGAGAAUCUUUGGAAGUUGUAUUUGAAGCCACAAUACAAACGAUCUGAACUUAUGUUUCC
UUGUGUCAUAUUAAUAAGUGAAAAUCUAAUAAACUGAAUCUAAGUGAGA

>*GPR149*, NM_204662
AAGACAUUUAAUCUAAACAUUUAAACUGAUUCAGCAGACAUUGCUCCUCUUGACUCAAGUUACCCUAACAAAUACAAUGCCAACCGGUUUGUUUUUU
UUUUUUUCCUAGCAUAUAAAUGAUUUUUAUUUGGGUACACUGUUAAAUAGUUAACAUUUACAAAUGGGAAAACAAACGCUUCAGUGCUUUGCAGCUU
UGUGCAAUGAAAUCCAAGUUAUUUACCAAUCUCUUUAUUUGAGGAAGACUUGCAUCUGAAAGGAAUCCUAAAUGAACUCUUAAAAGCCACCCUUGCU
UGCUUGUUGGCACUGGGUGGUCUGUGGGAAGCCAGAAAAUGAGAGAGCAGCUCUACACCCGUGAAGAUGUAACAGGUUUCAAUGACUGUGGCAGUUC
UCCUACUGAUGUGCCAGACUUGAAACAUUUACUGAAAAUAUGUAGCACAAGCCUAAGACUAAGCCAUGCAUUUGGUUAGGGAUUGUAAGUUACGUAC
GUGGUUUAAUUUUCUACUGCUCCAUAUGUAAUUGAUACGUAAUGUCUCUGUAAGUGGCCAUUGUGUAGACCUUUGAAUGGGGGUGUCUGAAAUGUAA
GCUUGCACAUUUUAUAAGUCCAACACUGUGAAAAUGUGUUCUCAUGGCUGGACAUUUCUUUCAUCGAGUGUGUCAAAUGUUGGUUGAUCUUAUGCUG
GUUACUCUAAAGUGUGCCAUUUUCUGCCUCCUUGAUCUCUUAGGAGAGGGGACAGGGCUGCUCAAGGAGGCCAAACUUAGAGACCCAGCUUAGCUGU
UGACCUCAGCUUGAGGCUGAGUGCCCAGAUCAGCUUCCUGCCCUGGAGCACCCUCUGGAGGAGCCUGUUCUUAUGCUGCCUUACAGGAAGCGUAGAG
CCAAGUCUGAAAUCAAACUAUAACCCACCAAUAGACUGCUUCUUGUCAAAUUCAAACAGACUGAGGGUAAAACAGCAAAUGAAGCACUUAAGUCUAA
UGCUUUUUGUCUGAGAGCUCAUUUUCUGAGAUGUUGUCAUACAUUUCAGCCAGAUAGAAGUAGACUUCAGCAGGACUUUAAGAGGUUUGACUAAAAG
UUACACCACUUCUUUAAUUAAUAGAUUUAAUUUUCAGUGAGUGACAACAUAAUAAUUCAGGUGUCUGCAGUCACCUUGUUAUCCAUUCAGUGGGAAC
AUGCACAGGAAAGUAAGCACAGCCCUAUUUAGGAUUCCUUUAUGGAUUGUGAACAGGUAGAGCAAAAGGGCCAACUAGCUCAGCUUGUGUUCAGUAU
GGAGGCAUGGAAAGAACAGGUGAUUUGCAUUCUCACCUCACCAACAUAAAUCAGGCCUGCCACCAUGAUCAGACGGGCUAUGCUGCUGUGACAGUGA
UGGAGAGCUGAAGAGAAUCAGGGUCAGAGACUGCAGGCCGCUGUGAUGGAGAUGGAUCACUCUGUGCAAGAUGUGCUUUUCACGUGCUGCUCUUCCA
AGGUGAAAACAAAGGUUCUCAUAGGAUUGCCUCAAGGCUCUUAGUACCUGGUGAUGCUGCGUUACACUUCAGAUUUAUCAAGAAACUAUAGUAGACA
GAGCAAUCAAUGGUAAGUGUAUUAGUAGGUUAUAUCCAUGUGCAUGCAGCAGGGAGACACGUUCUGCUGUCUCUGUAUUACUUUGCUUUGUGUUUGU
AGGAUUAGCUCAGAAAGUUGCUUUAUAGCAACAGAUGUGCUCAAUCCAGAAAGACGUUGUGAGGGAGGAUGCAGUGCAUCACUGCCUCAGUUUGGUC
ACGUUCUCUGAUGGGUGCAUUUAGAUUGUGUUCAGGUUUGGAGGCAUUAAAAUUCUGCAUAGCUCUGACCCUGAGCUUGACCUAAUUGUAGAAUGUU
GCCUUGCUGGUGAAAAAGAAAUACAUUUUCUAUAAUGAAUCUGUAAAUCUGAUCAUGUACGAAUGUCUGACUGUUUUAUAUUUUAUUUUAGUGACUC
ACAUCAUUAAACAUUUUAAAGUGUCAAAAAAAAAAAAAAAAAAAAAAAAAAAAAAAA

>*PRPF39*, XM_003641432
UCAGAAGAGCGAGUGAAGUUCACUGUGCAGUAUCAACAAAGAGUAAAAUAUAUUUUUCUAAAUUAGGGUUGUGAAAGAACUGUUGUAAUACCUGCUU
UUUGGUCUUUUGCAUGUAAAAUGAGCUGAUGUUCACAGGGUAUGUGUGAAGCAAGAAUUGUGUGCUGGUAACUGAUAAAUGGUAUCUGUACAAUUCU
AGUUUACCAUAAAUGCAUUUUUUUUUAAAUUCCCUGCACUAACUUAUGGAGCUAUCUGAAGCAUUUUGUUUGGUCUCGUAAUGAUCCCUGCAGAGGG
CUGACCUGCUCUGGCUGCCCACCAAGCUGUUCCCUCAUUUCUCCUCAGGCCACAUGGAGAAAGUAGGAUUUCCCAAAAAAAAACCCAACACCUCAAU
GUGUUGAGGUGAGGACAGGGAGAUUGCUCACCCCUUAACACCAUGGGCAAAACAGACUCAGUGUGUGGAAGAUGAAUUCAUAACAGAGUAGGGCAGU
GAGAACUAAAAGCAAACUGAGAACACUUUCCUGCUGCUCCCCUUCUUCCCAGUUUCAACAUCUGUCUCAAUUCUUCUAUCUUCUCCCUUGCUAAGAG
CCACGGGAGCGAGGGUUGUGGUCAGGCUGUAGUAAUUUUUCACUGCUACUCCUCUCUCCUCACGCCCUUCUCUGCCCCAGGGUGGGUCCUUCCCUUG
GCUUGCCUUCUGGAGCUUCUCCCACAUGGGGUGCAGGUACUGAUCCAGCUGGAUGUUGUCUGGGCUGCAGCUCCUGCCUGACCUCCUGCAGCAGCCA
UGGGGCACAGCCACCUGCUCUGCGUGGUCCCGACAGGCUGCAGGGGUACGUCUGCUCCAGGGCACAGAGCACCUCCUGCCCCUGGCUGACCUUGGGG
GCUGCAGGGGGAUGCUACCCACUUUCUCUCUCAGCUUCUCCUUGCACAGCUGCUGUGCAGUGUGUUGAAGCCUUUCUUCACUGCACACUCAUAGCAG
CAUAGCUAAUGCAGCUUGCUGACUCUGCCUUGAAUGGAAGUGGCACUUGGGGCAACCCCUGCAGGCACCAUCCUGCCCCUUAAGCCCAAUACAACAC
CAUGGAUCUAAUCAGUAUACCUUUCUGUUGUUUUAGAAUAAGCAGUUUUGAUGUCUCCUAGCCCCAGCAGCUGCUCCAGGAGACGAGCAGCUCUUUG
ACCUGUUCUGCUCUCCUUCCCAACCAAAACUUCACCCAUUUCCAUUGUGCAAUCCUUUUCUAUGCCCCAGUGAGCAAAUCCCAGAUUACUUUGUUAU
AGGGUCAUGGCUGAAACUCUUCAGAUGGAGGUGGCAGCCUUUGGAAGCUGUGACAUGUAUUUUCUAUUCCAUGUGGGGGGAGGGGGGCUGUCAAAA
AUUAAAAUGCAGUGCAAAAUGAUGUUGCAUUCUUUUUUUAAGAAGCACAAACUGUGUUUGGGAUGAUUUUUGUAUCUAGAGUUUUUCAUGUACACAA
UGUGAGCAAACUUUUUUCAUUUUGAUCAAAGUGAUCAUUCUCAUUUUCGUAAUAAAAGCAAGGAAUUCAGCAGUUC

>*DMRT1*, NM_001101831
GGGGCCCACUGCUCCUUGCUGCCCAGGCAGCACUGCUGCUCCAUAUGCUGCCUGCGGCCACAGGGCUGGAGUCCCCUCUCCAGCAACUGCAUCAUGC
UGCUGGCGCAGCUAGUGAGAGGGAGGAAACCAGUGUUAGUCCGUCAUGUUUCAUUAUCUUCACUUGAAAAAUAAAAUUAGCUCUGAAUAGACCUAAA
AAAAAAA

>*PRPF19*, NM_001039331
CCUCGGGUGUGGGGCUGACAGCGCUCGCUGCAGCGCUGGGGUUUGGGGUGGGGGGUGGGGGGGUCGGGUGGGAGGGGUUUCCUUUCUCCUUUAGGUU
UCCUUGCUGUAACCGUUGGCAGAGGUGGGGGUUAGUUGGAUGUCGCCCUCCCCGUGCUUCUUGGGUUUGUUGUUUUAGUUUUCUUUCCCAGUUUCGU
GUUGGUCUUUCUGGCUUUAGGCUCUCUUCUGUGGCUGUAAGUGAUGCACUGCCAGGCCCCGACCCGUACCCCGGUGGAUAGUUGUGAUGGAGGGAUG
GGGGGCUGUGCGGUUUUGUAAGCAGUUAUCUAGUUUCAGUACACAAAUAAAGAACUAUUCACCGUAAAAAAAAAAAAAAA

>*MAPKAPK2*, XM_015299062
GGGCGCCCUCGCCGCCGGCCCUUGAGAAAGCAAUAACUAUAUAUAUAUUUUUUUUAAGAAAAAAAAAGACAAAAAGAGGCAGACUGUUAUAUCAAGC
GCAUGGAAUUCAGACAUUUAUACCAGACUAGUUAUUUUUAGCUACUCACCUGUGUUUCUGACCUUUCUGGAGCAGGCGAUGUCCCCAUCCGAUCCAC
ACUCUCCGACCGGGGGGGGUUUUGUCCUAUAGGUGAACGACGCUGAUAAUUGGAUUUUAUUUUAUUUUUUUUCCCUUUUGUGAAACAUUUUUGGUUU
UAUAUUUUUAUUGUGGUUUUUUUUUUGUUUUGUUCGUUUGUUUGUUUUCUUCCUUCCUUCCUCCAAAGACAUGGAAAUUCCUGUGGUGACCUUCCUA
UAGGGUAUAUAACAAAUACUUUUUAAAACUACUGUAGAGCUGAAACCCUGACAGCGUGCUCCCUGCGUGGAGCGCUGGCUAGAGGCAAGCUUCGGGC
AGGGCUCGGGGAAGGAGAGCUCCCAAAGCAGCCAUGGGGGGGGCUGGUGGGUGGUGUGCUGCCUCCCCGGCCUGGAUAUCUGCUGCUUUUAAAGCAC
GCUUGGGGAAAACAUGAGGAAAAGUUAUCUUUGGAGCCCUGCCCUGCCCUGGAAGCCUUGCUGCUCGCUGGGGGCACGGGGCUCUCCGCAAGCACCG
CGUGCGUCUGUGCUGCUGUCUGUCCGUACGUGGCUGUGCGUCGGUACGGGGCUCACCCCCACCCUGUGCCAUCCAGUUCUGACCUCAGGGCGGGCAG
GGGGCCAGGUUUGGGGGUGCCCCUCCCCUGGUCCCCCAAUCUGUGCAGGGCAAGGAUGCUUCCCCCUCCCCCCCACCCGAACGAUGCUGUGCUGGGG
GUCCCCGGGUGAUGGAAGGAGGAGGGGAUGCGGUGGUGCAGGGCUGGGGUCUUGCUGCCCUCCUAUGUGGUUCCUAAGGGGGGGCUGGGAGGGAAAC
CCCCAUCCCUGAGCAGAACGGCGGCGCUGAAGUCUUUAAUCCAUGAGUUCUUAUUAUUAUUUGUCACGAUGUUUCACCGCAUUUAAUGUGCUCGGGG
AGAAAGAAAAACGUUUUUUUAAGGCCAUGUUUAGUUUUUAGGUGAUCUUUUAGACACUGUAUGCAAUUUUAAUUUGUUUUAAGAAACAGUUUUUAUC
UUAUAUAUAUAUAUAUAUUUUUUUUUUUUUUCUCCCGUCUGUUGGCUUAUACUAACCUUUCUGGUCUGCUCUUCAGUCCUUUGGAUUAAAGACUCUU
ACUGCGUC

>*PRPF8*, XM_415805
CCCUGCCUCCCCGGCACUCUUCGUACUGUUGAUAUUCAACAUCCUCUCCCUGCUCUUGGUGGCCCCCUUCACGUGCUCGUGGCUCCGUUGCCCCCCG
UGCGUCCCAGCCGUGCUGUCCCGGUGGUUGUACCGUCUUAGAGUGGCUUAAUAAAUUUUUUACAAAGCCGAA

>*CCNA1*, XM_004938747
AGAUGGUGGAUCCUGGACUUAGGUUUAUUUUUGUGAUGAACAAAGCUGGUCUAACAUCUCAUAGAGCACUGCAGGUCAUGUGUCUGUAACCAUGGUG
AUCAGAACAGUUUUUAGCUAGUAUUUUUUAAUUGGAUGCCUUUUUAAAAAAAGGAUAUUAGAAUAUAGCUCUUAAUAUAAUUGGCAGCACUUUUAAU
AAAUGUCUUAUUACACUUCCUCUGAA

>*SRSF2*, NM_001001305
GAAAAUGAUGCAUCAGAUAAAUGAGAAUCUACAUAAAGGACGCCUUUGGGGGGAAAGGAUCGCUUGAGUCUACAGUCCAUUGGAGAGACCCCUGGCA
UGAGCAACCAGCUGUCGAAAAGGUUAUUUUGUAACAUUUUGUCUAACUUUUUACUUGUUUAAGUUGCGCCUCAAGUGGCAGAUUUUACAUUUUAUGU
GCCAUUUGUUGCUGUUAUUCAAAUUUCUUGUAAUUUAGUGAAGUGAACGACUACCGAUUUCAUUAUUGGCUUGGAUAUUUGAGGUAAAACUUCAUUU
CUGUUUAUAUAGUGCUGACUUUCAUUUGAAAUUUAACGGAUGGUAACUUGCUGCCUCAAGUUUCAUACUGAGGGAGUGCACAGCUCUUCUGUGCUGA
GUAGCUAACUGUCAGAAAGUAUCCUCAGUUUUGUCUUCAUUCCUUAAAUAAAAUUCUGAAGGAAAAACAAAAACAAAAAACCCUUCAAUUUGAAAUU
UACUGUAAAUGCUUUUUUACUUUGAAUUGAAAUAGUAUCUUCAAAUUGGUAGAAAUCUUUUCAGGUGACAGUUGAUAUUUGAAAGGAUUGUUUUUAU
CAGACUCAAUUCUAAUCUCUUCUCUUAUGUAUUUUUGUGCACUAGGCGCAGUUGUGUAGCAGUUGAGUAAUGCUGGUUAGCUGUUAAGGUGGCGUGU
UGCAGUGCAGAGUGCUUGGCUGUUUCCUGUUUUCUCCUGAUUGCUCCUGUGUAACGAUGCCUUGUCGUGCAGAAACAAAUGGCUGUCCAGUUAAUUA
AAAUGCCUGACAACUGCACUUCCAAUCACCCGGGCCUUGCAUAAAAAUAACGGAGCAUACAGUGAGCACAUCUAGCUGAUGGUAUACACACCUUAUU
UUUUCCAGAAUGGUAAAUCUUGCCAACAUGCAUAUAUGAACUUAUAUUUGAGAAAUGUAAUGUUAAGAAAGAAAUGGUUCAUUCCUCUGUAAGUACU
GAUUGGUGUAUCUUUUGCUUAAGACAUCUUGUACUAUACCCACUGUCUCUGUAGUUAAUAUUAAACAUUUUUUCUGUGUUAACUUUUCUCAACUUAA
UGUCUGAGUCAGCUUAUUUUUAGCUUGAAAAGAUUUCUGCAAUUAUACUGUCAAUGUUUAACUUCUAUAUAGUAACAGAAUGCAUUCUACUGCUUCU
UAACUUUAUCCACUGGGGUUGCUUUUAAAUUACUCUGGGCAGACUUGCUGGUAUUGUGACUAACCCAAAUGCUGUAUGAUAGUAUUGCACACCAGGC
UCCAAACACCAAACUGGGUCUGUUGGAUGUUAUGGAGGAUACACACUGAGCAGUACUAAUUGCAUUUGCUGUGUGCUUGUCUUGAUUCAACAGGCUG
CUGCUUAAUGGUAGCUCAUCUUAGUGAUCAUUCUUUUCUUGAUUUUAUUUUAUUUUUUUUUUGGAUACCAAAUGGAUUGUAAUGUCAAUCAUCACAU
GUCAAGGGUUAUUGCAAUGCCUUGUUAACUUUAUGCAUGUAUGUUAAAUGGAAAUGGAAACAUCUGAAUACUUGCAGCCUGGCUUGACCAGGGAUUA
AAAGUGCUUUUCUACAAACUGAGGAGUUUCUGUGUUCCAGGACUGAGUGUCUAGCAGCAGUCUACAUAGAUUCAAUUACAGGGGCUGGAUUUUGUAU
AAGAAUCUUAAGGGAAUGAGCUUCAGUCCUAGGAAACCAAUCUUAAGGGAUACGUUUAUCUUUAAAAGAGUUUGGUAACUUUUCUUUGUAGCCGAAG
GUUGUGAAUUUAAGUUGGAUCUUGUGGAUUGUCAGGUUGCUAUGUUGUGUAGUGGCUUACAUAAUGGAAAUGAGUCUCUUGAAACAUAACUUGGUCU
UUGGGAAGGAGAACAAAUUUCUCAAAAGCCAUAGUUACACAAAUAAACAGAUUUAUUUUUACAAUGCAUGUUUUAAGAAGUACUGCUAACGGGGAUC
UGCUUUCCAAAACAUACAUGAGGUUUGCUGUAAAUCAGACUGUUUCCUGCAGAUCCAUGCUAAAGUUAAUUAUAAUCUUGUAAUGGAACCAACGAGU
GGAAUAAACUUAAGUACCAUCUGAAAAAAAAAAAAAAAAAAAAAAAAAAAAAAA

>*EIF3E*, NM_001006349
CGCAAUUCUGUAGCGGGAAAAUUAUCAGUUUUCAUUUGACUAAAAAGCACAUGAUAAGUAUGUAACUUUAAGAAGGCAAUUGUUUCAUCUGAAUUAU
UUGAAUAAAAUUGGCUACUUUUAAAAAAAAAAAAAAAAAAAGAAAAAAAAAAAAAAAA

>*EIF4A2*, NM_204549
UCCCUGGAGAGGAGAUGGUUUGAAUGCAGUGCUCGCUGUUGCUGAAUAGGCGAUUCACUUGCAUUGUGCUUCUUUCUUUGGGGAUAUAUUGAAUCUU
GUCUCAAUGCUCAUAACGGAUCAGAAAUACAGAUUUUUGAUAAGCGAAGCGACUUUUUGUCGUGAGCUCUUGUGGGGAAAGCCAUUGGCUUUAUCCA
CUUUAGGGUUAGAAAUUUAUUUCCUAGUUCCAUAGAAGUGGUUGUAUUAGAUGUUCUUUAUCAUUUAAUAAUUUACUUAUGGACUAAAAGAUAUAAG
UGCUGUAUAAAAUCAGCCAAUUAUGUUAAACUAGCCUACCUUCCUUUAUUGUAUGUACUUACCCUUCAGAUUGAAUUGGAAAGGCCUUUCAAAAUCU
CAAAACUUUUAUAAGAGCAUUAAAAUGCAUUUUCGUUUGAUAUGUAUUUAUUCAAUAAAGUAUUUAAUUAGUGGUAAGUGUGAUCUGGACCCUGUUG
CUAAGCCCCAGCAAGCAAGCAAUCAUACUAUUGUCUUAGUUAGGGUUAAACCCAGUAAAAUUUGCCAUAUUGCACAUGUCUUAAUGAAGUUUGAAUG
UUCAAUAAAAUACUUCUAUAUUCACUU

>*BTG4*, NM_001278123
AUCUGUGGCUGUCCCUGUUCCAGCAGCCUCUAACACUGAGCUGUGCUGUGGGAUGAAGGCUGGUGCUACAGAGCUUCGAAAAGAGAACGUACUUUCU
GUCUGUUCCCUAUCCUCUUACUCUGUACUACUGAAACUUCUGGGUUUUUAAAUAACAUUUUAAUAUUUUAACAGUGUUAUCAAGACUGAAAAAUUAA
CGUGAGAUGCUACUUGUAAGCUAAAAAGGAAAUGCAUAUAUAUCAAUAAAUCUAAUCAUGUUUGCUUUUUAAAAGCUGUCCAAUCCCCUCUUGGUGC
CUGAAGGGAGUU

>*SRRM3*, XM_015296012
GCCCCCCCCGCCCCAACCAGGAGCCUCCAUCCCACCCCGGGAGCAGGGGAUUGCCAAGGAUGGACCGAGCGGGGGGGUGACCCUGAGCCUGUGGGGU
AUCUGGGGGUUGGGAGAUG

>*DDX49*, NM_001030938
AGCUCCUCUGUUCAGAGGACAGGAAAUCCCAGGCAGCCAAGAGAUUUGCUGUUGUCUAUUAUCAGGUUCUGGAUAUGCAGGUUUUCUUUUGAGCACA
UAAUUCAGAAUUUUUGUUCCUUUUUUAAAUAUUUGCCAUGGUCGUGGGGGUUUUGUUUGAGUUAUCUUCCCUACGUUAGCUCUGUAAGUGCACAUUU
CUGAACAUUUAGUACUGCUGAGUGUUGGGGCUGGAGGCUGAUAGGUGGGGGAAGGACUGCAAGCUACCAAAAGUGUGCAUGCUGCUGGGAUUCUGCU
GACUGAAGCAGGACUGCUUUUCUCUGUGUCAACUCAGUGCUUGGCUACUGAUUGCUCUGCUGUAUUUUUCUUUGACAGGAACUGGAGUACAUGGAUU
UUGAUGAGAAGAAAGAAAUAAAUAAGCGGAAACAAAUGAUCCUUGAAGGAAAGGAUCCAGAUCUGGAGGAAAAAAGGAAGGCAGAGCUGGCCAAGAU
CAAGAAGAAAAACAAUAAAUUCCGUGAGAAGGUCCAGCAGACACUGCAGGAAAAAAAGCAACUGCAGAUGAAGAGGAAACUACAGAAGAAAAUGGAG
UGGCGGAACAAACUGCAUGCUAAGGAAGAGAAAUAACAUCCUUGGCCUCCCAUUGAUGGAGGGAGUGUGGGAGCUCUGCUGGUUGGCUUGGACUGCC
CUGAUGUUCCUGCUCCUUUGGGAAUACUGGGAUCCAUGAGUUUUUAUGUGGACACUCUGACCUGAAACCAGUGUUGGAGAGUAUGGGGAACGUGGAC
GAGGAGAGAAGGGACGCUUUGAUAAACUGUCCCCAUUACUUUUCCUUUCUGUGCAGUGCUGCUGGCUUGGGCCUUUCCUGACAGGCUCAGCCAAUCU
CCUAAAAGAAUGUCUUUUUCAAGAGUAUGUUUCCUUCCUCUAGUUAAGGAGAGGCCAGACCAGAAGUCUUGGUGAUGCAAAGGGAAAUAAAGAUCCU
CUCCCUCUCUCUUCCCCUUCAUUCAGUCAUUUUAAUUUCCCUUUCAAGGGCUGGUACUUGCCACUCACAGUUGUUUAUAUCAACAGAUUCUCCCCUG
AGCCAGAGUCUAAAAAUAAUGUGACAGGAUGAGCUCAGCCUGUGUGUGUAUCAUAGUCCAGGUUUUUCUGAGAGGAAAAUUCCUAGAGCACAUGGAG
AGGUUGUGUUGUGUGGCUGGUCCUGGGGCAACCUUGUAAUAAUUCUGCUUUAUAUGUUUAAGUGGCACUCUUGCCUCUGAUUGUUUGCCUCAGCUCA
AGCCCAGCUUAUCCAUGGGGACAGCAGAGUCGCAGUUUGCUCAGUGACCCGGGCUGCUGGGGACUCCUGUUUCUACAGAUGAUCAGUUCAAAGCUCU

GUUCAGGUUUCACCCUUAGUGCCUUCUUUAAUGUUCAGAACUUCCUCUAAUCUGAGACUGAAGAACUGUUAUUUCUGUCCUCUGGCUUAAAAAAGUA
AUUUAUCUGGUCAGCAUCCAAAUAAAUAGUUCUGCAGCAAAAAAAAAAAAAAAA

>*EFTUD2*, NM_001031501
ACACAUCUCUGCUGAGCCCAGCGAGCUCAGCAAGCCCAGCACUUCCCCACCCCUCAGCGCUCCUCAGGUUGCUCUUGUAUGGCUGGAGAAACCUCUU
GGGGUGUUUGUGCACCGCGUGCUCAGCGCCCGCCCCGCACACAGCACAGCCCGGGGCACCAAUGCUUUGUGUGUUAUCAGAGCUCUGCGUGCAGUGA
GGAGGUUUUCCCUUUAGAUCCGCUCUCUGCAGGCACACAGGUGUAGUCUGGGCUGCAACGCAUCAGCUUGAGUCGAAUGUUUGGACUCUGGGGGUGU
GAGGUUGCUGGAUCAGCUCCCUGCUUUUGGUAAUGCUGCCCGUGGGUGCUCUGCCUUCCUUUGGGGCAGGUGCAGUGCUGGGUGCCGUUCCUACCGA
CCUCCCCAGGAAAAGGGGCAAGUCUGGAUUUUACCCUUUAUUUUUGUACAUGUUUUUGGAUCCCUGGUAAACAUCGAAUACAGACUUCCACCCAGAA
AAAAAAAAAAAAAA

>*SF3B1*, XM_421912
UCUUCUUGUUCUGUUGUUUGUGUUUAAUGCACAGCUGUUCUUCACAUAAAUGCUUCAGAUUUGAUGAUGUAAAAUUUUAAAAUAUUGGAGAUGAGUA
UAGAGCUGGGCAGAGGCAGAGGUAGGAAUCCAGUAGCAUGAUUUUUUAAAUAUGUCCUUGUUUUUUGAUGUUAAACAGUUAAAGCCAGUAGUGACCA
AGAAAACAGUGAUUACAGUAUACAGGAGGGAUUUCAUUUUUGGUUCAUCUUUAUGAAGAUUUAGAUUUCAUUCCUUGUGUUUAAAGGGGUUGUUUAU
UUGAGAAAUAAACAUUUGUGUAUAAAAUCUAAUUUGUGUGUGGUUUUUGGACUGAGAGACUUGUAAAACGAGUCUCUGAUGGAUCUAAGUAUUUGUU
CAUGUAAUAAAUAGGUGGAGUCAAGCAUUUUAACCCUUUUGUCAUCCCCUAGCUUUUGAAUAAAUAAGAAAAAUAUACAAACUGUA

>*SNRPD3*, NM_001007838
UUUUGGCCUUGACCAGUAUGCUUUUUUUUAUUAGAGGGGCUCUUAACUUGUCUACAUAUAUGUUGGUAACUUCUGUCUAAUAAAUGUUUCUGACAAA
GCCUUGUCUGCUGUUAUAUGACAGAACUCCUCUUAGACACACUAGAGUUCUGGCUUCUUUCUGUAGCUGGCAAAUACAUUGGAGGUUGAGUUUCAAA
GUGUGUUGUCUAGAAAACAGUGGACUUGAUAUCUGGAUUUGACUUCUUUGUGGUUUUAUGUGAUGCAUUGAGUUUCAAAAGAUCAUCUUGCUUGCUU
CUUGUUCAGUACUGCUGUUACUCAUCCUCUAAAGAAUUAGAAGGCAUUCUUGAUGAAGAAUUUUUAAAUGUUGUUUUAAAGUUGUGUUAGCAUUUCC
AUUUACAGAACAAACAGCCAAACUGAAGCAGCUUCUAGAGAAGCUUCAUAUGGAUCUAUAAUAAAUACAGGUCCAUCUUCAAAGCUGUAUAUUAAGC
UUGUAUUCUUGUAUUUCUUAUUUCUCUCUUUGGAUGCAGCAAAAACUUAUCAUUAGGAGCACACAACUAAUAUUUAGUAGACAGACCAUUUUAGCCU
GAGUCUGUACUUGCCUGGGGUGAUGUGAUUCUGGCUAUAUUGGCAGUUACUUGUUAUUUACCAUUGCCUGGGCAGAGAAGCCUGAAUAUGGAAGAAA
AAAAACAAUUUUCAAGCAUUGAUUUGAAAUGUUUCUUAUGAAUCUAAAAGAUGUCUAGGAAAAGUCACAGAAAUGGUUAUCUAUUAUGUAGGCUGGC
CUUUUGAACAGAACUUAGAUCUGACUUUAGCUUUUGUUAACGUCUGUACACAAUCACAUAGUUCACAAGCUCAUCUGUAGGAGGCCAAAUUUAGUUG
UAGCUUUAGCUUAUUUUUGCUUGAAGUACCACGUAGCUGUCUUACUGACAUUAAUGGUUGAAAUAUUUAAUUUGAACUCUCCUGUACCUAAUUGUAC
ACUUCCAUACAGUCAUCAGUCUAUACAUGUAUGAAUGAGUCCUGCUUUUCUGCUCUGCUGUAUAAAGACCCCCUGUGUGCAGACAUGGAAGUUGAAC
UAAUGUACUCAUUUGUUGAAUUGUCUGUAAAUAAAGCAGUGUUUGAUUAGCAAAAAAAAAAAAAAA

>*SNRPG*, NM_001302092
UGGCAGAGGAACCCCAUAGUGACCUGACCCUGUUGUGUUUUGUUUACUCACUCGAACCUAGUGAAGCAACCACAAGUGUAAGCUUAUUUUUUGUUAA
AUAAAUUUUGUACUGGUUGAUGUAA

>*HSD17B1*, NM_204837
GCGCGGCCCCGGCAAUAAAACCUCGUGCCCGCG

>*SNRPB2*, XM_419331
CCAGACACUCUUUGGAAUUCUUUGUAUUGAUUUUUUUUCCUUUUUUAAUAUGAGAGUACAGCAUGUUUUCUGUCCUGUGCAGAAAAGAUAAGACUGU
UGUGUGAAAUGGCUGCAGCUGUCAGCACAGUUGCAAGACUUCUGUGGAUCUACUGAAUAGUCACUUUUUGUGCUAAAAUACUAAUUUUUAUAAAAUA
AAUUCAGCUUUUGCUGUUCUGAAA

>*RNPC3*, XM_025153040
AACAUAGCAAAAGAAACAUUCCUGCGUAAAUACUGCAGUAAUACUGUCAUGCAGAGUGUAUCCUUUCUUGUUGUAUCCUUUUAUGUGCAAUGUUUCC
CAUAGCACUCAACCACCUCUAACUUCUCACUUAGAAUAGGUUUGGGGAGGAGCUUAUGCAUUUUAAAGCCUGAAUUAAUCUAUCAAUUUAGAAAGCA
GCACAUUUUCAGGGGGGAAAAAAAAAAGAGUUAUCUGUUGAGUGAACAGUUGCAAUGAACUUUCCUAAUCUUAAAAGGUAGCUUUCACCUUUCUCCU
CAAAGGUCAUAUAAUUUACUUCAUUUCUGAUACAGUUCCUUAGCUUAUCUGAGCUAGAAGUGUUUCACUGAUGUGUGUGGAGCUGUGCUGGGCUCAC
UCUGAGGAUGUCAUCUUUAACUAAAUCAUUAGAAUAAUAUGUUGGUUUAAAAUACGUCACAAAGCCAUGAUUAAUAUUAUCAAAGGUUGAUGUGUU
CUUAGAAUGUUUGAUUACUGUUGUCCUACUGUAUGAGAAACUGUUGAUUUUCAGUUUGUACAUUUGUCUUUUGUUUUUGAACAGAGCAAAAUCCAAC
AGUGUCUCACAAUGCAGUAAUAGAGUUCAUAACUGAACUAUAAGUCACUGUUAACGUUUUUGUUGUUUCUUUCAUCGUUCUCUUCACUGAGGUAGUG
CUGGUGAUGGUGCCUUUUUUUUUUUUUGUGGAUUUCGUAUUACAUUGUCAUUUUUCAUUUACUGUAAUAUAAAGCCAGUAUAGCCUGUACCUGCUAC
GUCAGGUUUUUUUUUUUUCAGAUACUCAGAAGUCUUAUAAUGUUAACCAUCUUUCAACUUAGUUGUCAAAAGAAAGCAUGUCACUGAUAGGAUCUGG
UUGUUCAUGCUGGUAACACGCCUUUGUGAUAUUCCAGGUUCCUUUUGCGUUUCAUCCCAUUCAAGAGUUCUCAAGCCUGGGGAGGAAGCAGUACAAG
CGUCAGGACUCUGGAACUGUGCGUAGAGCAUUUCUAAAGUACUUGAGGCAGAGUAUAAAUUUCCUUAUCUUAAAGAUUCCAAGUAUGUUUGUCAGAA
GGCUUACAUGAUAAAUAUAUGUUAGUUUGCUUUUGAAAUCUCUAAUAAAUUAUUUCCUUUCUCUUAA

>*EIF4G1*, XM_015291529
GCAGCUGUGAGGAGGAGGAGGAGGAGGAAGGGGGGAGAGCAUGGGGCACCCUGGGGUGUGACUCCAUCCCCUCCCAGCCCUGGCCCCCUGGACU
UGCUCAUGCCGGGGCCGAGGGACCUGCUGUGCCCCCAGCAGCAGCAUCCCCCUCUCUUUUUAUUUCUCCUCUCCCUCCUCCCUCCGCCCCGGUUCUG
UCGGCCAGGCCUGUACUAGUUUGUCACGAUGAUAAUAAAUGGAUGCUGAUGGAGGCGGCUGUCGCUGUGGGUCUGCUCCAGGCCCCUUCUGUCCCCC
CCCUCCCAGCACACUGUGUCACCGGGAGCGGGGGCUGAGCCCCCCAGGCUUAUGUCAUGGGGGAGGGGGGUGAGAUGGGGAGGACGUCUCCCCCCUC
UCCCCCCUCCUCUUCCUCCUCCCGCCUCCUUGCUGAAAUAUAGAGAGAUUAUAUAUAUAUAAACUUAUUAAAUUUGGGUUGGGGACAGGAGCGUGAU
UUCUCCCCCUCCCCUGUCCUCUCUCUAUUCCCCUAUCACUGCCUGGAGUCCUCCCUAUGGUUUUUUAUUUUAAAUAUCAAUCAUUCCCCCCAGCUCC
UGCUAUGACACCUGGGGAGGGUGCCGGGGGAGGAGGGGGGAGGGGCGGGGGGGAAAUGUGUGACCCUUUCUCUCUGGGCCAGGCUGAGUCCCCGUCC
UUCCCCCGUAGCCCCCAUCCUCCCCACCCCAAACGUUUAAGGCCACAAUUGGGCAAAGCGACGGGGCGGCCCCGCAUCCGCGGCCCCUGUAUUUAUA
GAGCACCGGAUGUGACGAGCCGCACGUGUAAUUAUUAAAACAAGGAUUCAAUAACUGG

>*CWC27*, XM_424748

AAUGAAUGUAAUGCUAGCCAGAGCUGCCUUGAAAAGUGACUACAAUAAAGCACUGGCUCUUUUGUAAGCUGUAGAUCCUAAAAAUAUCACUGGACAA
GUGUAAAAAGGAAUCUCUCAGGACCCUACUGUAUGGUUUUGAAUCCGGACUACCGUUUGUUUCUUAAGUUGUGCAUUAAACCGCUCUCAGUGCAUUC
AUUAUCCAAGUUCUGUAAAGCUUUGUGUGUAUUUAAUAAAACAACUGACUUUA

>*SPDYA*, XM_004940192
AAUGCACUGAACAAAGAAACAGAUGCUGUGCUUUGCAGAACAAUCUGUGUUGCACUGCAGGGCCAUAGAUGACUGUUAAAACAUUUCCCUUUAAAUA
GCAAGUAGGAGUUAAGAGUCUGAUAUGCAGUAGAAAUCAAAGCAGGAAUUACCUAGAAGUUACAAAACUUGCAAAACAUAGCUAUUUUGUAACUCUU
UAUUCUGAUACAACGCUCAGAUUAGCAGCUAACAGCAGCAGCAUCAAGAAAGUGGCGGUCGAAACUUUCUCAGCUUGGUGAGGAAUGCUAAUUGAAA
AACAAAACAAGUUACUAUUUCAAUAUUGAAUUUUUAGUUUAUUAUAAAUUUCACGAUUUUGUGUUAUAUGUGAAGUGCUAGUACAGAAGUUAUUAUU
UUUGAAAAUAAAAUAUUUUAAGAUAAAACAUUUCCAAUAAAAGCAUUUUCCCCAGUA

>*TEX11*, XM_025149940
UGAUAGCCACAACUGCAGAAAAGGAGGACAGCAGUUGGAAACAUCCUGCUGAAGGAAAGAGGCAUUUAGCUUUGCAAGGGCACAAGGGCACAGGAGC
AGUGGAAAAGCAAGCAGGAUCUCCCUCUUGUUGGCAGUUAAGUUAUGUAUGUUUCCGGAUGUGUUUAUUGAAUAAAUGUUACUGUUUCCCUCUAGGA
GAAUUUAAUUCUGUGGAGCUUAUGUAGCAGAAAUAAAUGCAUAAGUUUCUCUUGA

>*ZC3H13*, XM_025146798
GCAAAUGGCGACAGUGGCUCUUUGUAGUUGAUAUCCCACUACCUGUGUUGGUAUUGAUGUUUUAGAACUGGUUGGUUGGAAAGCAGCUGUAGAAGAU
GGAAGAAAAGACUUCAUUUGUAUGCAGAAUUCCUAAUUUUGGAUGUGAAUAAAUGUGGUAAAAAAGAAAGACUUCUGUGUGAAGUUCUCUGCAAGUU
UUAUAGUAUGCAUCUUACACUAACAAAAACCUGUUUUAUGGUGCAUCAGGUAUAAUAAUGUGAAGAUGUCUGGUUUUAGCAGUUUAUAACACUGCCU
GAUAAUAAACUGAAGAAAACUGUG

>*TDRKH*, XM_015280019
UGGCCUGACCUUGAGCUGUUUCUACCCCUACUGGCCGCAGCGUUGGAUGCUUUUUGAGAAGCGAAGUGCCUGCCCCAUAUCACAGCACCCCCCGCAU
CAACAGCGCCCGUUUCACUACGAGUUGCAUCUCUACACCGACUCGGAACUGCUUUCCAGCUGCCCUCUGUAACCACCGGAGGGGACAAACCCGGCCG
CGGUGUCCCCAAAUCUCCCCCGGGAUGCUUCGGCCCCCCUCUGGAAGCCACUGCCCUGCUGCGUUCCCAGGGCCGCUCCGUGCCUCGUUCUUCGUGU
UCCCCGUUGGGUCGGUGAUGCUGUGGUCACCGUGGGGGUGCCUUUCACAUUGUUGUCUCCGCGCCGUUGCGGUUUGGUUGUUGUUGUUUUCACCCCG
CUGAGGGGAAGAGCUGUUUGGUUUCCUGGUUGAAUAAAGCCCAGAGGGCACUGAAUGCA

>*SYF2*, NM_001257294
ACUGUGACAGCCAGCAGAGUGCAUGCUACAGCCCUCCUCUGCCUGUUGAAGUGCAGUGUGAUUUUGCAAACAAUUUUAAGCUUUGUCAGAGAAAUAC
UUUGUAGACCCCAUGAAACAAUGUGUAAGAUCACUAGAUGUUUCAUACAGGAGAUGUGCACAGUCUAUGCCUGCAGUUGUAUAUAAGCUUUACUUGC
UAAGCAGCCUGCUCCUCCAGUGUCAUGUUCUAAAUAAACUCUCACUCUGUUUUAAAGUG

>*CRNKL1*, XM_419315
GGUGUUGCCUUCCCCUGCUCGUCUUCUUGGGCAUUGUAUGCUAUUUUCAGUGGUGAUGAAUAAACAUUCUUGAAGGGUUUUGCUAUUUUUGACUUAA
GACAUCUUUGGUUUGAGAAACAGCACCAUGUUGGAGGUGAUUCUCUUGAUUUCCAUCUACUGAAAGGAAAUGUCAUGGAGCAAGACCUGCUUAUCCA
CACCAGUGAUCUGAGAACUGUGAGAAGUGGAUGGCAGCUUUUGUUUUUAGGGCAAGACCAACUGUUGUAAGGCUUCUGGAAAGCUUCACAGUAGUGC
UUAGGGAUGUUAAUAUACCAAGACAACAGAUCUACUUUCUUUAAUAAGAAAGAUGUCUUCAACCAUUCCUGAUUGAUAUUUUUCCAGUAAUUCUGUA
AAAUAUAUGUUGUUCAUGAGGGUAGUUGAAAACAAAAACCAGUUGGCUGCAACUUGUUCAACUUCCAACCAAAGUAAGCAGUUAUCCCAGAAAUCUG
AUCAGGAUGGACAAGAAUAGAAGCUGAAUACACUGAAACUAACCCAUGAAAGACAAACCUGUAGAAAUUGCUACCGCAGUUUGUCUGUAUUUUAAUA
CUUAAAUAUAUUGGCAUUAUAUUUUAUAGCAAAUAAUCUAUUGGGAAAUAAAGUAGAAGUGUAAAUGGACUUCUCAUUCUGCUUUUUCUACUUCAUA
GGGCAAAAAACAUUGAGGGGAAGUUUUCUCAUUUAUAUGCUCUUAGGGAGGCAUUUUUUUUUCUUUUUCCCCACUUUUUUUCAUUGUAAUGAGGAAA
AAAAAAGCUCUUGUUCCACUGCAGCUCCUGCCAGGAGAGACCCAGAAAAGUCAUGCAGGACUGCAUGGCUCUGGGUGGUGGUCAGGAGCACAGGGGA
GAGGAAAGGAAGAGUGUAUUUACCUGCAGCCUUACCAUUCUGGUGAGGGCAGAAGAAUGCAUGUGCUGGAGGUACGCCCACAAACAUGGGAGUACCA
GGAGAACAGAUUUGUGGGAAAGCUCUCUCAGUUCUCUUGGGUCAUUUGUUGGAGUGGGUGGCUAUCUGAAUUGCCUGCAGGACAAGUUCUAUCUGUG
CAAUCCUCUGCUUAAUCUGGAAAGACAUUCUAUCCCUGUUUUGUCCCGUGUUUAUUGAUCUUGUUAAUUACUUAGCUGGCUGACCAGAACAAAAAAA
AUUGUUAAUUGAGAGAUUUUGUCCUUGGGUUACUUGCUAAUCUUCACUCUUCUGAAUUAAAAAAAAAAAAAAAAAGAAAGAAA

>*PLRG1*, XM_420368
UGCAGCAACAUACUUGUGCUGUAAUUAUUUUCUGGCUUUUUUGAGAGCUUUCGGUCACAUUAUGGUCUGCCUACAACAGAAGAGCAGAAAGUCAGCA
AGUCUUUGCUUUUUCAGUGUGUUUUCUAAUAAAUUUUAUUUCUUUCUUUUUGUCUUUUUUUUUGGUUGUGUGAUCCCAGCAAACAGGUUGCAGCUGU
CAACCUUCUCUUUCUGGAGCAUGUAUAAUUCAUGAAGAGGAUAAAUGUGCAGGUGGUUACUUUUUAUAUGCAUCUACUGAGUGUGCAUCUGAAAAGA
UAUUGGUGAGCUGGGUUUUCUGAAAGACCCUUGCUUUGCUCCAGGAGUAACUGAAUUUACUCGAGUCCAUCUACAGGAAGCUUCUUGAGUGACACUU
AUAGAUUUUAGAGCAAUCUUGUUUACUGUGUAUGUGCUUAGUAAAUACUGUAAUGGAACAAUAUAUAUGCUAAAUUACAGAAUAAUAGAUUAACCAA
UAAAGAAAUGGGAUAAUACAAUAAUAAUGUAGUUUCCUUGGAAAACACCAUAUGGGCAAUCCCCAGUGGCUGAGUUAUUGAAUGAAAUUGAUUUUCC
GAGUGUUUCUUUCCCUAGGACAGCUUUCAUAUGCAACUUCGUAUCUUCGCUUAUUUAAUUUUGUAUAUUUCAUUUUGCAUAUGACCUGAUAUAAUGA
GAGCCACUGUCAAAUUAUAUUGCUUAGUUAGCACUGGGCUAGGACCAGUUGACUUUGUAAAUCAUGUCUUGCUUUUUUCAGUUAUCCAAAGUAUUUG
UGACAUUCAGUUACAGGGAACUUCUAAAACUGAGUUGCAGGGAACUUCUAAAACUGAGGUCUGAAAAAAAGCUCUUAAGUGAUUCCAGCCUUUAAUU
UUAUUUAUUUUUGUCUCCCUGUGAUCUUACAAAGGGUUUAAAGCAUGUAUAUGCAUGCAUGUAUGUAUGUACAAAUAUCCCCCUGUACUCAGCACUG
GUGAGGCCACACCUCAAGUAUUGUGUCCAGUAUUGGGCCCCUCACUGCAAGAAAGAGAUCGAGGCCCUGGAGCAUGUUCAGAGAAGGGCAGCAAAGC
UGCUAAGGGAUCUGGAGCACAGGCCCUAUGAGGAGUGGCUGAAGGAGCUGGGAUUAUUCAGUCCGGAGAAGAGGAGGCUCAGGGGAGACCUUAUUGC
UCUCUGUAACUACCUGAAGGGAAGUUGUAGUGAGCUGGGGGUCAGCCUCUUCUCGUGUGACUAGUGACAGGACUAGAGGGAAUGACUUCAAGCUGUG
CCAGGAAAGGUUCAGGCUGGACGUUAGGAAAUACUACUUCUCUGAAAGGGUGGUCAGGCACUGGAAUGGGCUGCCCAGAGAGGUGGUGGAGUCACCG
ACCCUGGUGGUGUUCAAGGAACAUUUGGACGUUGUGCUGUGGGACAUGGUUUACUGAAAAGUAUUGGCGAUGGGUGGAUGGUUGGACUGGGUGAUCC
UGAGGGUGUUUUCCAACCUUAGUGAUUCUGUGAUUCUAUGAUUCCAUGAAACCUUGAAAGUUUACAAAUUCUCCUGAAGGGGCCAAUAUGUCAUUGU
GUUUACAGUCAUGCAACUAGCAGUAACCAUGUAACGCUCUUUGCUACACCGUCUCUUUGGUGUCAGUAUUGUUCUUCAUCACCUAGUAAACUUUGUU
CUUCAGAUUGCUACACUUGAGAACAAAAUAGGAAAGGAUAGGAAUGAUGGUGUUCUUGGAAUUAUGAAAAAUAGGAAAGUGAUGUUUCAAGGGGAGA
UGGAGAGGAGAGAGUGAACUACAGUAAUGUGUGCAGUCCCUCUUUGACCUGCUUCUCUUUGGGUAUCUGCCUGGCCCGCAGUUUCUGGCUCAUGCAC
UGUUAGUUUUCUUGCUGUCCUAGAGCAUGGAAUAAAAUACACUGGUUAUUCUUCAUAAAAAAGUGAAUGAUAAUAGAGGAUAUUUACAGGUUGAAUC
AGACACCUUUUAUUUGGUUGCUAGUAUGACACUUUUUUUUAAUAUCAUAUUGCAUCUGUUUUCAUUUUAAUUAAUUUAAUAGGGGAAAAUGUAGAUU

AUGGCCUAUAGUUGUUUUGUACCUGUUGCUAAUGCUUUGUUCCAGAAAUCCAUGAAGGAUUUUGGUGUUAAAUAGACUUUUCUUGCUUGUAGUUAAU
UUUUUAUAGGAAAACAGUGUGGAGAGGAAUAACUUGGAAAAGGGUAUUUUUUUGUUGUUAUCUGUUUUUAGCAGAAGUGUCUGUUUUGAUAAUUUGG
GAGAUGGGAAAUUAGAGAUUUUUGCUUUGUUCUUGCACAUCUUUACUGGUAUCAUUUUUUCCACUGUUUGUCACUUAAAAAAGAGAAGAAAACUCCU
CUGCAGAACCUGUUGUUUUUCCUUAAAGGAAUCCACUAGCACAGGAGCAGUAAUUGCCACGAUGUUCUCCGUAGUGCUGUUGGUAAAGAUGCGGUUU
GGAAGUGAAAGGUUGUGUCACCACUGUUUUGAACUGUAUCUUUCCCUUUUGGCUCUGUAAUAUGAUGUUUCAGUUCUCAGUGUGACUUGUUUUUUUC
UCAGUAUAUUCUUUACCAUUUGAUUUGCAUGUUCCUCUCCUUUCCACCUGUUUUUUCUUCCACUAAUGUGAGUAAUGUCGUAUACUUCUGUAAAUAC
UAUCUGAGACAUCAAGAUUCAUUUAAAAAAAUAAAGGUCAUUUAAGUGCUUCUGUAGUGCAAAGUAAGCCGAGAUGGUGAGCUGAUGCACACAAAUC
UGAUUGUGUAGGAUGCACAGUGAGAUUAGAUUUGCCCACGUAGUCAUAAGGUAAAGCUAAAUGCGAUCCUUGGAAUGUAUUUUUCGUGCUUAUGGUA
AGUGCCAUGCUGCAAUGUCAGUUAAAGUAAUUUUGAAUUGGAGCCUCAUCUCAGAAAAUCAGC..... (truncated)

>*SRSF5*, NM_001031197
ACUAUAAGUGACUUGCCCUGGGGGCCCUUUUUUAAACCAUACUUGCUAAAACUCGUGGUAAGUAUGUGACUUUCUGAGGGGAAGGGUUUGGAUGGGG
UAGGGAGGGAGGAAGGGGGUGGGGGGGGUCGCGGAUCUUUGUAGAUGUGGACCAUGGUGGGGAGGGUUAUUGACUAAUUGUAUUAAAUGUUUUUUG
AUAACCCUUUUUUUGCUCACAUUUUUUGUGAAUGUCUGAAGUGAACAGUUGGUGUAUAUUGCCAGAGCUCUUUAUAACUAAAGCAGACAACCUAAAA
GUUUUUUGUUUUUUUUUUUUACUCCAAGAAGAGUAAUCUGGAUGCUGAAUUCCCAGUAUGCUCAACUGUGAAUAGCAAUUCAGAGUAUUAGUUUAAU
UUGAGAUAUUAAACUAACAGCUUUAGGCAUCUAAGAGCUCUACAUGUGCUGUACAUAAAGCUUUAUUUCAGAGCUUUUUUUUUUUUUGUGCUUUAGA
ACCACUGUUUCAGUCAUAGGUCAGCAGAUUAAGUGCUCAACAGUAUUGGUAAUAAAACUUUAUUUUAAAAAACCUUCUCAUUGAAUACGCCUUGAAC
UGCAGUAAGAAAAUGCCCAUUUCACUGCCCUGUGGUAAUAGUAUUCUCUGAGGCUCCCUAACUCUUGAUACUUAAGAAUUAAAGAGCUUAAAUAAAG
UUU

>*SRSF4*, XM_015297750
CCUUGCUGCAAGCAUACUUGGAACAGUCAGAGAAGUCUUUUGUACGUGUUUGGUAGUUGUAGCACAAGUGAUUGAAGUAGAAAACAUGUCAAUGCUG
UAUAUUUUUAACUGCCCUGAUGGUGUGUCUGUCGUUGUUAGUGGCAGUACUCACGCCCUGUAAUGCUCUCUGGUGCAAGGCUGUUCAGCUCUUUUAC
UAUUCUGUAGAAAGGUUCUCUUAGAAUACAGUUCUUCCUAGAUCACUGAAAUGGCCCUGUUUUCCAGGAAGAGCUUUGCAGAGAGUGAGCACUGCAC
CUUAGUGGGUUUAUGAGAUUGCAAUGAUGACUGAUAGGUAGGUAUGACGGCUUCAACAAUCUUUGCCCUUGAAUUGAUGCCCUUUGCUGUAUGCCAU
UUAGUGGAAGUGCUAAGUCUUACGUUUCAUACUACUUUUGUUUCAUAUUUUUGGACUUACAGAGUUGUGAAUAGCACAGUCAAAAGGGGAUACAAAA
GGUACUUGCAGUAAUCUGUGGGAAAAUAGAGUUCCAUAUUCUGGUAUUGUGACAAUAUCCUUGUUUUAUAUUAAUUUUUUUAUUUAUUUUUCCCCGU
CUUGCAAAGUACAGUGAUCCCUGUUUCCAUGAGAUUUGAAUAAAGACUAUUUUUGCUUGAUGACA

>*SRSF6*, NM_001030843
GACUAGAACUCUCCUCUUACCUUGCACACUAUUAUGGAACAUUUUUCCUACUUGUCAGGCCAUUAGUGUUAUGUUAGUUUUUCAGAAGUUGGUUUUU
UCUCUUGCAGGAGUGAAUGGCCUGUGAACUAAGUAAUGAGGCGUAAAGCUUUAAUUUGUAUCCUAAAUUUGAAAAUACCAGAUCAGAUGGUGUGGUA
CAAAGCAGGAAAAACCCCUUCCCCCCCCCCCCCCCCUUUUUCGGUUUUUUUUUUUAACAUUCAGGUAGAACUUUCAUUUUAUAGCAUUUCUGCUGGA
GUAACUUAACUACUCUAAGGUGCAGAGUGGUUUUUAUAUUAAAAGUUGAAUAUAACAGGCUUAAAUCUGGGCUUUUUUCCUUUUUUUUUUUUUUCCA
UGGUUUUAGAACUACAAGUAUCAUAGGCUUAGUAGGUUUACAGCUUGCUACCAAAGGCAAGAUUGUCUUGCUGGGCAGAUCAGGAUUCUCUGGUUUA
UCUUCAUUUGUGGAAGCAGGCUGCAAAGCUAUAAGCAAGGAACUAAACUAGAAAGGCUAGACCAGCAAUAAACUCAAGUUGGAACAUUUGUGUUACA
UCUUAACUUGCCAAAAGAACAUUCCUCCCCAAUACUGUGACUGUAUUAAAGGUAACAAAGGUACUCUGUAACCUGUGUCAUGCAGUAUUGCUACAAA
UCAUACUUUUGAAACUUAAAUGAAUGAAAGCUUUUUGAUGUUUUUUUUGCUUGACUAUCAAGUGGCAGUUCUCAGACACUAAUGUGUCUCUUGGAUG
UAGCUGAAGUCCACUAGUAUGCUGUAUGAUUUGUCAGCCUUGAAAUACCUAUUGAAUAUGAAAACUAGCUAAAAUAAAACUGUCUUCGCUCCCUUUA
GACUCAUUUAUGUUAACAUUCUACUUCGGAACUAAGAUUGUAUUGUGUUUGAACCUGAUCUUUACUGGAAGUGUAAUCUCUUCAAGCUAGAGUUCUA
GUCUUAAUGAAGGCUUUGCUAAACCACAGUUGCCAAUUCACUUAAAUUGUGGAAUAGAACUCAUCCUAAAAACUCCCCUUUAUAGCUAAAUUGAUUU
UUGUAACAUGCAACAUUAGAACUGCCUUGUACUCUUUAUACAGUGUGUAGUUUGACUUGUAUAAAAUUAAAUUGGUGACUCAGAAAAAAAAAAAAAA
AA

>*SF3A2*, NM_001004397
AGCCUCUCCAUCCCACCGGACUCGUCUGGCUCAUAGCUUUUUAUAUGCAGGUCACAACUGAUUGGGGUAAAAUAGUCUUGUUUUGUAUGCCAGUGAU
UUUGACUAGAUCCUGUAGAUCCAUUAAAAGGUUUUCACUUUUGCUUUC

>*DAZAP1*, NM_001031428
CGUGUGCUGGCAAUCAGGGAGUUCUGCCCACACGGGAUGCCUGGCACCAGCAGGACCCAGGGAUCCCAGACUUGUGAACUUGUGACAAUCACAUACU
UGAUGGAAGAAAAACCUAACACCAUUUUUUGGGGGGGGCGGGGGAGAUGGCUUCUGAAGGCAAAAGCUGUGGGUGUUUGGACUGCAGUUUUUAAAUA
UUUUCCUUUCUCUAACCCAUCAGCACAAAUAAAGAAAAUGUCACUGGUUCAAACUACAGGGUUUUAAAAAUGUCUUCAGCUUUAAUUCAAAACUUCA
GGUUUCUUUUUUUUUUUUUUUGAGAUUAUUUUUUUCCUGAGCCUUUGUUUUACCGUAUAUUGUAAACUUUUAUGUUUAAAAGAAAAAAAAAAAAUAUA
UACAUUUACAGAUUGUGAAAUUUUUAAGAGAAAUUUUCUACUAUGUAUGCUGGCUUAUUUUUUAAUUUAAAACAGGGUUUCCGUUAGCACUGAUGGA
AGUGAGGCUGAGCUGCAGCUCCCUCACUGCUGACGUCAGCGGUGGAGCAGAUGGGUCAGAAACUCUGGGAGUUAAAAAGAAAUCUACUGAGUGUAUU
UUGCAUUUGUUACCUGGUGGGAUAGAGCUGGUAACAAGGCAGGUGCAGACUUGCUGAUGCACAGAGUCACUUAACGAAGUGCAGCGUUGGAUCUGUG
CUUCGAACUCUUUCGAAUCCUCUGUGGAUUUCAGUAGGGGUUAACGGAGGCCUUGUUAAGAUGUAGAUUUCCCUCAGGCAAAAGGUUUAGGUGCAUU
UUGCACUAACCAGUGAUUACCCCUGGUUUGAAUAAAGAGAAGUACAUUUGGAUUAGAAAAAAAAAAAAAAAA

>*SNRPD1*, NM_001277998
CUUCUCCCAUGCCACUGUUAGCAAAUUCUGGGUGACUUUGGAUUCUUUUUGUACAGGUCUUGUUUAUGGUAUCCGUUUUUAAUAAACCGAGAAGUGA
AACAGGUGUCUUUCUUUUGUUAUUGAAGUAUGGGAUGGGAGGCAUGCAUAGAAAUGGGCUGCUGCAGACUAAGCGUGUCCUGUUGGUGUUGAUUAUG
ACUUUCAAGAUUUCAUCCACCCCUUGAAGGCCGUGAAAUGACCUCCUGCUCUUUGCCCCAUAGAGAUGGACUUGUGUUGACGCACUCUGAAAAUGUU
UGAAACAAAAUCCCUCUUUUAUAAGGCACUUGCCAUCAUCUAAUGGUUAAAUAAGGCACUGCAUUUAGAACCUGUGUUCCGUGAGCAUGUCUCUUGU
GUUCUUCAUUGACCUCUGUGGAAGUUCAUAGAUAAAAUGUUACUGUUUUCUAUUACAUGACGCUUAAAAUUUAAGCAUGGUGCUUCUAUAGAACAUU
AACACGGGCAAAUGAAACAGUUCUGUGGAACUGCUGGACUGUUGCAUCUCAUGAUUAAAAUUAAAGUACGCUUGGAUAUGAA

>*LSM10*, XM_004947764
GCCCCACUGCCUCCUCCAUGGGACACCGUGUUUUUCUCCAAUGAACAACCCAACUUCAAAGGGUCGGUGAUCUUCAGCUCCAUCCCUGUGCAACUGG
CAGCUACUCCUCUAUUCUCUUCUUACGGUUUCUCAGCUCUCAAAGAAAGCCCUUUUGGCCACAA

>*RPS20*, XM_428540
AUCGUAGUCAUCUACUUUGAAUAAAGCUGGCUUAAAAAUUUUC

>*TNPO1*, XM_424806
CCUCAUGCAUUGUAGCUGCAGUCCCAUAAUUAGAGGUCCUUCAGUCUGGGAGACUAUGAGGGAGCCUCUGCACCCAGGGAAAAUGUUACCCUUUACU
GGGGGGAAGGGUAAACCAGUAGGGAAUACAGUACAAUCCCAACCCUACUGGGAGGGGCGGGAGGGAGGUGUUGCCGUCACUGUAUUAAGUCGAUGUU
GGGAAAUGUUUUAACAUCUGGAGCCUUUGUGGGUGGAAAUCUGUCUCCUAUUACAACUCUGCACUGGAUGUGAAGAAGAAAAAAAAGAAUCUUUUCA
CAAAACAGCACAUUCUGGAAUAUUGUGGGGAUUUGUACCAAAAUAAGAACCAUAUAAUUGAACCAUAGGGAUACGUAAAGAGGAAAACUAUUUUGCG
CACAAUAAAGGAAACCUAAGAAUGGGGGUCACAAACAGUAAAAGGCUUUUUUUUUCUUUUUUUUUCUUUUCUUUUUUUUUUUUUUUUUUUAGUAAGG
AUUUUCUACAUUAUUUCAUCCUGCUUGAUGGGAUUCCUAGGAAUUUGCAUGUUAAUGAAGAACUACACAAAUGAAGUUAGUACAGUUAAAAUGCAGC
UUGUGUCUGUUUUAGGAACAGGCACUUGCAGUGUAUGAUAUAGAAACACCAAUUAAUUAGGGUUAACUUAGGCAAAAUGAAGCAACCUGCAAGCUGC
CAAAUGAGUCACUCCUUUCAUUUUUGGGGUAAAGGGAGGGACAGUUCAAAGGAGAGACUGACAUCUUAAUUUUUACAUUAAAAAGAAUUUAAGGUAG
UGGAUAUGAUUUGAUUAUUGUACUUCAUUAGAUUUAAUUUCUAGAGUAAGGCAUUAUUCUUAACGUGCAGUUUAAGGUUCAGGCAUGCGUUCUGGCU
CAUAGUGAUCAGAAGUAAUUUAAAUUAGUGGGAAAGUAGCAUGCUUGCAUCACAUAGAGUGAAAUUGGUAUACAUUUACCUAUGUUGCGCCAGUUUU
GUGUUGCAGUUUACCAAUUCAGUAUAGCCCUGCAUUUAAAGUUCCUUUUCUAAGAUUCUGUGGAUUUUAUUUUUAUUAAGAAUAUAGAUAUAAAGUA
CUGUAGUUAACAAUUAGGCCUUGAAAUACCUUUUAGGAUCUGUUAAGAAUAAGAUUGAUAUUGUAUUGUGUGUAACCUGCACAAUGUGGAAAGCUGA
UAUAGCUGUGCAAAAUACUUGCCUCUGUGCUGUCAGUGUGAUGUGCUUUCUGCAUGGUUAUAUACUAGUGAUUUUAUCAGAAUCUCUGAAAAUGAGU
UACAUGGUAAGACCUGUUAAAGGCUGCAUAAAUGUUAGUUGGCACGUAAAGACAGUUGUAGAAGUUGAUGACAUGAUUGCUAUAUUUGUGUUUAUUC
CCACUCAACAUAUUAUCUUCUAUGCUUUCUUUUUGUUCAAAGCAUGAUCUUAAUGAGAUGUUUAAGUUAAUGGAUGUAAUAAUGCAGGGUAUCUACA
CUGUAUCGGCGCAUGUUGGUGGCCCUUUGUGAUGUAGUUAAAUGCACAAGGGUGCAAGUUUAAAGCUACAGAGUGAAAGUUGGUUUGGAUCCUCUUC
AUUUCAUUUGUUUAGCUUUUCUGUUGUGUUUUUUUUUCUCUACUUACAUGUAUUCCUGUGAAUAAAUCCUUGUUAAGUUAACCCUUUACUUUUCCUU
CCAUGUGUAUUUUCUUAUGUACUGUGAAUGUGAAAUUCUAACUGGUACACUUGAUCUUGUGUCCAACUGAAAGUGGCAAGUCUUUAUUAAUCUAGAU
UGCCUAAAGAGUAUCCCAUGAUGAUAAAGGAAAAUGGAGAGAUUUUUAACUCUGCUGGUCAGAGGUGAAGCCACACCUUUCCAUUUUUCAAGUGCUG
CAUAAUUAAUCCGCAAAACAAAAUUAAGCCAUAACAGCCUUUUUAUAGAUUUAGUUUCUCACCUUUGCAUUGCAGAAUGGAUACUGGAUAACAGGUU
UUCUUUUGAUUUUUGUUUUCUUUUGUUCUUUUUUAAAAAUUGCUCUUCUUUGCAUGGUUCUAUUCUCUGAGUGCUGAAUGAUUUAGCUAUUGCACUG
AAGCUUGAGCUGUGUGAGUGUGAACUUAAAAAUUAUGUUUAAAGAGGAAACUUUUUUUUAAUGCAUGAUAGCAUGAACAUUUUACAUGCACCUCCGC
CCCCCUCCCUUCCCCCCCCCCCUCCACUUUGUUCUUUUUACGCUUGAAGAAUAGUUUGGACAUGUACUGUAUGAAGUACAUUCAGUGAUGCUAUUAC
GUAGUAUUUUUUGCAGAAUUGCAUGUGAUAAAUUGCUUUCAAAUUACAUUGCUUUGGUAGCAAAUACUAAAUUUCUUCUGAACUAAUAAUUGAAGUA
AAUUAGAAAUAGCUUUAGUCACUGAAAAUGAUUAAAAACCCAAUUCUAGAAGAAUAAUUUAGCAAGAUGGGAUAUUAUUCUAGACCCAAACUUGCUU
UAAUUUUGAGUGUUUAAGCAUUAGGUACGAAUUUCAGAGAAAUGGCAUUAAAAAUUUCAUAAAAUAAAAAAACAACUAAGUAAUAUAUAGCAUUACG
ACUGGAAUCUGAGUUUAGUUAAACUUCUUAAAUUUCAGAAAGCCCCUUUAACCAAAUGAAACCACCUCAUCUCAGAUUCAUUUAGAAUACCACUUUU
GUACAUACAUAUUUGAAUAGGUAUUUGUACAGAUGUGAACUGAUCUGGCAGAACAGCAAGUAAAGUAAUUAAAAUAAGGCAGAAAUUAACAUUACUG
GCAAAGUAGUCUUUUGUAUCAGCAAGUUCUCUCAUGGUAUGUUUUAAAGCAAAUAAACCAUAUUCCUUAUGCAUAAUAUAGUGCAGAAAUUCACAGA
AGCCAUUUAAGGUAUAAUGGGAGGUAAGAAAAGUUCCAAAUCUGCAUUAGCAGUUAUAAUU..... (truncated)

>*GTSF1*, XM_001234363
AGCAUCGUGAUCGUGUCACUGCAGCCUCAAGCCAUAAGCCGUCACUCUGUCGUGUGGGUGCAUGGUGUUUCAAUUCAUACAAUAAUGGGGAAUCCUC
UGUGAAUUCCUUACAACGUGCAAUUAUAACAUGCAACCCUUACAACAUGCAAUUAUGCAGUCCUUACUAUAUGCACGUACACAGCUGAAAUCUAGAC
UUAAGUACUUGUCUAGUUUCCAUUAGAGUUAUGCAGUUCUUAUUUGGAGAAAAGUUUUCCAAUGCUCAGAGCAUGUUUGAAAGCUCUAACAUAACUG
UUCUUUCCUGGGGUGGGAAAAAGUUUAGUUCACUAUCAGUACAUCUAGGACUGUGGAAUUGUUUGGAUUAAACAGUAUGUUUCUAAAAAUGAAUGCA
AAGUGCAAAAUAAAAUCUAAAGUACAAGCCCAAAAAACUAAGAAACAGCACAAGGUAUUUGUCUAGAAUAAAGUUAAUGCCACUGAUGUUUUGAAAA
GAGUGCUCCUCAAAAUACUGACUUCUUACCAUUAGUGUUACUGCCAGUGUAAAAUAAUGUAUGUUGACUAGCAUAAUGUGCAAAAUUAGGAUUGGUU
CUGUCAAGGCUCUUGUUGUUCAGAAUGACAGUUCAUAAUAAAAAGAUUUUGAAUCACA

>*RBM22*, NM_001006151
CACGUCGUGCCACCCUUGUCAUUUAAGUAAAUGUUAUUUUCUAGUUACCAUGGUAGUGCCGUAUGUUGAGCUGUGGGUGAACUAGAGAAGCCUUAUU
UCCCUGCUUGCAGACACUGGGCAAGCUGAGCUCUGUGUCACUGUUAGCUACUCAGAUCGUUAAGACAUCCCAGAUCUUUCAUUUAGUGCUUAGGGUU
GGGUGGGGUGGGGGGGCAGAAAGGACUGUCAGGUGGGUCUGGGGAUCAGAUGUAUCAGAAUUACAGUGCUGUUAGUGUAUCCCAUUUGCACACUUUU
GAAAUAAACUUCUGGAAAAAACAAAAAAAAAAAAAAAA

>*ACTL9*, XM_425897
GGGGACACCCCAAAGCCGGCAAGAAACAUUGGUGCAGUUUUAUCAUAGCUACAGACCAAGCAGCAGAAAGCAGUAAUAAAUAGUUUCGUACCACCUG
AA

>*ISY1*, XM_414311
CAUUGCACUAGGAUGUCCUGUUCUAUAAUCUAUCCUAAAGCAACAAAGUUUCUUGUAUUCUGGCAGCAACAAAUUUAAGCUCUGAAAGUCCUGUGAA
UAUAAAGAGUAAGUUGUGGUUAAGUUAGUGUUUUUUAUGGAAGGGAAAACACCCUUUUUGUUUUAUCUGCUAGGAUGACUUGUACUUUGGUGCUGAG
AAAGAUCAAGCCUGGUUUAACUUGGUCUCUGCAUCUUCAGUAAACAGUCUUCAGUCCUUUCACUUUGUGAAAUGGUCUGUGCAGUGGUGCCUCUUAC
AGGUAGAGCAGCAAGCCAAGUGACUUAUGAGCCGUGGGAGAAAUCUGAUGGUUCUGCCAUUCUGUAUCCUGACUGUAAGUGUACACGUUUUUCUCAU
GUUGAAUUUAGCUGACCUGAGUCCAGAAUUAACUUCCUCUUUGAUGCCUGGUUAGUUUACCUGAACAGCCUAGGACCUAUUUCAAAAUGACUCGAUG
GGCAGAUCUUAUGAUUGGCUUUGAAGGUGAUUUGCUUCUUUAUAUAAUGUGUAAUAUGUAUGAAUUUGUUGCCAUUUUUGUGGUUUAUAGCCAUGUU
UGUGUGAAUUGCCUGAAUGCUGGAUAUUUCAGCAGCUAUUGUAAAUACUAGCAUCUUUAUAAUACCUUGUUCAAGUAUUUUUAGUAAAAUUUUUGUA
CAGACUGA

>*CDC5L*, XM_420058
AGCACAAAAUUCCUUUCAUCACUGAUGUAAUGUUUUUCAAGUAUGCAGAUACUUCUCCAGCAGCUUUUGCCAUCACUUGCAGAAAGCAAGCGUAGUC
UGCUGUUUGCAUCUCAUCCACUUUUUUUCCAUUACUGGACUAUUUCUGAUGCUGUAGGUAUGAAUGGCUUCAUCAAGGAUUUAAAAAAAAAAAAAAG
GAUUGAAAUCUCCAACACUUCACUGUGGUAUGAUUUUAACCACGUCUUCAUUUACUAGGAUCAUCACAUUAUUCCUGGUGUUUUAUCAGUCUUAAGA
UCUGUAAGGUAGAUGCCUGCAGAGUAAUGUCUUUUGAAUAAACCUGGAAUGUCGAAAAUAA

>*LOC769176 (ACRL)*, XM_015285316

UCAGCUGCAUGACCCAGAGCAGGCUGCAACGCAUUGCGACCCUUUUCUUUGGGGUAAUGCCCAAUGGCAGCCCCAGUGCCAAAGCCCUGCUCUGUCC
CAUCCACCCUCCUCUCACUGCACACCCAGGUUUACUCAGUUGGACAAAUAAAGCUUAUUUUCACAG

>*ACR*, XM_003640318
UUAGCUGCAUGACCCAGAGCAGGCUGCAAUGCAUUGCGACCCUUUUCUUUGGGGUAAUGCCCAAUGGCAGCCCCAGUACCAAAGCCCUGCUCUGUCC
CAUCCACCCUCCUCUUGACUGCACACCCAGGUUUACUCAGUUGGACAAAUAAAGCUUAUUUUCACAGCUA

>*SNW1*, XM_421294
GUGCCUCUGUACCUCGAGUAGCAAAGAAAUGGACUACAAGCUUGGACUUCAUUUAAAAAAAAAAAAAGGCGUUUUUAAUAUCAGAGAGGUAUAUAGGG
UGAUGUCAUGUAAAGAAGCAGUUCAGAACUCUUGUGCUGUGUGGAGAUGCAUAUCCACACACUAGUUCCUCUAUGAAGGACAGCUACCUAUGAAGAG
UGGAAGAAAGGAGACCACUUGGAAAUAUUUGCCAGGGGAACUGUUCUACAGCACGUACUCUGACUUUGCUAAUUCCCUGUUGCCCAGCAUCUCAGGG
UAAGAAUGUAGCCCCUUCCCACCGCCACAGUUUUAUUAAGUAGCUGAAGCGUUUAUUUCAUUUGUUACGAGCUCUCCCACUGUAAAUAAUUACUGUC
UCAUGCUGUAAUAGAUGCUUGAGAGCUUCUACCCGUGUUUCUGUGUCGUUCCAUAGUUUAUUUGAACAAGGAAGGGAAGCAGCUGGAUUCUGUUUUA
AAUCCAAUACUCACAGUUUCUCCAUAAUCUUUAUUUCAGUUUCUUUACAUUUAGUGAAAUAAAGCCUCUUUCCACAGUUCAUUCACGUU

>*TDRD5*, XM_025153194
GAAGAGGUGAGAGAAGCAGAUGGAGCUGCUGCUGAGGGAAGGCUCCAUGGAGCAGUGAAAUAUUACCAUGCUGUCAUUCUUUUAAUUAAAAAGCACU
UAAUUGUUUGAAAAACUUGAGACAUCUUCUCAGACGUGCCCAGUGCCUGUAGCACAUGUUACUUUUAGUUGAGGAAAAUACUCUGUUUUUCUUGUAC
UGGUGUUGUACUGCUUUAUUGCUCUUUAAUGAUUCAGAAGAACA

>*RNF17*, XM_015279441
AAUCAUUUGUUCUCAAUGUCUGUACCAGUGGUUCUCAGACCAAAGUCUGUGAUUGUGUGUGAAUGUCCAUCCAGUCAAACGUGAAACACAUCAGAGG
GCAGCUUUGCAGUAUGUGUUAUCUACAGCGAAUACUGAAGGACUGCUUCGUUCUGGUUUUUGGAGGGCUGGAUGGUUUUGUUUAUUUCAACUCAAUG
UUGCAUAGUUCUUUUUCCUCCCAUUUAUUAAGCACUCUUUUCAUCUCUGCUUGUUUUCUCUAGUUCUUAAUCUUGAAAUAAGUGAAUUUAUUCUAAA
UCACUUUUGACCUUAAAGCCUUAAGUAUUGUUCUAACAACAGUUUUUGUGAGAGCAGAUGGAAGGAAUGCGCAGUAGUGAAGAAAUAGAUACAGAAC
UAAUGUUGGGCACUGUUGUUCUCAUUGUAAUAGGUGAAGGGAUGAUAAUGCUAUGGUGUGUAACUAGUUUAUAUUUCAGGCAUAUUUGAUGACAAUG
CUGCAGUACUGGAGUCUGGAACUAGGCAUGAACAGGUGGGAGAGGGGAACGUUGCUGCUGCUGCUGUUAAACAUUUGCAUUGUUGUAUUUUGGUUUC
AGUAUGAGAUGGAUCUGUUUUGUUCAUUGUGGCACCUCUCCCUCCUCCCUUAGCUGCAGCAUUCAGUUGAAAGUGUUAGAAUAAAGAGGAGCUUGCU
CAGCAUGAAA

>*TEKT3*, XM_015295251
GCUGGGAGCUGCCCCAGAACGUCUGCGGGAGAUGUUCUCAGCUUUGCUAGGAUGACUCAAAAAUCCACGACCACUCUUCCUGAUGAAAAUCCUGAGU
AGAAAUUAAAUGUUUGCUUUGUGUGUUA

>*NR6A1*, XM_015279582
GCCCAGCUUGCCCCCGGCCCGUGCCUUACUCUGUGCCUCAGGCAGGGAGCGGGCUCACCUCCAGAGGAUGGGGGAAACAUGAUCAAGGCAGGAGGGA
AGUGCUGAGACUGGCAGCGGGUGUCCCUGCCAUUGUAGCAAGAGUCUUUUGUUUGUUUUUUGUUUUGUUUUGUUUUUUUUUUUACUCUUUUUCCUUA
UAUAUUUAUUUCACGACAGAGUUGAAUGUAUGAUCUUCAACACGAUGCACAUGGUUCUGUAUGAAUGCAGCAGAUGCAUUCUCCAGCAGUUUACAGA
AUGUGAAGAUGUUUAAAGUUGCAGUGUUUGUUAGGGUUAGUUCUUUUGUAUUUUGGGGCUGGGGACUGAGAUGAUUAAGACUACCUCAAGGAGAAGA
UGCUGUUCGCACACUGCUCUUUUCAUGAUUUGUAUCCAGAAGUGUUUGUCAUAGAAUGCAAACUGCCUCACCUCAUUGACAGAGCUGUCAGUAUCCC
AAAUGGACAUUUUUGGAAAAGGGGUGUUAGAGAAGUGCCAAGCUUUUUCUGUUUGUUUAAAACCUAAAAAAAAAAACCCUAAAAAAAAAAAAAACCCU
AAAAAAAAACCUUAAAGAAAAAAAGAGCUAAAAAAAAGAAAAGACAGGGAGCGGAGAAGAAGGGAAGAAGGGAAAGUCUGUCUGGCCAGUAGGUUUCC
UUUCCUCCAGCAGUUUCCAGGUGCAGGUGGUAUUGCCUGCGGUUUUCUUGAAUCCUGCCACCAGAUCUGCAGGGCUCCCAAGCCAAGAAGGGCUCUC
ACCCCCAUUCCCGGCUCGAUCUGUGGGAUGGUGAGCUGCCCUCCUUCCCUGACCUCCCCAGCUGGGCUGCCACUGUUGCUGCAACACCUUUCUGUUU
GAUUGAAACUGUGCUGUUGCAGUGUAGCAGGCUCCCAGCAUGGCAGCAGUAAGGUACUCUUGCUGAGAGAGUGAGAGCUGUAAAUAAUUUCUAAUUG
CUUCCUUAACAUCCAUUUUAGGUUCAUAAAAACAGGAAAAGCCUACUGAGGGAAGAGGCAGGGCUGCCCCUUUUAGUGUAUUCCCCCAAGAUGUCAU
UGACUACUGAGGAGACUUCUGCAUUGCAGCCAUCAGAAGCGGCUCAUUUCUGGGAACUGCUAACUUAUCCUGCAGUGAAAUGCAGCCUGCAUAAUGG
UGAGAAAGGACUCCAGGAUCCUCUGGUAUGGCUUUCUCUGCUGGACUCGGGGAGUGCAUGAUCCAUAGUUGUAUCCUGCCAGGACAGUUUUAUUGUU
UAGUGGAAUCCCUUCUUACCAGGAAAAAUUGGUAUGACUACAGAACUUGGUGAGCUGUUUGUCCCAGGUGUUGCAAGGCUUGAGUUUUCCAUCUAGC
AACCUCCACACCACUUCAGUGCGUGGAGGAAUGGCUUAAUGACAGCCAGUUAGCUGUCUGAGUAAUUGCUCAAUCCUGGCGUUUGUCUCAGUUUGAA
UAUCCCGUGGGUAAUCUCACGGUGUCCUUCCCAUAGUGGUGGUGGGGAGUUGAGGGCAGGUGCUCUUAUUCUGAUUUCCUCCAUUAGCUUAGGAAGA
CACAGUCAUCCUAGGGCCGUGCUACCCUGGCUGGACUCCUUUCCCUGUGUCCUUACUGUGAUCUUUUGAAUGCUGUUUUUAAAAUACUUUGGGAAAC
AACUUCUGUUUGCUCAGGGCAGGAGGAUCCAGUUGCUCCCUUUCUGUCUUGUCCUGAUAACAGCUGUAUAUUGCUGAGAAGUACUGACGCUGCUAUU
AGAUGAAGAUGAAUGUGACGGUGAAUGCGAGAAAAAUGUUGUUCACCAAAGUCAUCUUUCCUUCCCUGCACUCAUUCAGUUGAAGCCCAAGAGCCCU
UGUGAGACUGGCUUGGCACUCUCUGGGUUAGUCAGCAGCGAUGCGGGGCACCGGGUGCUGCCUGGAAGCCCUGGCUGCUCAGUGGGGCUGCCCCUCG
GAGGCGUGCUGCUGGCUAACAGAAGGCCCUGGGCAGCACUGUGUUCUUUGGAAUUCUGCCUGGGGAUCUUCCAGCUCGAUGGUUUCCCUUUUCUAAA
AGUCCACUCGCUGUUCAUUUUUCACUAUUUGAAUACGAUGUUACGGGUAACAGCUGCUGGGACCAGCAGCAGUUCAGUCUGAUAGAGCUUCACAAAU
CCAAAGUUGGUUCUGUAAAUCUCAUUUACAAUUUCUGACUGUAGAAUGAGACAAGCACAUAUUUCUCAGGAAGGCCUUCUGCAUACGUCGUAUACCU
CAAGUGUAAAUGGGAAGAUUUUAGUAUAUCUGUUUGUAAGUUAGAAAGUAUUUCUUCGCUUUUAACUUUACUUUCCAAAUAGUUUGAUAUGAUCUUU
UUAUUACGGGUUAUCUUCUGACUUGCUGUUCUAUACACUCAAAGAAUUGUUAAAUCGAAUAGGAAUAUUUUAUUCUGCAAUCACCAAACUUGUGUAA
UCUGCUGAUGAAUGAGAGUUAGUUUUAAUAAGUGUACAUAAAAGCAGUCAUCUUAUUGCCAAACACCCCACAAAGCCAAUCUGUUUGUUAUUCUGAG
UUUAUCCAAAGUGCUCCCGAGACUCAUGCUGUACCAAUAAUGAAACCCUUAAUUUCAUUAGGAUGACAAAUGGACAACACGUGUUCUUUCUGUUGUA
UACACCAAAUUUUGUAAAGGUCGAUUGGCAGCAUUUACUGUAGGGAGUAGAAGAUCCUUACUAAACUGACAAGUAAGGGAGAAGCUAAUAUACCAAA
CCAGAACGUAACUCACUGGACAGUCAUUCUUAAUACCUCCUGCACCGAGUCCUCUAAUGACUUGUAUUUUUUAAACAGGGUAAAGUGAAUGUGUUGC
UUUGCAAACUCAGGUAUUAUGAGAAGGUAGGAAUGUAGCUAGAAAUGUAGAGACGUUA..... (truncated)

>*MOS*, NM_001031516 (3’UTR is not known)

>*SUPT20H*, NM_001006275
AAGGAAAAGAGGCACACCUGCCCCUCCAAAAUCCUGAGACUUGCUUUUUUUUUCUCCCUCAGUAUAAAAAUGAUUUGAAGGUGAUUCUCUUUUUCCC
CUGUUCAAAAAGGAUAAAGCUUAAAAACAAAUCAGUGUUUUUAUAGUACUGUUGGGAAACUGAACUUUUGACAUUACACUGAAAAAAGAUGAACUUG

GAAGCCAACCCUACGUAACAGUGAGUUCAUCAGUAAAGCAGCUGGGGUCUGGCUUCCUUAAGUCACUUAAUUUUUUAACAGAAUGUUUUAUCUUGUA
UUUUUUACCAUUCAGCAGCAUUGUACAUAGUAAAAGGGUGAAAGACAUUUUAGAAGAAAAUGAACUUUGUAAAUGUAGUAUUAGUAUUUGUUUAUUU
AGUAUAAAAAGGUUUGUGAACACCGUAACAAAUACAAUUUUUAUAAAUCCAAAAAAAAAAAAAAA

>*NAA38*, XM_025142148
CUGGUGAUGGACCCCAGCCAUGCAAAAUGACCACCGGAAUUGCAAUCCCCUCAGCCCCAGCAGAGCCCCCGUCGUGGCCGCACUGUCACCACGCGAU
GCCGUCAUUCCAGUCCCCUGCAAAACAGGACACAGAUGCCCCUUCCUCCCCCAAACACUCCGUUUUCAGCCUGAAACAGAGUUGUGCAGGUUAAUUU
AAUCUCAGGAACAAAAUGUUUCAGUGGGCGAGAGGGGCGGAAGGAACACACAGCAGUUCUGGUCCCUCCUAACUGCGAUAAAAUGCUGCGGCCAAGG
GGGGUCAGGGAGGAACAGGACUGAAGGGGGGAGGUUGCACAGCGGGUCUGGGGCAGCCCCAGGGUUCCACAGGGCAGCCACCGUGGUGGGGAGGAGU
GGGAGGGGGCCGCUGAUGGGGCUGUCCCCCCAGGUUGCAGUGACACUGAGACACAGCCCCCCAGCAAGGACACCCCAGGGACCUGCACCGGGAGUCG
GGAACCCCAGAGCCGCCCUCCCCCCCAGUAAAGCAGAUCAUUUACAAAAUACAGAUCCCCAAAAUGCCCCCCAGGGACAGUUUUCGGUUGAAAUCUG
GGAAGGGUCUGUCCCAGCUCCCCCCGCCUCCAAGGUCCCUCUUAUUGCUUUGUGGGUAGGGAUGGUGGCAGAACCCACCCCCAGAGCCCCCCUGUGU
UUUGGGGGCUGCGAGACCCCCUUCACAUUGUCGGGAAAUGGGGGCACUGAACCCUCCCCCUUGCACAAGAUGAGGGUCACUGGGGACCCCCCGCACG
GUGGGGUCCCCAGUUUCCCUCUCUACUCUGCUCUUGUGAGGCCCCAUCUGGAGUACUGUAUCCAGGUAUGGAGCCCCCAGGACAAGAAAGACAGAGA
GCUGUUGGAGAGGGUCCAGAGGAGGGCCACGAAGAUGAUCGGGGGCUGGAGCACCUCCCCUACAAAGACAGGCUGAGGGAGCUGGGCUUAUUGAGCC
UGGAGAAGAGAAAGCUGCAGGGUGACCUCAUUGCAGCCUUUCAGUACCUGAAAGGAGCCUAUAAACAAGAAGGGAGUAAACUCUUUGAAAGGGUAGG
UAACAGCAGGACAAGGGGGAGCGGUUUUAAGUUGAAAGAGGGAAGAUUUAGGUUGGAUGUUAGGGGGAAGUUCUUUACCAGGAGAGCGGUGAGGUGC
UGGAACAGCUGCCCAGAAAGGUUGUAGAUGCGCCGUCCCUGGAGGUGUUGAAGGCUGGGUUGGAUGGGGCCCUGGGCAGCCUGGUCUAGUAAAUGGG
GAGGUUGGUGGAGAUUCGUGAUCCUUGAGGUCCCUUCCAACCCAGGCCAUUCUGUGUUUCUGUGGGGGGCAACAAAAGCUUCUGCGAUGAGCGGGAA
GAGUUGAAGACUGAUUUCUGUUAAUCACAAAAGGUCUGUGUGCUUGUGAAGAGAUAAAUUAACUUAUUUAUCACUUUA

>*FUT4*, NM_001031487 (3’UTR is not known)

>*SF3B3*, XM_001232347
GCAGCAGCUGGCAGGAGCAGAGCUUCUUGCUGGGCUCCUAUGGACACUCUUCCAAGCACAGUUGGAAGGAGGAAUGUGUGUUUUAUUUCCCUUUUUC
UAAAUACUGAUUUGCUCCUCUUGGUUUAUCUUUCAAAGUAAUGCGAUUGCUCCGGUAAAUACCAGUAUCAGAUAGUAGGUCUCCCAGCACAUCCCAC
UGCUUAUGUGAAGGCUCGUGCUAUCCUGGGUUCCAGCCAGGGAAUCUCUCUUGGGAAUUACCCAAGGAAGGAUGUGAUCUGACCCUUGGCCCCAUCC
CUGGAUCAGCUCCUCAGUGUCCUUCACCCUUAAGGCCGAGGGAGGCCAAGGGUGACUUGUCCUGGCCGUGCAGAAAGCACAAGGGGGUGGAUCGGGC
UGAGCUUCUGUAGGACCAGAUGGGUUCUUCCCAUCUUUCUGGUAAAGGAAACAGCUUUGUCCUGACGGAGAGCUGCUGGGUUCCUUUCAAUAGUGGG
AGGGCAGAGGUUUAGGGUUCAAGCAGCCUGGGGGAGUCGGGCUGGGGGAGGGUGCACUGCCAUGCAUGUCCCCAGUGUUGCCUUUUGAACAGCGAGG
GAGUUUUGGGCUCACCAUGUCUGACUGUUUUCUCUGUAAAUAAGAGUUUUGUACAAUGUUGUCUCUUGGGGGCAGGGAAGAGCAAGGCCUGAGUCUG
GGACUUGAUUUGUUUCAUAAUAACUUUGGCAAGAGCAGUGUCUCCAAGCUGUGACCGAGUAGCACAUGAAGAAUAAAAGCCUGUUUUUUAACUA

>*HOXB4*, NM_205293
CUAUUCCCUGGAGGAUUUCAGGGCCCGUUGUCGUAUGGCAGUGCCGGAGGUGGGGGUGAAUUUUUGCUGCUGCAAAUCUCCGCAGACCGUCUGUUGG
GGAAAAAGCACCCGGAGUUCGACCAGAAUCUUCAGGGGAAAUAAUAAUACUAAUAAU

>*RAN*, NM_205258
GGGAUGAAGCUGGAGCCCAGCGUCAGAAGUCUAGUUUUAUAGGCAACUGUCCUGUGAUGUCAGUGGUGCAGCGUGUUUGCCACUUUAUUAUCUAGCU
GAGCAGAACAUGUGCUUAAUCUUUGGGAUGCUGAAAGAGAUGAAUGGGCUUCGGAGUGAAUGUGGCAGUUCAAAACAAAAAAACACAACAAAACUUC
AUAAUUUUGGACCUGCAUAUUUAGCUGUUUUUUGGACUGCAAUUACUUCCCCUUUGAGUUUCAAAUAUAAGACUGCUGCAGUCACAUCAAAGUGUUA
UGUGGUAAAUCUUGUUUCUGUCAUUCCCAUUCUUUUUUUUUGUUUAGAUAAUAAAGUUGUAUUUCAAAUAUCUCUAA

>*WDR31*, XM_004945764
CUGUAGGAUCCUUCCCAGCACAGAUUGUCCGUGGGGAUCUGAUCCCCAAAUUCUUCCACUGACAGCAGGCACUGGGUGACCUCACGCCUGCAGAGCA
GUGAGCUGCAGUGCACAGGGGAUACAUUGCUUACUGGUUGCUUGUGGGUGGUGGGAUGCAGUUAGAGCUGGCUAGAAGAAAAGCUGAACCAGUGUUG
CAUUCAGGCCAAAUUCUCACUCCUGAAGAGUGCUGUGGGCUGAAGUCACAAAGCUUCCACACAACCUGACUGUAAAGGGAGCCAGCAGAGGAGAAAU
GAGCUAUAAGAAAGCACGUGCCAUCUCUGUGCUGUUUCUCAGGUCUGAGUGUCUCAUCAGAGAACUCCUCUUGCACCAGGAAAGCUGCUUUUACUGU
GAAAAGAAGGAACAACCAAGCUGGGACUUCACUACUGUGAGAAGAAACCAGGGAUCAAUGUCUCUCCCUGGAUCAGAUUUAAAUCAGAUUGCUGAGC
CAGCUCACAGCACAGUGCCUCCAGAGGACUGCAGGGGAUGAACAGUGUACAUUUACCUGCUGUGACGACUGGCUCCCAGUUCUGCAGCAGAUAACUC
UGGCCUGUGUGGUCCCAAGCAGCAGCUCUGCAGCAUGCCAGGGCAUCGGGCAGGGAAUGCUCAGCCUCCUCUCCUUGCUCCAGUUCUUCAUCCUGCU
GAAUGGGGCUGAGCGAAUGUAAAUGCAGUUCAUUGGUCUCCAACAUUUCAUCCUAAGCCAGCUAGAAGUAUGCUGCUGUGCCAGGUGGGAGCAGGAC
AGGCACUGGGUAAGAAUUGGCAGCCUGCUGAGAAACACCCCAGGAGUUUGUCCCUUAUGGGCAGGGAGAUUCCUGUCUGUGCAGCUGGCAGCAUGCA
GACCCUUCAAAUGAUACCUGAGAAGGGGCUUGAGCACACAUAGCUGGAUAUUUAGGCUUACACAAGAGAACACAGCAAUUAAAUACGGUUUUUGUGC
UAUUUCCUGG

>*GNB2L1*, NM_001004378
GGAUGUGGUAAAAACACACAGAUUUUAUGCACAAAACUCAAUAAAAGAGCGCUCUGUUGAGCUCAUGGCUGCAUUAUAUCCCA

>*EIF4E1B*, XM_015293879
GAUUGGGGUGGUGGGAGCAUGGGGGGAGCGGGACCCCCCUUGCCCGACCCCACAAUGAAUGGCUUCAGCUCA

>*SRSF7L*, NM_001031368
AGGAGGAACACACAUCCUGAAAGAAUGGAUUCAAGUUAAAAGGUUAAAGUUUAAGAAGAAGAUUUAUUUUGUUUACAUUAUUAUAAGGAUUUUGUG
GUGUCUUUAUAAACGCAAGGGCUUAGUCCUAGAAGGUUGUUUCUAUUGCCUAACAACAGGCAUGAAUUUGGAUCUUUUAAAAGUAGACUAAUGCAAA
ACUCCUUUGUUGUGUUAAUGUUCAGUGAUCUGGAAAUAUAGGGCUUUUUGUAUUUGGUGCAUUGGAAUAAACAGUGCAUUUCUAACUAAAAAAAAAA
AAAAAA

>*SRSF3*, NM_001195554
GACUUUGAGAAGGAGCUGUGUACAGGAAGUCGUUAGAUCUGAGUACGGGGAGUAUGUACAGAACAUUGUUUUGUUUUGGAAUUUCAUAAAGCUUGGU
GCAUUUUUAAAAUGUUUUAGCUGUUGAACUUGCUUUGUUCCUUGUAUCUUGUAACAGGUGGCAAAUGUAAAGAUGUGAAUCUGUAUAUGGUUCUAAG

CAGAUCAUAUGAAUUGUAAUAUUAAUCACUUUUUACAAUGUACCCUUACACUUAAUUGUUUGUGUUGAAUGGUUACCAGUUUGUCUUGCAUAGAUAA
UGUUUCUGAUCUUGACAUUGUGCAGUAUGUGAUUGCUGGGGUUCAGCCUGGGGGGGGUGUGUGCAGCAAAGUUGUUUGUUGCAGAGUGGUGGCUGUG
AGUGGUGGUGGUGCAGGAAAGGUGUAGGUGCUGCAUAGUGCAGUACCAGCAGUCACAUUGAGCCAGCGGUGUGAAAGGAACGAGCAAGCUGCUGUUA
CCCACAGCUGUAGUUCUGACUUGGAUUACCUGGAACAGAGCUCCUUUGUAUUAUGAUAUACAAUUUUAUAGCUUGUAGGGUCAGUUAAUUUUGUACU
UCAUGUGACUAGAGUGCUUAAAUAGAUCUAGCUUGACCAUCUAUUCCAGGACCUCUGAAUAGACCAAAUACACAGCAGAACUGACAACUGGAGUGGA
UGUGUUGACUUUGUAAUGGUACAUUGUAAAAGUUGUGAAUUAAACAUUUCUUUACUGUACAAGAAUUUUCAUGUCACUUGUAAAAUGUCUUUUGUGA
GAUCCUUGGGAUUAAAGUUUUGGUUACAACUUGU

>*RBM44*, XM_025152392

GUAUUCUAGCCUACAAGAACACAGGUGUGAAUUUAUCUAGCUGUGUAGCCUGACUAGGAGAACUUCCUAGGAACCUCUUCCUGCAUAAAAAGUAAGA
GAAUGAAUCACCAAAACAACAAUUUCUAAAGAUUAACUGUUGCUGCAGAACGUCUGUAAGCAGAGGAGCUUGCUAGCUCUGUGCCCUUAUCAAACUG
GGUACGACGGGGGGCUUUAUUAUGCAGGUUGUAUUGAUGUACUCAGUGGAGUACGUUAUCAAUGUUCUGGGCAAAGCUGACAUCUAAUGGCCAGUUC
CUGUGUAGCAUUUCACAGAGGAAUUCCAGUACAGGCUCAAUACUGGUUUAAAACCUGGUGAUUUCUUCUGUUGUAUGGUAUUUAUUUCUGGUGUAGA
UAUUGAACAGAAUAAGUUUGUACGUCCUCUAGUUUUUGCAUGUUGAAGGACUUCGUAGACAGUUCCUUUCUUCUACUUGCUUGCCUGGAAAUCUUGC
AUUGUUUUCUCCCUUUGGCAGAGUUAGCCCAGAGUUGGACAUCUCAAUGUUUAUACAUCUGUAUAUUUGUUAAGAGGGAAGUUAGGCCUGUGCUGAA
AGCACCAUCGGGAAACGCAUGGAGCCCGUUCACUUCUAAAUGGUGACUUGAUGACUUUGAAUUAACUCGGGUGAUUCACAAAAUUACCUCUGGAUGU
GAUCUGUUACACAAGGACUAUUUUAUUUUAUCGUAAUAACUUUUCUGUGCAGUUCUCAGGGCUGGGAAAUGUCGCUCUUGUGUGCAAACUUCUGCCU
UCCAUAGCAAAAGCAGUACAGCCUCUAGUGGUGCUCUGGGAUGCUGACAUUGCUGAUGUUUUGUUUCACGUUACACGUGUUCAGGCCAAACUUAAUC
UGUUUUUACUUUUUCUUUCUUUCUAUGUAGAUGAUUUAAUGAAGAAUUGAACUCUUCACAUGACAGAUUUUUGCAGUCCAGCUCGCUCUGGGAUUGU
UCUGUAUUUCUUGUUUGUUUCAUUUUGGUAGGGCUUCAUGAACACUCGCUUAGCACUGCUGUAUGGUUUGCAGUUCUGUUUGACAGAACAUAGCCCA
AAGGUCUUUUGAGAGUGGCUUUUUGUGCAGGCAGAUGUUAAGUAAUUCUUCCCAGUUUUGUUGUUCUGUGUAGUUCAGUGUACUUCUCUGAGGUUGA
AGGCUCCCAUUUCAGCACAGUGAGUCUCCUCUCAUAGCAGCAGGACUUCUCUCUGCUGAAGCACACAACCCCCGGCUCCUGGUGCCACUCCGUGUCA
GGCACGGUGAUAGCUGCAGUGCUAACAAACAGCCAGCAUGCUUACACGCUUUCCAGAUGACGAUUCCAAAUAUUGUGCAGAAAUGUCAAGUAUCCUU
UUCAUACAAGGCAGGGGAGGGAGAGGCAGCAAGGGCCGGCUUGGAGAACACACAGUGCUGGCGGUGUGGUUACGUAAGCCCUGUGCUGCAGUGGCUG
CUCCGAGCAGAGCUGUGCCCUAGAGAGCUGGGCCUCUAAGCCUCCUUCGGGUCCUAUAGCACAGCACAGGGGAAAGCCUCUCCCACCCAGACUCUCC
CUGAGAUCUGCUUUGGGUUGAUACGGGAGCACUCAGCCUUUUGUUUGCCUGUGGAGCUGCUCCCGGCGUUCUACAUCCUCUUCUGGCCAUGGAAGGA
GAGGCAGCUUAGGUGUGUGCCUGCCGUGAAGCUGAGGGGAGCUAGCACUGAUGGUCGCUGAUAGGCGUGUGCUGUCAUACCACACCUGCAUCGCUGU
CCACCUCUGUCCCUUGGCUGUGUUUUGCCCUAGCUCCUGGCUGCCCGUGCUGCUGAAAGCAGCCUUACCGUGAAUAAAGGUUGAGAUGCUUUCUUCA
CA

>*RPS6*, NM_205225

AGAUGUACAUGAUACUGAAAAUAAAACCCUUUUGUGGUUAAAUUUUACUGUGAGACUUCCAGUGAAUAUAUUUCCUGGCUAUGUCUUAAAAUAAAUG
GUAGUCCAGACUAAAAAAAAAAAAAAAAA

>*DZIP1L*, XM_015277142

GCUCAACAUGCCCCAGAGCCAGCCACCCCACCAGUAAGCUCCAGGUGCCUGAAGUGACCCGGAGGCCUUCCUGGAGGACCUGGCCACAAGUCCCAGU
GGCUGGUGACACACAAGGGACCAACACCUUGGUUACCAUUCAGCCAAGCACCGUGCAAAGGGACACUUGGUGCUAAUGAGGAAAAGGGAGUCCUCUG
CUAGCAGCCACUGGAUGGUGCCACAAGACCAGGAACGAGCUGCAACAGCAUCCUACAGUUGCAGGAGCCUUCAGAGCCCCACCUGCAGUCCCUCCUC
CCCUUCUUGAAUCAGUGGGAACCACGACCCAAUGGAUGACAAUCUGCACAUACACAACACCGAGCAAGGCUGGCUCUGUUUGUAAUGCCCAUCGGGA
GAAAUGAAGCCAGUUUUCCAAACUGUAUACUUUUCUAGCAUUCUUGCUUUUCCAUUUUUUUGGAAAAAAAACAACCUUCUCAGCUGCUCCUCUGUUU
UCAGCACAUUCCACCUUCAGCUCAAACAUAUAUAAUUUCCUAUUACUAGCUGCAAACAUUUGCAAGCCCAUUUACGUGGCAGGGAUAAGGAAGCCCA
GACAGGUUGGGCAGAAUAGGGUGAAUUCCUUUAAGUUGAUCAAAUGGCAAAAGUGCUUUCCAGAUAACAGUCUGCACCAACACAUGUCCCCAGGGAU
GUAUGCACUCAGCUGCGUGCCUAUGCACUCCCAUAUAGUCUGUCUGUGGCUGCAUACCUUAAGAGGGAAACGUUCCCAUUUUAAUCCUUCAAGUUGA
AAUAAGAGCAGUACCUGGUCUGUAUUAUUUGUUACAGUGCUACUGCCAGAUGUGUUAUUUCAUGGGUCACUUAACAUAUGAUUUUCUUUUGGGUGAA
CGUAACCCGUAGUGAGCGCUGCUUUUAAUGAAUUAAAUCAAGAUGGGAUGUUUUCACCUCA
