# Supplementary material for: Whole-Transcriptome Sequencing-Based Analysis of DAZL and Its Interacting Genes during Germ Cells Specification and Zygotic Genome Activation in Chickens
Source: Int J Mol Sci. 2020 Oct 31;21(21):8170. doi: 10.3390/ijms21218170 (PMC7672628; doi:10.3390/ijms21218170)
Supplement: Supplementary file 1 [file ijms-21-08170-s001.zip › Figures S2-S4_Final.pdf]

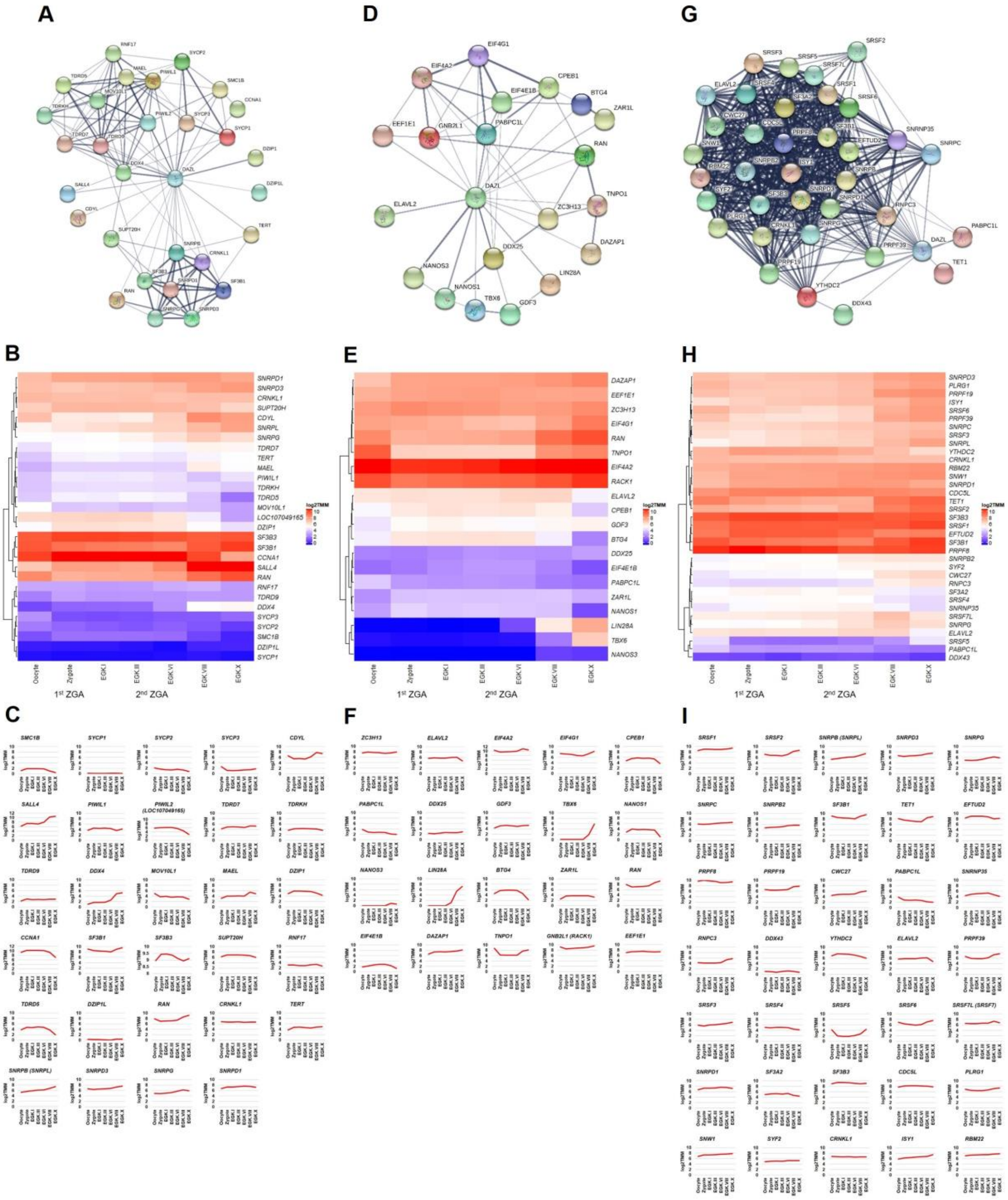

Figure S2. Interaction network and intrauterine embryonic expression of *DAZL* interacting genes identified in the chromosome and/or DNA replication associated (A–C), mRNA and/or tRNA biogenesis (D–F), and spliceosome complex (G–I) categories. The confidence-based direct interaction of *DAZL* with genes identified in these categories are prepared using the STRING database (A, D, G). The expression patterns of *DAZL* interacting genes (in these categories) in the chicken oocyte, zygote, and Eyal-Giladi and Kochav (EGK) stage intrauterine embryos (EGK.I to EGK.X) are examined using the WTS data. log2 TMM-normalization is used to better visualize the gene expression through heatmap (B, E, H) and line graph (C, F, I).

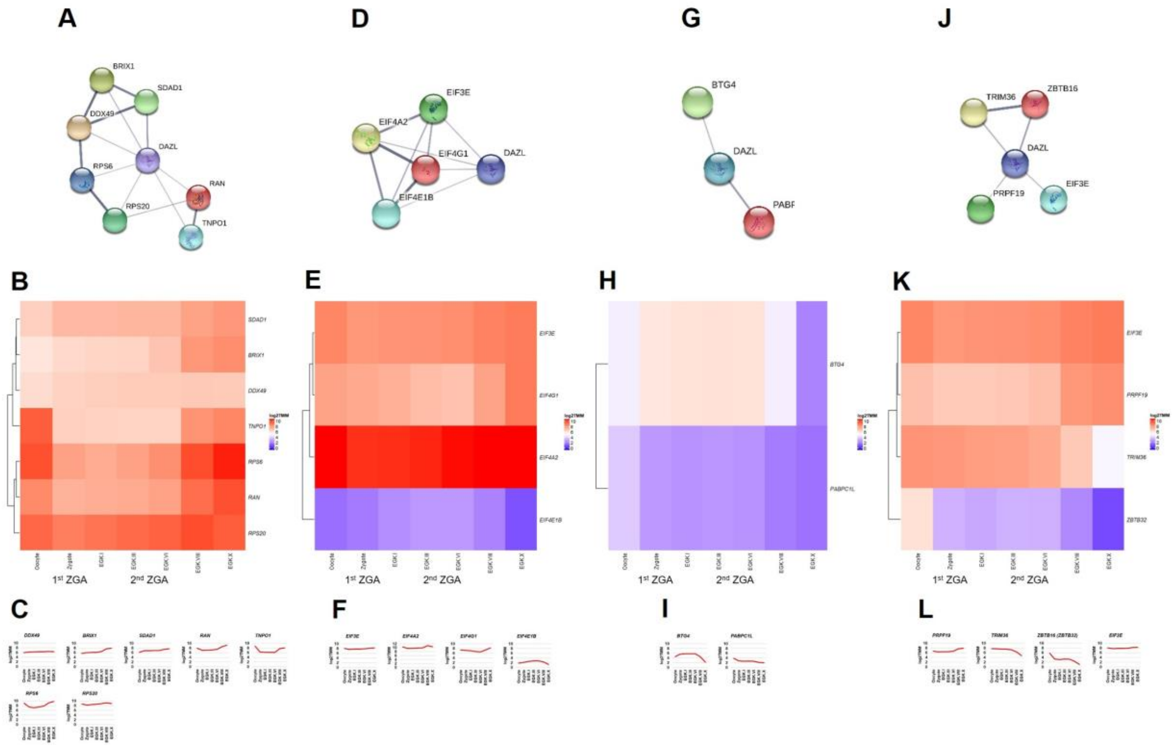

Figure S3. Interaction network and intrauterine embryonic expression of *DAZL* interacting genes identified in the ribosome biogenesis (A–C), translation factors (D–F), RNA degradation (G–I), and ubiquitin and/or proteasome systems (J–L) categories. The confidence-based direct interaction of *DAZL* with genes identified in these categories are prepared using the STRING database (A, D, G, J). The expression patterns of *DAZL* interacting genes (in these categories) in the chicken oocyte, zygote, and EGK stage intrauterine embryos (EGK.I to EGK.X) are examined using the WTS data. log2 TMM-normalization is used to better visualize the gene expression through heatmap (B,E,H,K) and line graph (C,F,I,L).

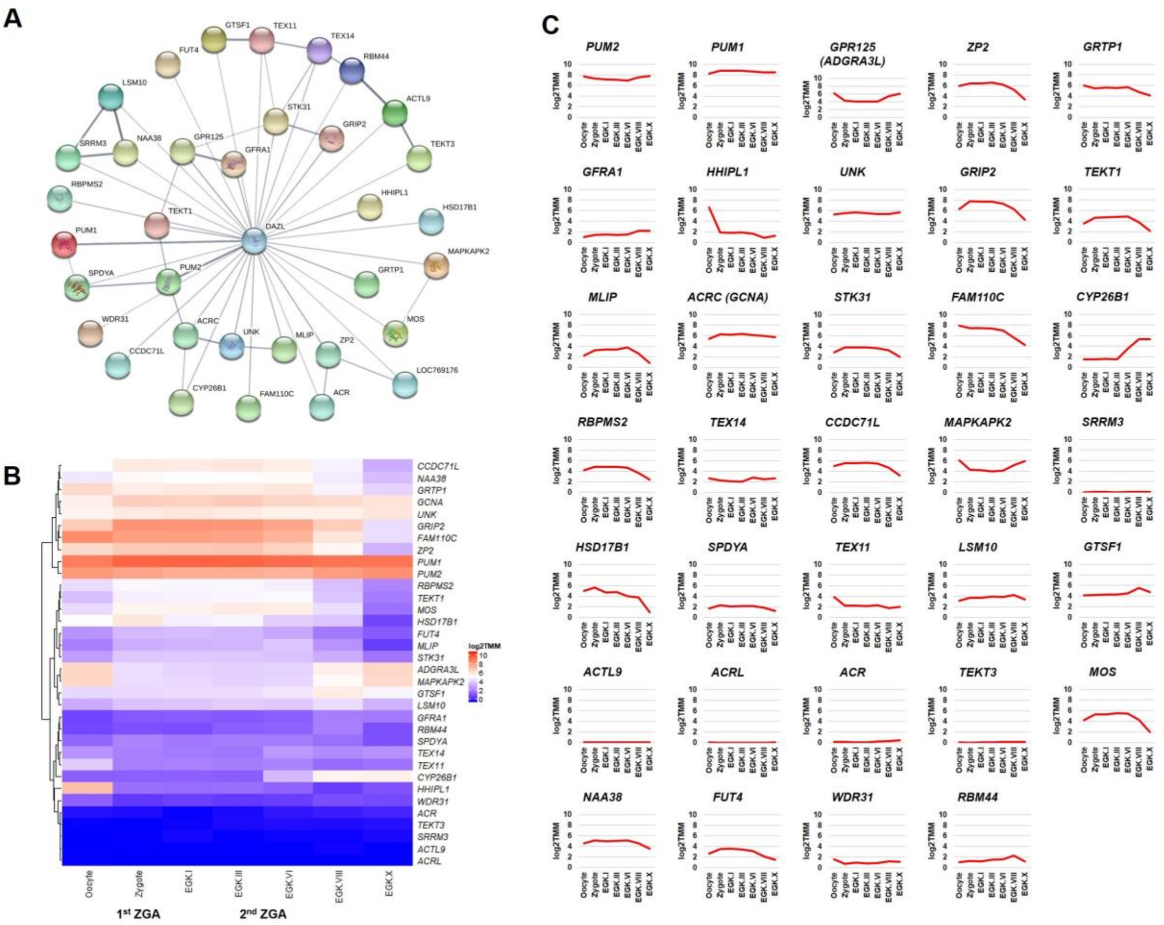

Figure S4. Interaction network and intrauterine embryonic expression of *DAZL* interacting genes that are not identified in the gene ontology terms discussed in this study. The confidence-based direct interaction of *DAZL* with these genes is prepared using the STRING database (A). The expression patterns of these *DAZL* interacting genes in the chicken oocyte, zygote, and EGK stage intrauterine embryos (EGK.I to EGK.X) are examined using the WTS data. log2 TMM-normalization is used to better visualize the gene expression through heatmap (B) and line graph (C).
